# Supplementary material for: Effectiveness of radiofrequency and exercise-based rehabilitation on symptoms associated with pelvic floor dysfunction in breast cancer patients: A study protocol
Source: PLoS One. 2025 Aug 29;20(8):e0330156. doi: 10.1371/journal.pone.0330156 (PMC12396660; doi:10.1371/journal.pone.0330156)
Supplement: S2 File — (DOCX) [file pone.0330156.s002.docx]

**S2 PFMT Programme**

**Physical Exercise Programme focused on the pelvic floor.**

The first 4 weeks will include one day of CORE and pelvic floor training in clinic and individual session to ensure understanding of concepts and correct performance of the technique. After this, the therapeutic exercise protocol will be based on proprioception, mobilisation and activation of the structures responsible for the CORE, isometric work, voluntary activation of the CORE muscles, pelvic floor and synergistic muscles such as the gluteus. The work will be both static and dynamic. The exercises will be performed in resisted exhalation and apnoea to facilitate the activation of the lumbo-pelvic complex, as previous programmes for patients with abdomino-pelvic dysfunctions have already demonstrated their effectiveness.

| ***Ejercicios*** | ***Descripción*** | ***Intensidad y frecuencia*** | ***Ilustraciones*** |
| --- | --- | --- | --- |
| **MONTH 1, Day 1** | | | |
| Individual session in a cabin, where anatomical and breathing concepts will be explained and how to carry out the exercises will be explained, checking their correct performance by means of a digital test. The number of sustained and rapid contractions and apnoea time will be determined according to the initial assessment. | | | |
| **MONTH 1, Days 2, 4, 6, 8** | | | |
| **Pelvic floor and transverse floor activation.** | Face up, legs flexed with heel support and dorsal flexion of the ankle. In resisted exhalation, self- elongation and activation of the transversus abdominis and pelvic floor. | 10 breaths. | 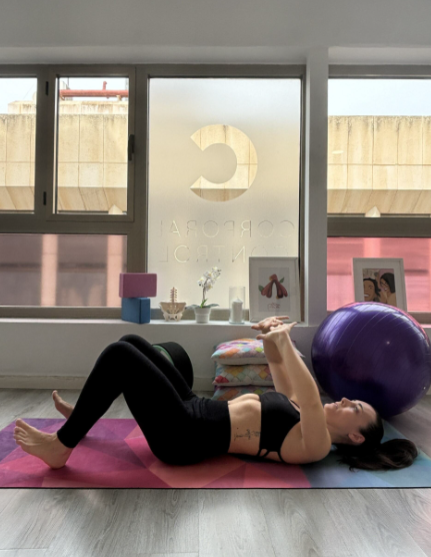 |
| **Gluteal bridge.** | Face up, legs flexed with heel support and dorsal flexion of the ankle. In resisted exhalation, self- elongation and activation of the transversus abdominis and pelvic floor together with gluteal elevation (gluteal bridge). | 10 repetitions. | 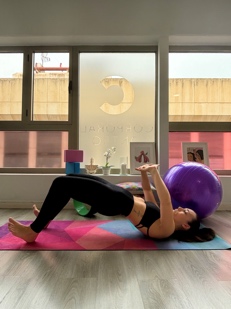 |
| **Gluteal bridge with alternate leg extension.** | Face up, legs bent with heel support. In resisted exhalation, self-elongation and activation of the  transverse and pelvic floor and lifting of the buttocks (gluteal bridge) with unilateral leg stretch. Same exercise with the other leg. | 10 repetitions. | 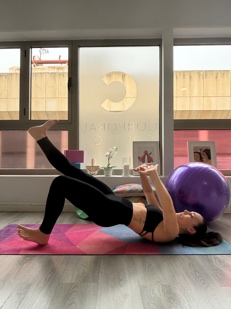 |
| **V-bend abdominals with ball.** | Face up, legs at 90 degrees with ball between them, hip and knee flexion-extension movement is performed with adductor activation.  This is performed with exhalation on descent and stretching of the legs. | 10 repetitions. | 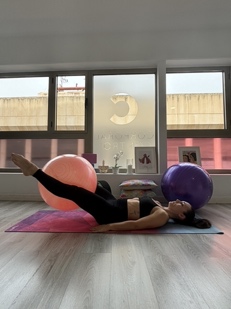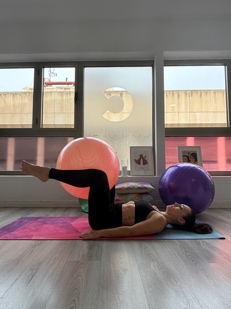 |
| **Leg raise lying on the side.** | On the side with legs straight, activation of the transverse and pelvic floor, perform lifting and lowering movement of the upper leg with maximum amplitude and slow speed and then with minimum amplitude of the movement and maximum speed. | 10 repetitions. | 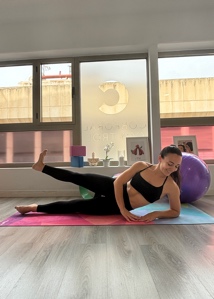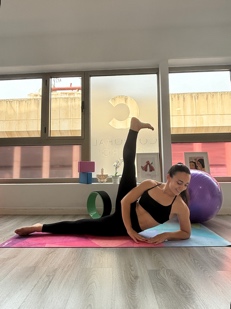 |
| **Leg circling sideways.** | On the side with legs stretched out, activation of the transversus abdominis and awareness of the pelvic floor, perform a circling movement with the upper  leg in both directions. | 10 repetitions. | 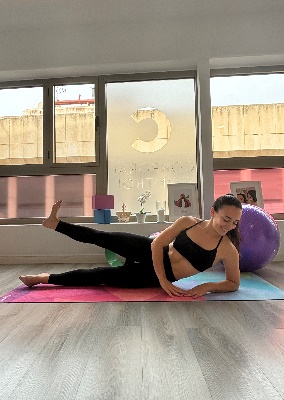 |
| **Side leg flexion and extension.** | On the side, legs aligned with the trunk, activate the transverse and pelvic floor, perform hip and knee flexion and extension movements. | 10 repetitions. | 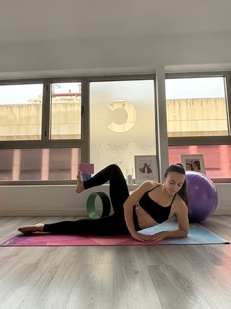 |
| **Sitting contractions on Fitball.** | Sitting on the Fitball, perform pelvic floor contractions with activation of the transversus abdominis, fast and sustained in expiratory time. Do not stay in apnoea. | According to the assessment parameters that we made in consultation. | 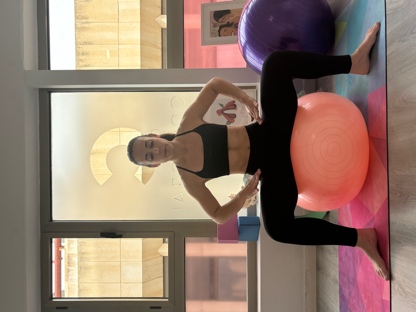 |
| **Pelvic mobility on Fitball.** | On Fitball, awareness and pelvic proprioception movements (perform circles in both directions and infinite circles) to give mobility to the pelvis and relax the CORE and pelvic floor. | 60 seconds. | 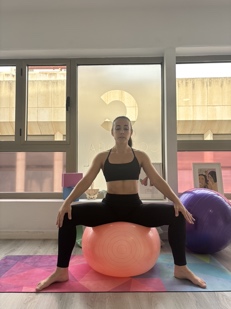 |
| **MONTH 1, Days 3, 5, 7** | | | |
| **Pelvic mobility on Fitball.** | On Fitball, awareness and pelvic proprioception movements (perform circles in both directions and infinite circles) to give mobility to the pelvis and relax the CORE and pelvic floor. | 60 seconds. | 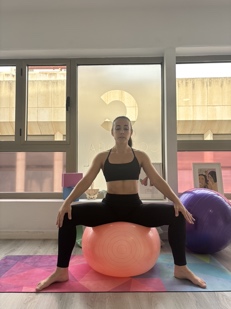 |
| **Pelvic contractions on Fitball.** | Sitting on the Fitball, perform pelvic floor contractions with activation of the transversus abdominis, fast and sustained in expiratory time. Do not stay in apnoea. | According to the assessment parameters that we made in consultation. | 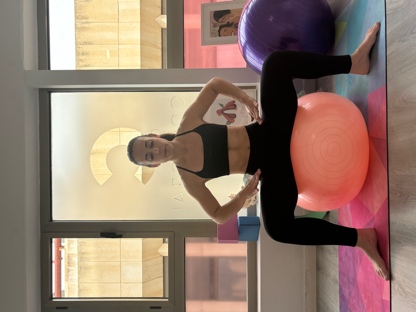 |
| **Self-stretching on Fitball.** | Sitting on the Fitball with heels supported and dorsal flexion of the ankle. After 2 full breaths, stay in apnoea and self-stretch, growing with transverse and pelvic floor activation. | Maintenance of 10sec. | 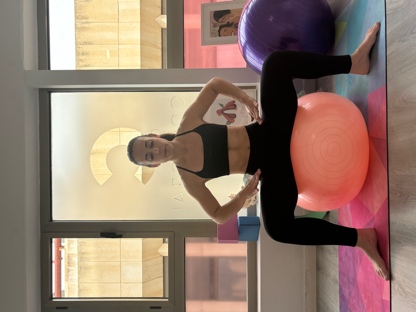 |
| **Self stretching on Fitball with alternating leg lifts.** | Sitting on the Fitball after 2 full breaths, stay in apnoea and self-stretch, growing, with transverse and pelvic floor activation with  alternate leg raises. | 10 sec. maintenance | 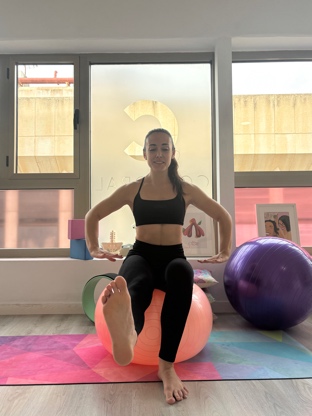 |
| **Quadruped pelvic scales.** | In quadruped movement of pelvic scales with transversus activation and pelvic floor awareness. | 10 repetitions. | 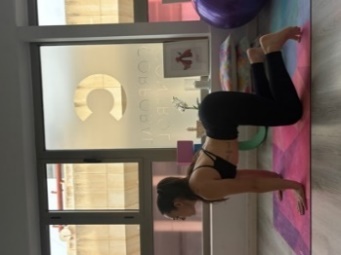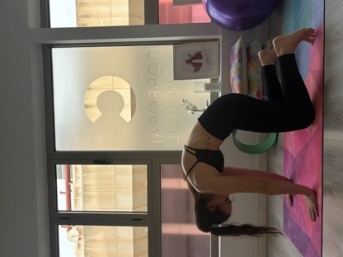 |
| **Lifting of opposite limbs in quadruped.** | In quadruped position, raise one arm and one contralateral leg in resisted expiratory time. Alternate limbs. | 10 repetitions. | 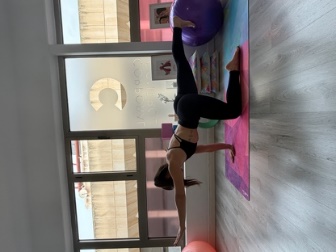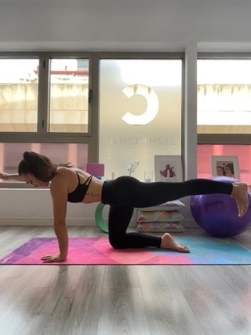 |
| **Hypopressives and self-stretching in quadruped with alternating arm flexion and leg .** | On quadruped, perform hypopressive exercise (after 2 full breaths stay in apnoea, self-elongation and activation of the transverse and pelvic floor and hold for 10 seconds) with flexion of the arms and stretching of one leg.  Repeat with the other leg. | Maintenance of 10sec. | 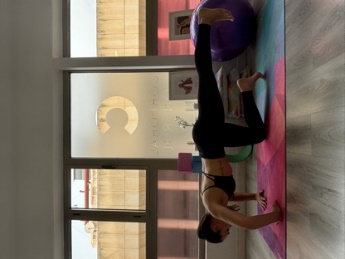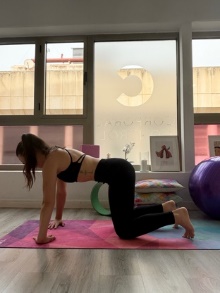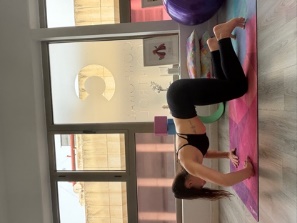 |
| **Mohammedan stance.** | In quadruped position, pelvis mobilisation and  dynamic stretching, ending with the posture of the Mohammedan and stretching of the posterior chain. Sit on your heels, rest your head on the floor and stretch your arms in front of you. |  | 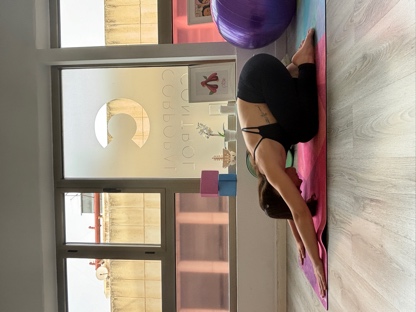 |
| **MONTH 2, Days 9, 11, 13, 15** | | | |
| **Pelvic mobility.** | Standing, pelvic awareness exercise. Make infinite circles in both directions with the pelvis, hands on hips. | 60 seconds | 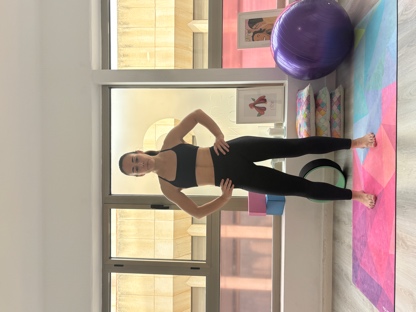 |
| **Pelvic floor contractions.** | Standing, neutral pelvis position, rapid pelvic floor contractions, then held while exhaling. Do not stay in apnoea. | Adapt the number of repetitions according to the assessment made in the  consultation. | 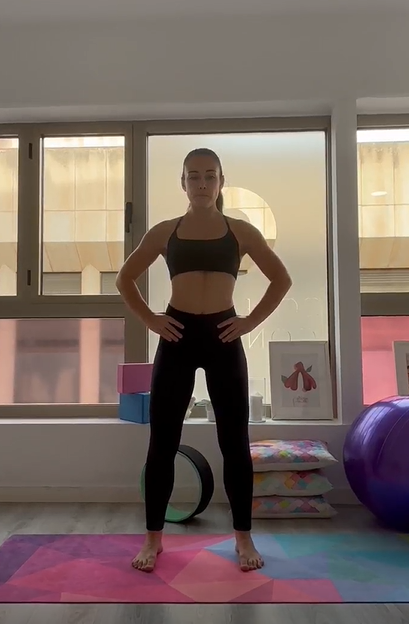 |
| **Balance and motor control on unstable bases.** | Balance and motor control exercises, with conscious breathing on trunk with stable base, on trunk with unstable base and on roller. | Hold each posture for 60 seconds. | 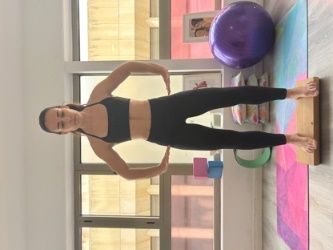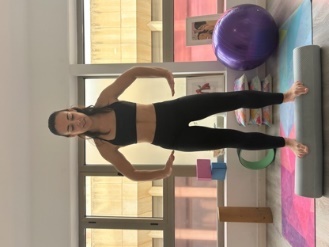 |
| **Deep squats.** | Feet placed wider than hip width apart. Deep squat and raise, activating transverse and pelvic floor. | 10 repetitions. | 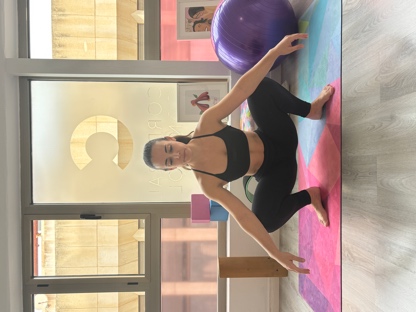 |
| **Isometric squats** | Feet placed wider than hip width apart. Squat held for 3 seconds and raise, all while activating the transverse and pelvic floor. | 10 repetitions. | 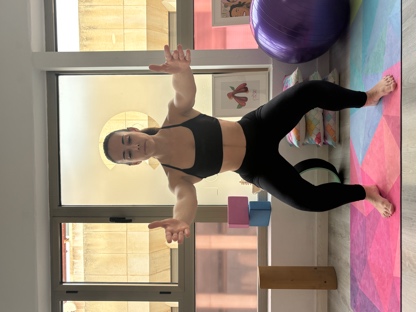 |
| **Alternating strides** | Standing, arms outstretched in front. Forward lunges  alternating legs. Activate transverse and pelvic floor. | 10 repetitions. | 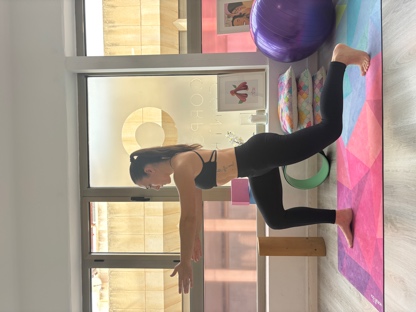 |
| **Self-stretching on the ground.** | Face up, legs flexed with heel support and dorsal flexion of the ankle. In resisted exhalation, self- elongation and activation of the transversus abdominis and pelvic floor. | 10 breaths. | 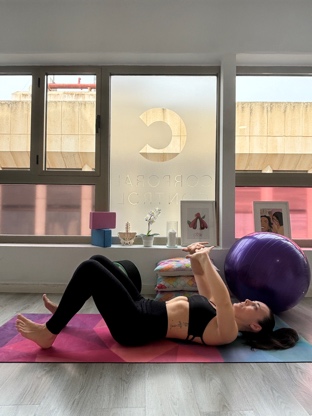 |
| **Gluteal bridge.** | Face up, legs flexed with heel support and dorsal flexion of the ankle. On exhalation resisted self- elongation and activation of the transversus abdominis and pelvic floor together with gluteal lift (gluteal  bridge). | 10 repetitions. | 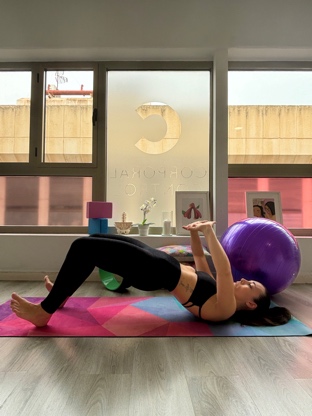 |
| **Gluteal bridge with alternate leg extension.** | Face up, legs bent with heel support. In resisted exhalation, self-elongation and activation of the transversus abdominis and pelvic floor and gluteus lift (gluteus bridge) with unilateral leg stretch. Same exercise with the other leg. | 10 repetitions. | 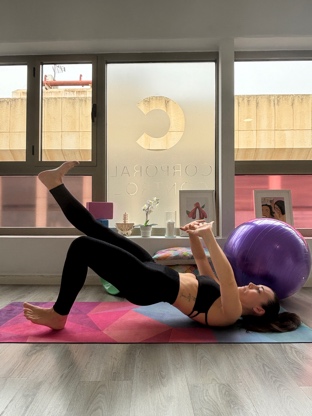 |
| **Activation of adductors with a ball.** | Face up, legs extended at 90° with a ball between them, hip and knee flexion and extension movement with adductor activation. Exercise with normalised breathing. | 10 repetitions. | 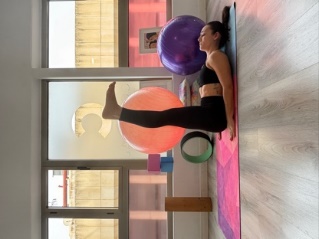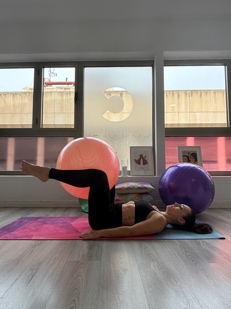 |
| **V-squat alternating bent- leg stretches with ball.** | Face up, legs at 90° with ball between them, hip and knee flexion-extension movement is performed with adductor activation. This is performed with exhalation on descent and  stretching of the legs. | 10 repetitions. | 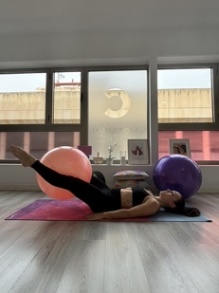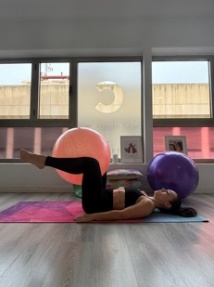 |
| **Active stretching and relaxation and body awareness session.** | Stretching arms, legs, neck, lower back, standing, sitting, calmly, paying attention to your breathing. Return to calm. | 5 minutes. | 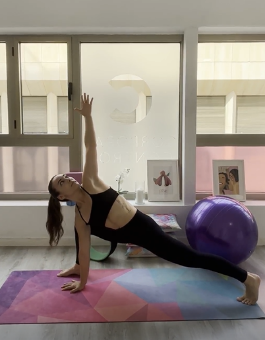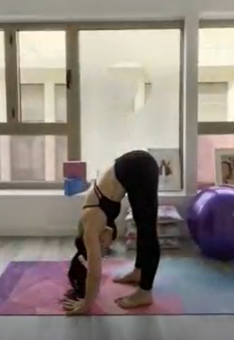  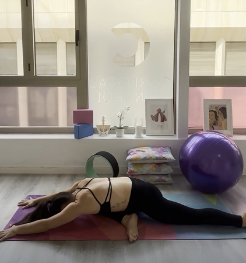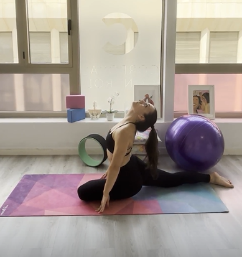 |
| **MONTH 2, Days 10, 12, 12, 14, 16** | | | |
| **Awareness and proprioception.** | Face up, awareness exercises and pelvic proprioception, performing sustained and rapid contractions. | According to initial patient assessment. | 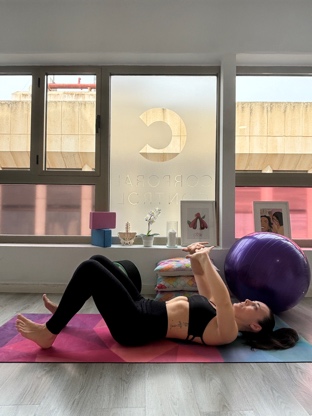 |
| **Self-elongation.** | Face up, legs flexed with heel support and dorsal flexion of the ankle. In resisted exhalation, self- elongation, growing and activation of the transversus abdominis and pelvic floor. | 10 breaths. | 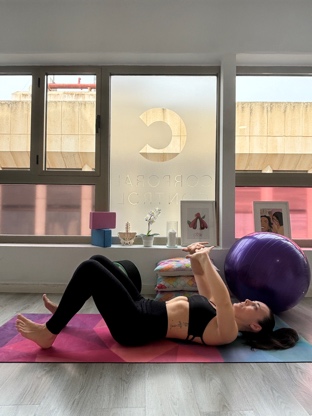 |
| **Gluteal bridge.** | Face up, legs flexed with support of heels and dorsal flexion of the ankle. In resisted exhalation, self- elongation and activation of the transversus abdominis  and pelvic floor together with gluteal elevation (gluteal bridge). | 10 repetitions. | 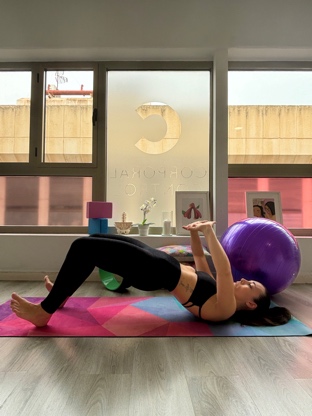 |
| **Gluteal bridge with alternate leg extension.** | Face up, legs bent with heel support. In resisted exhalation, self-elongation and activation of the transversus abdominis and pelvic floor and gluteus lift (gluteus bridge) with unilateral leg stretch. Same exercise with the other leg. | 10 repetitions. | 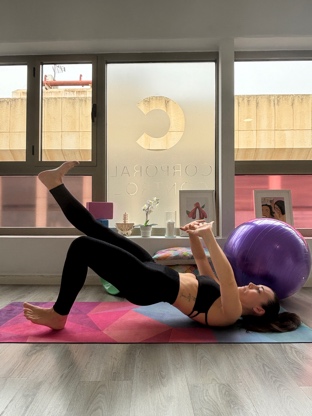 |
| **Leg raise lying on the side.** | On the side with legs straight, activation of the transverse and pelvic floor, perform lifting and lowering movement of the upper leg with maximum amplitude and slow speed and then with minimum amplitude of the movement and maximum speed. | 10 repetitions. | 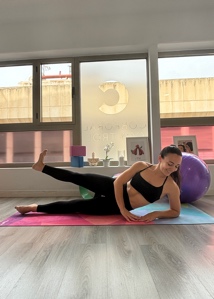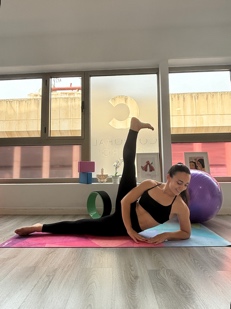 |
| **Circumduction of the leg sideways.** | On the side with legs stretched out, activation of the transversus abdominis and awareness of the pelvic floor, perform a circling movement with the upper leg in both directions. | 10 repetitions. | 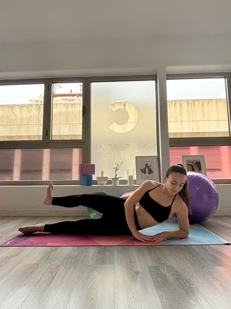 |
| **Side leg flexion and extension.** | On the side, legs aligned with the trunk, activate the transverse and pelvic floor, perform hip and knee flexion and extension movements. | 10 repetitions. | 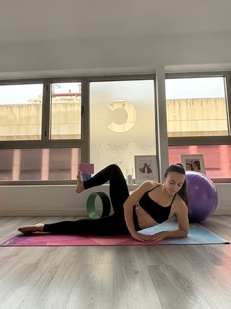 |
| **Hypopressives and self-stretching in inverted dog.** | On the quadruped perform hypopressive exercise (after 2 full breaths, go into apnoea and activate transverse and pelvic floor and hold for 10 seconds) with  push-up and leg stretching  (mountain or dog yoga position inverted). | 10 seconds. | 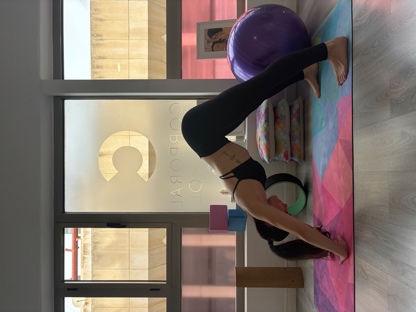 |
| **Hypopressives and self-stretching in inverted dog with alternating leg lifts.** | On the quadruped perform hypopressive exercise (after 2 full breaths, remain in apnoea and activate transversus abdominis and pelvic floor and hold for 10 seconds) with flexion of upper limbs and stretching of the lower limbs (mountain or dog yoga position Inverted) and add one leg lift to the ceiling and hip and knee flexion-extension movement. Repeat with the other leg. | 10 seconds. | 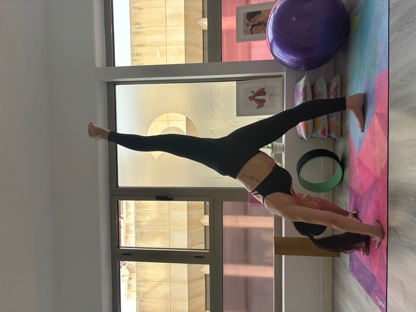 |
| **Front plank with arms bent.** | Front plank on toes and support on forearms. Back and legs aligned, abdominals strong. | 60 seconds. | 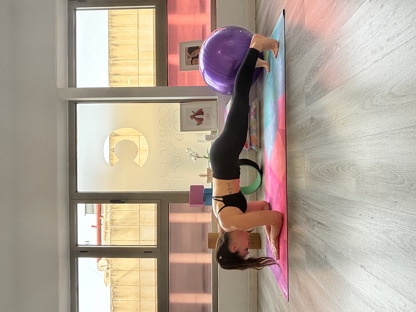 |
| **Front plank with arms outstretched.** | Front plank on toes and support on hands, arms extended. Back and legs aligned. Strong abdominals. | 60 seconds. | 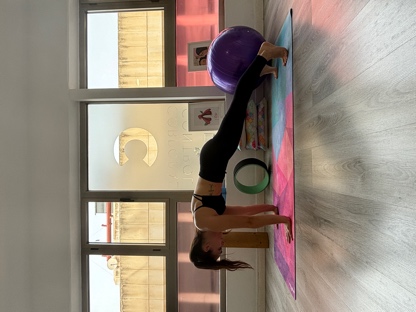 |
| **Side plates.** | Lateral plank, feet together, support on one arm stretched out and the other stretched up. Keep pelvis aligned with trunk. Repeat on the other side. | 60 seconds with  30 seconds rest between each. | 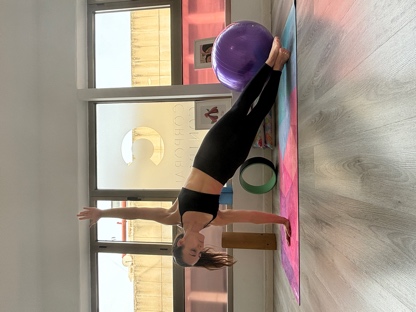 |
| **Front plank with alternating arm and leg raises.** | Front plate with alternating elevation of left arm and right leg and their opponents with strong abdominals and pelvic floor awareness. | 10 repetitions (5 on each side). | 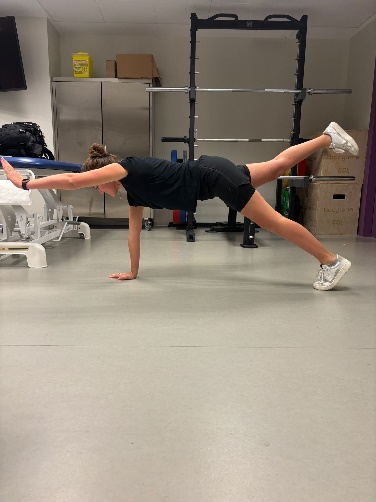 |
| **Front plank - climber.** | Front plank on outstretched arms. Bring the knee to the elbow on the same side, alternating legs. | 10 repetitions. | 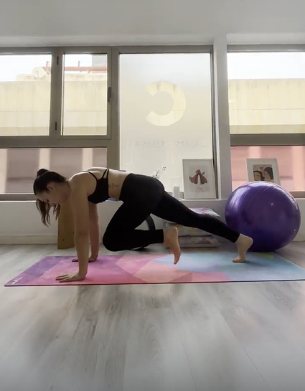 |
| **Exhalation crunch.** | Crunch on exhalation, activating abdominals, head lift with hands behind the head. | 10 repetitions. | 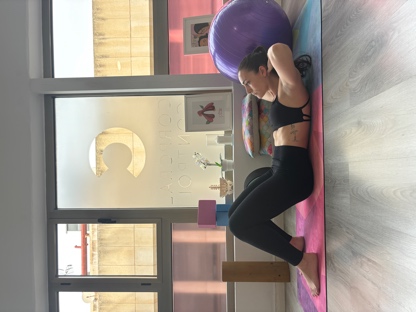 |
| **Exhalation crunch with lateral tilt.** | Exhalation crunch with lateral tilt. Hands crossed behind the head, elbow reaching for the opposite knee. | 10 repetitions on each side. | 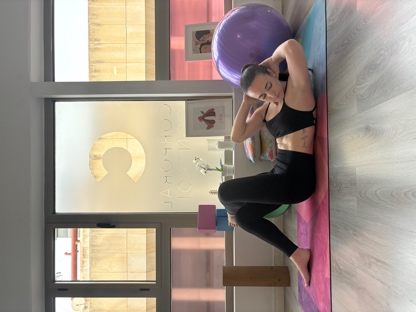 |
| **Crunch with trunk elevation and laterality.** | Legs bent without supporting heels. Trunk upright, contracting abdominals, trunk rolls to each side. | 10 repetitions on each side. | 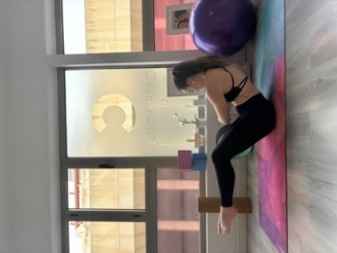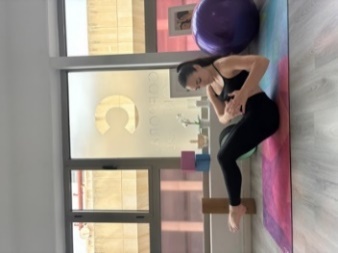 |
| **Oblique.** | Face up, legs bent, resting on heels. Slight elevation of the head and hands reaching for each heel alternately. | 10 repetitions on each side. | 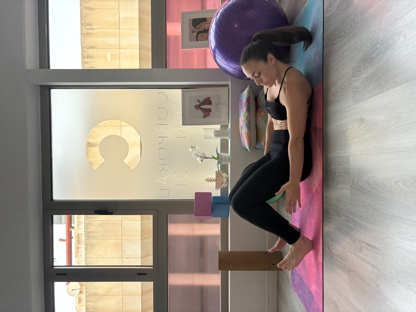 |
| **Active stretching**  **and relaxation**  **and body**  **awareness session.** | Stretching arms, legs, neck,  lower back, standing,  sitting, calmly, paying  attention to your breathing. | 5 minutes. | 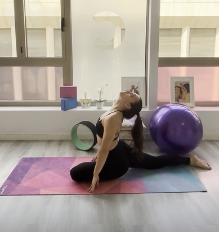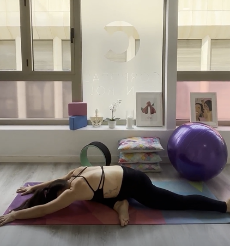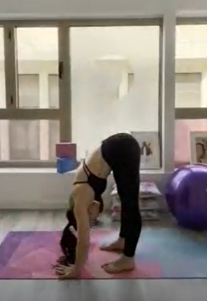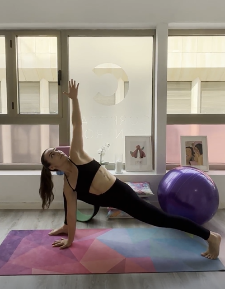 |

During this 3rd month, we will perform mobility, proprioception and pelvic floor muscle training exercises with increased loading through posture and movement. External load up to 60% of your RM will be introduced according to the progression of loads in terms of strength, endurance and health.

| **MONTH 3, Days 17, 19, 21, 23** | | | |
| --- | --- | --- | --- |
| **Gluteal bridge face** | Face up, legs flexed with heel support and dorsal flexion of the ankle. In resisted exhalation, self- elongation and activation of the transversus abdominis and pelvic floor together with gluteal elevation (gluteal bridge). | 10 repetitions. | 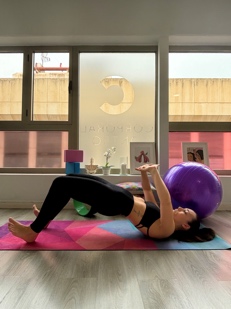 |
| **Gluteal bridge with alternating leg stretching on the back with alternating leg stretching** | Face up, legs bent with heel support. In resisted exhalation, self- elongation and activation of the transversus abdominis and pelvic floor and gluteus lift (gluteus bridge) with unilateral leg stretch. Same exercise with the other leg. | 10 repetitions. | 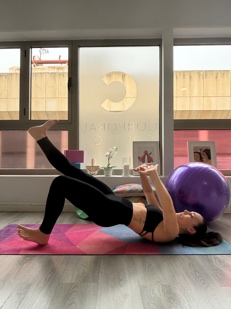 |
| **V-shape sit-ups alternating bent-leg stretches with a ball.** | Face up, legs at 90° with ball between them.  Perform hip and knee flexion-extension movement with adductor activation. This is performed with exhalation on the descent and stretching of the legs. | 10 repetitions. | 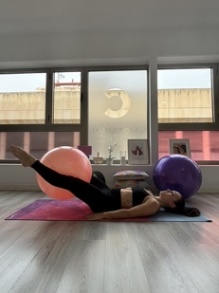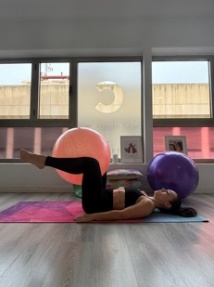 |
| **Front plank with arms bent.** | Front plank on toes and support on forearms.  Back and legs aligned, abdominals strong. | 90 seconds.  Rest: 30 sec | 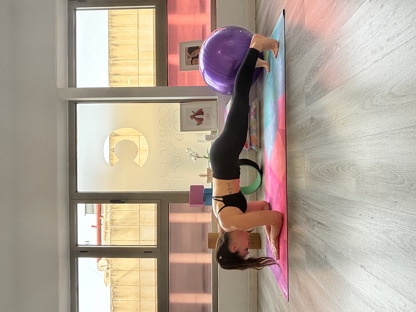 |
| **Front plank with arms outstretched.** | Front plank on toes and support on hands, arms extended. Back and legs aligned. Strong abdominals. | 90 seconds. Rest: 30sec. | 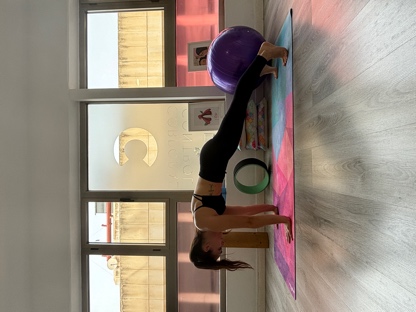 |
| **Lateral plank.** | Lateral plank, feet together, support on one arm stretched out and  the other arm stretched up. Repeat on the other side. | 90 seconds.  Rest: 30 | 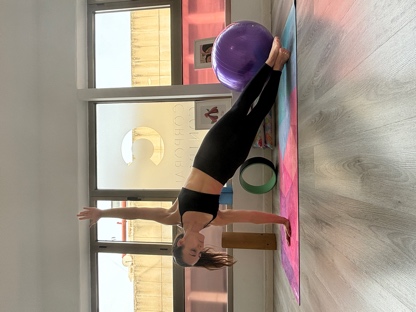 |
| **Front plank with alternating arm and leg raises.** | Front plate with alternating elevation of left arm and right leg and their opposites with strong abdominals and pelvic floor awareness.  Repeat with the other side. | 10 repetitions (5 on each side). | 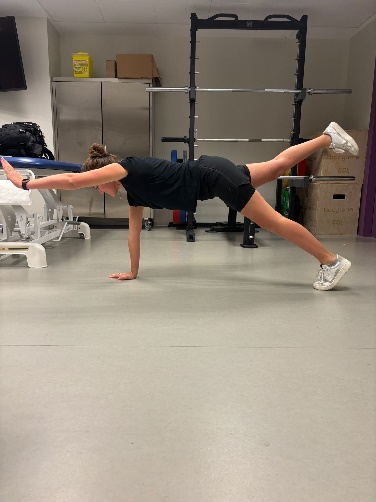 |
| **Front plank - climber.** | Front plank on outstretched arms. Bring the knee to the elbow on the same side,  alternating legs. | 10 repetitions. | 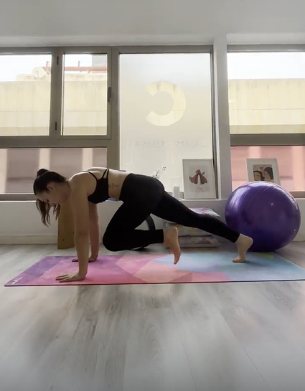 |
| **Active stretching and relaxation and body awareness session.** | Stretching arms, legs, neck, lower back, standing, sitting, calmly, paying attention to your breathing.  Return to calm. | 5 minutes. | 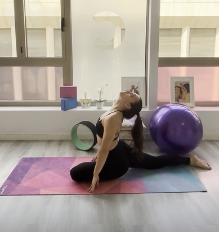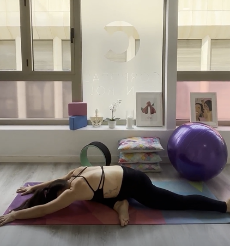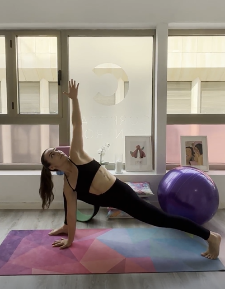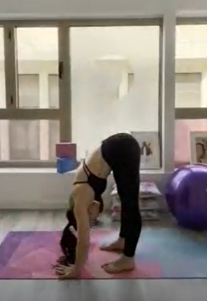 |
| **MONTH 3, Days 18, 20, 20, 22, 24** | | | |
| **Pelvic mobility.** | Standing, pelvic awareness exercise. Make infinite circles in both directions with the pelvis, hands on hips. | 60 seconds. | 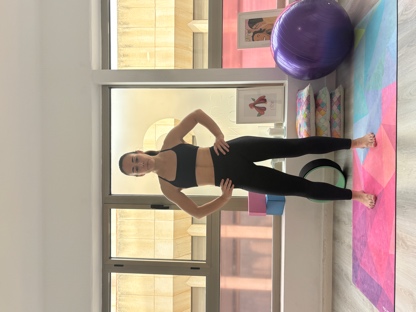 |
| **Pelvic floor contractions.** | Standing, neutral pelvis position, quick pelvic floor contractions, then  hold and exhale. Do not stay in apnoea. | Adapt the number of repetitions according to the  assessment made in the consultation. | 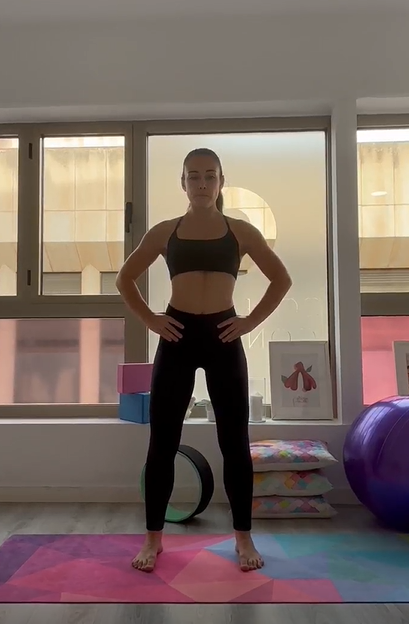 |
| **Balance and motor control on trunk with stable base.** | On the trunk, with the base stable on the floor, do ketellball push-ups and then rotate the ketellball around your abdomen. | 10 repetitions of each. | 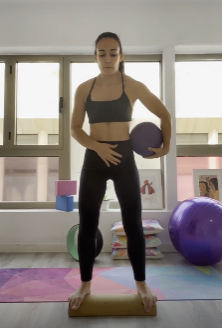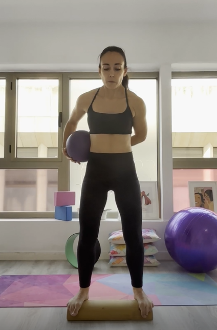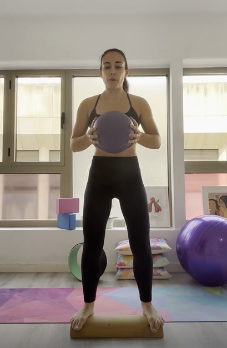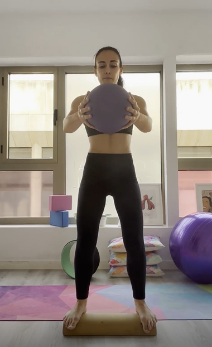 |
| **Balance and motor control on trunk**  **with unstable base.** | On the trunk, with the base unstable on the  floor, do ketellball push- ups and then rotate the ketellball around your abdomen. | 10 repetitions of each. | 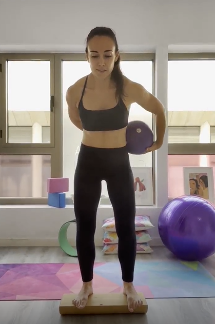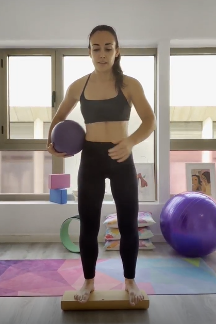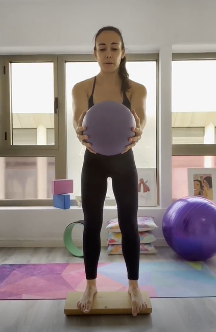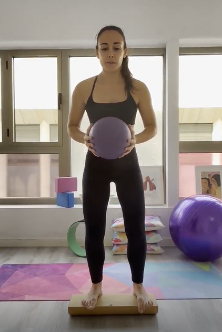 |
| **Balance and motor control on a roller.** | On the roller, do ketellball push-ups and then rotate the ketellball around your abdomen. | 10 repetitions of each. | 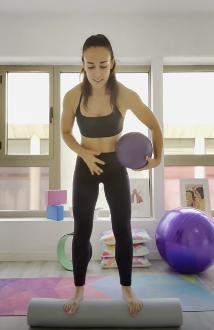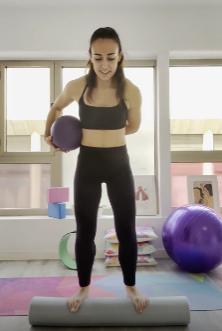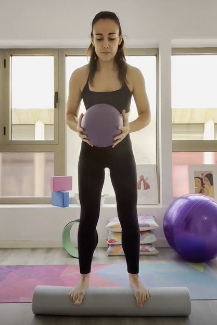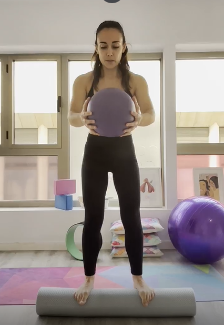 |
| **Deep squat with ketellball.** | Feet placed wider than hip width apart. Hold ketellball with arms extended in front. Deep squat and raise, activating transverse and pelvic floor. | 10 repetitions. | 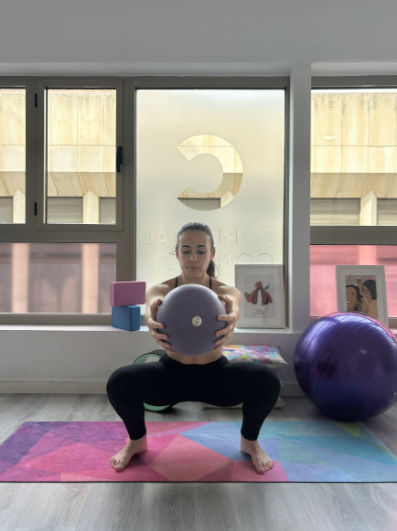 |
| **Isometric squat with ketellball.** | Feet placed wider than hip width apart. Hold ketellball with arms extended in front of you. Deep squat, hold for 3 seconds and raise, all activating transverse and pelvic floor. | 10 repetitions. | 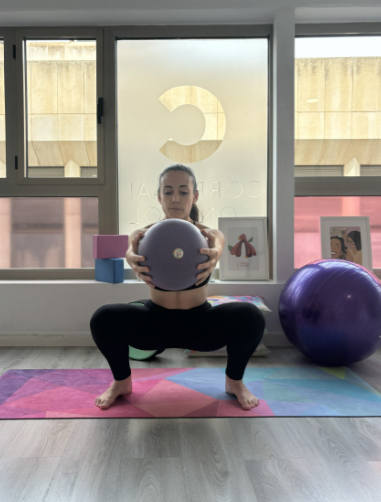 |
| **Alternating stride with** | Standing, catch ketellball with arms extended in front. Lunge forward alternating legs. Activating transverse  and pelvic floor. | 10 repetitions of each leg. | 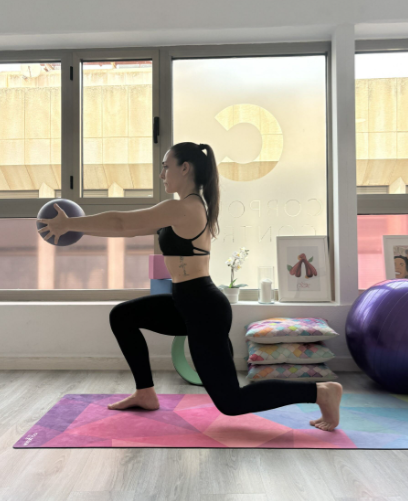 |
| **Hypopressives and self-stretching in inverted dog.** | On the quadruped perform hypopressive exercise  (after 2 full breaths, go into apnoea and activate transverse and pelvic floor and hold for 10 seconds) with flexion of upper limbs and stretching of the lower limbs (mountain or dog yoga position inverted). | 10 seconds. | 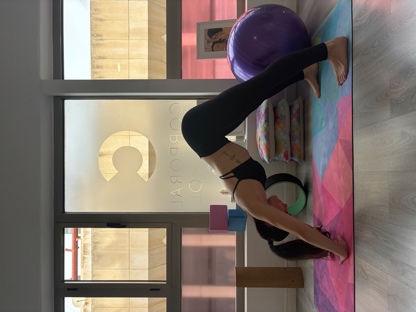 |
| **Hypopressives and self-stretching in inverted dog with alternating leg lifts.** | On the quadruped perform hypopressive exercise (after 2 full breaths, remain in apnoea and activate transversus abdominis and pelvic floor and hold for 10 seconds) with flexion of upper limbs and stretching of the lower limbs (mountain or dog yoga position  Inverted) and add one leg lift to the ceiling and hip and knee flexion- extension movement.  Repeat with the other leg. | 10 seconds with  each leg (20 seconds). | 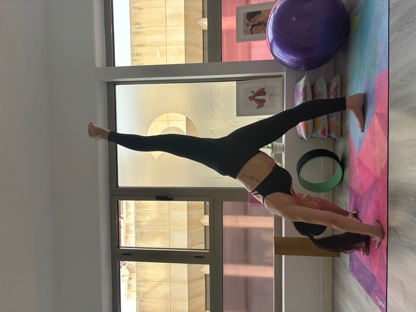 |
| **Crunch with ketellball.** | Lie on your back, support your heels, take the ketellball and raise the trunk a few degrees, activating the transverse and pelvic floor in expiratory time. | 10 repetitions. | 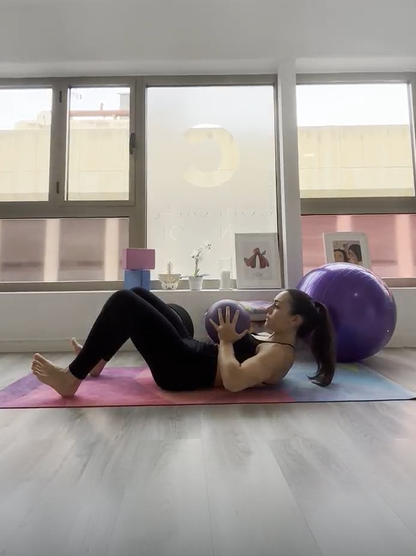 |
| **Crunch with side bends.** | Same exercise, but with 2 ketellballs (1 in each hand) or weights, and instead of raising the trunk, do inclines by bringing the ketellball closer to each heel. | 10 repetitions. | 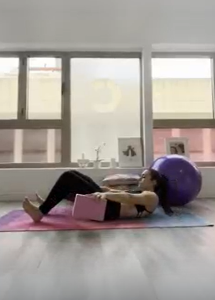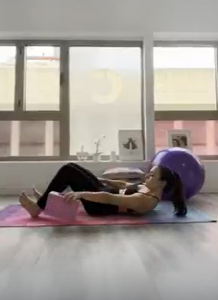 |
| **Crunch with trunk lift and laterality.** | Same position, but place the lateral side of the left foot on the opposite knee. Lifting the trunk with the ketellball, bring the right shoulder to the left knee. Repeat on the other side. | 10 repetitions. |  |
| **Oblique with lift and weight.** | Sitting, with the trunk leaning backwards and without supporting the legs, carry the ketellball  alternately to the left and right. | 10 repetitions on each side. |  |
| **Active stretching and relaxation and body awareness session.** | Stretching arms, legs, neck, lower back, standing, sitting, calmly, paying attention to your breathing. | 5 minutes |  |

During the 4th month, we will re-evaluate the RM for load management. We will increase it to 75% and focus on working and activating the CORE and pelvic floor in a dynamic way, with normalised breathing and introducing impact and fatigue, which are demonstrable risk factors in pelvic pathology, with the aim of automating the abdominopelvic synergy and its competence in everyday situations, as well as physical exercise.

| **MONTH 4, Days 25, 27, 29, 31** | | | |  |
| --- | --- | --- | --- | --- |
| **Gluteal bridge with alternate leg**  **extension.** | Face up, legs bent with heel support. In resisted  exhalation, self-elongation and activation of the transversus abdominis and pelvic floor and gluteus lift (gluteus bridge) with unilateral leg stretch.  Same exercise with the other leg. | 10 repetitions. |  | |
| **"Hip thrust with ketellball** | Face up, knees bent with heels on the floor, lifting toes to the ceiling, slowly release the air while contracting the abdomen and pelvic floor and using the ketellball to lift the  pelvis. | 10 repetitions. |  | |
| **V-shape sit-ups alternating bent-leg stretches with a ball.** | Face up, legs at 90°° with a ball between them, hip and knee flexion-extension movement is performed with adductor activation. This is performed with exhalation in the descent and stretching of the legs. | 10 repetitions. |  | |
| **Abdominals in hip and knee flexion and extension with ball.** | Face up, knees with hips at 90° and a ball between the knees squeezing to activate the inner thigh muscles, bring the feet towards the ceiling and lower the feet to the  floor without touching it. | 10 repetitions. |  | |
| **Front plank with arms bent.** | Front plank on toes and support on forearms.  Back and legs aligned, abdominals strong. | 90 seconds. Rest : 30s |  | |
| **Front plank with arms outstretched.** | Front plank on toes and support on hands, arms extended. Back and legs aligned. Strong abdominals. | 90 seconds. Rest : 30s |  | |
| **Side planks** | Lateral plank, feet together, support on one arm stretched out and the other arm stretched up. Repeat on the other side. | 60 seconds with 30 seconds rest between each. |  | |
| **Front plank with alternating arm and leg raises.** | Front plate with alternating elevation of left arm and right leg and their opposites with strong abdominals and pelvic floor awareness. Repeat with the other side. | 10 repetitions (5 on each side). |  | |
| **Front plank - climber.** | Front plank on outstretched arms. Bring the knee to the elbow on the same side,  alternating legs. | 10 repetitions. |  | |
| **Hypopressives and self-stretching in inverted dog with alternating leg lifts.** | On the quadruped perform hypopressive exercise  (after 2 full breaths, remain in apnoea and activate transversus abdominis and pelvic floor and hold for 10 seconds) with flexion of upper limbs and  stretching of the lower limbs (mountain or dog yoga position Inverted) and add one leg lift to the ceiling and hip and knee flexion- extension movement.  Repeat with the other leg. | 10 seconds. |  | |
| **Active stretching and relaxation and body awareness session.** | Stretching arms, legs, neck, lower back, standing, sitting, calmly, paying attention to your breathing. Return to calm. | 5 minutes. |  | |

| **MONTH 4, Days 26, 28, 30, 32** | | | |  |
| --- | --- | --- | --- | --- |
| **Pelvis mobility and relaxation of the CORE and pelvic floor** | Standing, pelvic awareness exercise. Make infinite circles in both directions with the pelvis, hands on hips. | 60 seconds |  | |
| **Ambulation Exercise with Sustained Contractions** | Walking at a comfortable pace while performing fast, sustained contractions, without going apnoeic. | Each person will do a specific number of repetitions according to their assessment. |  | |
| **"Skeeping" with transverse activation and SP awareness** | Lift the knees alternately as if we were running in place, paying special attention to activating the CORE and maintaining awareness of the  pelvic floor. | Each person will do a specific number of repetitions according to their assessment. |  | |
| **Balance and motor control exercises on trunk with** | Initially perform this exercise on a stable base, later on a trunk with an unstable base or on a roller. Bring the ketellball between the legs and push the hips forward and bring the ketellball above the chest, lower the ketellball in a controlled manner. | 10 repetitions. |  | |
| **Isometric squat with ketellball.** | Feet placed wider than hip width apart. Hold ketellball with arms extended in front of you. Deep squat, hold for 3 seconds and raise, all activating transverse and pelvic floor. | 10 repetitions. |  | |
| **Deep squat with ketellball** | Feet placed wider than hip width apart. Pick up ketellball with arms extended in front. Deep squat and pull up, all activating transverse  and pelvic floor. | 10 repetitions. |  | |
| **Alternating stride with** | Standing, catch ketellball with arms extended in front. Lunge forward alternating legs.  Activating transverse and pelvic floor. | 10 repetitions of each leg. |  | |
| **Burpees** | Standing, with feet shoulder width apart and arms at your sides, activate the transverse abdominis by drawing the navel in and up and contracting the pelvic floor muscles at the same time. From the standing position, lower into a squat position and place hands on the floor, jump up and bring feet into plank position, hold plank position and return to start. | 10 repetitions. |  | |
| **Jump into the drawer** | Feet together, jump over a crate and jump down.  It can be done like climbing stairs. Raise awareness of pelvic  floor activation. | Repetitions: 10 |  | |
| **Exhalation crunch with ketellball** | Crunch on exhalation, activating abdominals, head lift with hands  behind the head. | 10 repetitions. |  | |
| **Crunch with side bends.** | Same exercise, but with 2 ketellballs (1 in each hand) or weights, slight flexion of the head, and instead of raising the trunk, do inclines bringing the  ketellball close to each heel. | 10 repetitions. |  | |
| **Crunch with trunk lift and laterality.** | Same position, but place the lateral side of the left foot on the opposite knee.  Lifting the trunk with the ketellball, bring the right shoulder to the left knee. Repeat on the other side. | 10 repetitions. |  | |
| **Oblique with lift and weight.** | Sitting, with the trunk leaning backwards and without supporting the legs, carry the ketellball alternately to the left and right. | 10 repetitions on each side. |  | |
| **Active stretching and relaxation and body awareness session.** | Stretching arms, legs, neck, lower back, standing, sitting, calmly, paying attention to your breathing.  Return to calm. | 5 minutes. |  | |
