# Supplementary material for: Effectiveness of radiofrequency and exercise-based rehabilitation on symptoms associated with pelvic floor dysfunction in breast cancer patients: A study protocol
Source: PLoS One. 2025 Aug 29;20(8):e0330156. doi: 10.1371/journal.pone.0330156 (PMC12396660; doi:10.1371/journal.pone.0330156)
Supplement: S3 File — (DOCX) [file pone.0330156.s003.docx]

**Biomedical Research Ethics Committee Application Memorandum**

**Title**

Effectiveness of radiofrequency and exercise‐based rehabilitation on symptomatology associated with pelvic floor dysfunctions in breast cancer patients. Randomized clinical trial

**Name of IP research project**

Cristina Orts Ruiz

**RESEARCH TEAM:**

Dr. Da. Cristina Salar Andreu

Dr. Sergio Montero Navarro

Dr. Da. Sonia del Río Medina

Dr. D. José M. Botella‐Rico

Dr. María Torres Lacomba

Dr. D. Josep C. Benítez Martínez

Dr. Jesús Sánchez Más

**ABSTRACT**

Breast cancer has a high impact, affecting 2.3 million women worldwide in 2022. In Spain, there were 40,203 new cases in 2023. In addition to the economic and social costs, patients suffer genitourinary dysfunctions due to cancer treatments. Genitourinary Menopausal Syndrome (GUS) affects 50% of menopausal women, with a higher prevalence in women with breast cancer.

This study aims to compare the effectiveness of radiofrequency (RF) and pelvic floor muscle exercise (PFME) in the treatment of pelvic dysfunctions associated with UGS in patients with breast cancer. A randomised, double‐blind clinical trial will be conducted in collaboration with the Asociación de Mujeres Afectadas por el Cáncer de Mama de Elche y Comarca (Association of Women Affected by Breast Cancer of Elche and Region) and the CEU Cardenal Herrera University. Women diagnosed with breast cancer and pelvic dysfunction will participate, divided into three groups: one will receive therapy based on PMSC, one on RF and the other on PMSC+RF.

Both techniques are expected to improve the quality of life of patients, where RF could offer additional benefits due to its proven effectiveness in the treatment of vaginal dryness and dyspareunia. The expected results will contribute to more effective and less invasive treatment protocols. The project presented has the potential to positively impact the health and well‐being of women with breast cancer, reducing the symptoms associated with the disease and its treatment, and improving their quality of life.

Keywords: Breast cancer, genitourinary syndrome, radiofrequency, physical exercise

1. **Introduction**
   1. **Breast cancer: incidence and economic impact.**

Breast cancer is undoubtedly a disease of major global impact, with important health, economic and social implications. Thus, in 2022, nearly 2.3 million women worldwide were diagnosed with breast cancer, of whom 666103 died from the disease^1^. In 2023, 40203 new cases of breast cancer were registered in Spain and 6759 died^2^. These data indicate that, although the survival rate started to improve in the 1990s, thanks to the implementation of early cancer detection programmes and comprehensive treatments including effective drugs in several countries^3^, even more effort is needed to improve early detection and treatment efficiency, resulting in more encouraging data.

It is estimated that breast cancer can cost up to €42,000 in total, including direct and hidden costs, as well as lost income due to sick leave, redundancies and reduced working hours. Specifically, 96% of patients have spent an average of €3,590 on medical expenses such as nutrition services, tests and consultations in private health care, psychology services or rehabilitation^4^. Moreover, breast cancer does not only affect individual women, but also has an impact on the family balance, society and the health care system^5^, so the negative socio‐ economic impact is even greater and beyond real estimation.

- 1. **Aetiology and risk groups**

Breast cancer is a disease characterised by the uncontrolled growth of altered breast cells, which can form tumours.

This type of cancer usually originates in the milk ducts or milk‐producing lobules of the breast. In its early stage, known as stage 0 or in situ, the cancer does not pose a life‐threatening threat. However, as the cancer cells spread into the surrounding breast tissue, they can form nodules or cause thickening^3^. Invasive cancers have the potential to spread to nearby lymph nodes or other organs, known as metastases. These metastases can be fatal if not properly treated.

Some inherited genetic mutations, such as mutations in the BRCA1, BRCA2 and PALB2 genes, significantly increase the risk of breast cancer. Women with these mutations may consider risk‐ reducing strategies such as preventive mastectomy of both breasts^3,6^.

Being a woman is the main risk factor for developing breast cancer. Other factors that increase the likelihood of developing breast cancer include ageing, obesity, excessive alcohol consumption, family history of breast cancer, history of radiation exposure, reproductive history (such as age at menarche and first pregnancy), tobacco use and postmenopausal hormone treatment. Approximately half of breast cancer cases occur in women with no identifiable risk factors other than gender (female) and age (over 40 years)^3,6^. Family history increases the risk, although the majority of women diagnosed do not have a family history, so the absence of a family history does not necessarily imply a lower risk of developing the disease^3^.

- 1. **Symptoms**

Symptoms of breast cancer are often not apparent in its early stages, so detection often occurs through screening programmes, such as routine mammography, or as a result of follow‐up for other conditions. In symptomatic cases, the presence of a breast lump is common, which is usually firm, with irregular borders and not painful. However, other symptoms such as scaling of the areola and skin, crusting, redness, swelling of the breast, dimpling of the skin, breast pain, bone pain, skin ulcers, swollen lymph nodes, weight loss and discharge of blood may also be present^7^.

- 1. **Treatment**

The treatment approach varies according to the individual patient characteristics, the subtype of the disease and its extent whether it is limited to the breast and lymph nodes (stage II or III) or has spread to other parts of the body (stage IV).

In the medical field, a combination of treatments are used to reduce the chances of cancer recurrence, such as:

‐ Surgery to remove the breast tumour.

‐ Radiotherapy to reduce the risk of recurrence in the breast tissues and surrounding regions.

‐ Drug treatments aimed at killing cancer cells and preventing their spread, which may include hormonal therapies, chemotherapy or specific treatments with biologics.

The surgical intervention may consist of the removal of the tissue affected by the cancer (lumpectomy or partial mastectomy) or the complete removal of the breast (total mastectomy). In surgery for invasive cancers, the lymph nodes are removed. In the past, a complete removal of the axillary lymph node bed (called a complete axillary dissection) was considered essential to prevent the spread of cancer. However, nowadays, a less invasive procedure known as "sentinel node biopsy" is preferred, as it involves fewer complications^3,8^.

The choice of drugs to treat breast cancer is based on the specific biological characteristics of the cancer, which are identified by specialised tests, such as the determination of tumour markers. Most of the drugs used to treat cancer are already included in the World Health Organisation's Model List of Essential Medicines ^9^.

Drug treatments (chemotherapy) for breast cancer can be administered before (neoadjuvant) or after (adjuvant) surgery, and are determined by the biological subtyping of the cancer. Cancers expressing oestrogen receptors (ER) or progesterone receptors (PR) often respond favourably to endocrine treatments such as tamoxifen or aromatase inhibitors. These drugs are administered orally for 5‐10 years and significantly reduce the chances of recurrence in hormone receptor‐positive cancers, although they may cause menopausal symptoms^3.^

On the other hand, cancers that do not express ER or PR, called hormone receptor negative, are treated with chemotherapy unless the tumour is very small. Current chemotherapy regimens are highly effective in reducing the chances of cancer spread or recurrence and are usually administered on an outpatient basis. Under normal conditions, chemotherapy for breast cancer does not require hospitalisation^3^.

Some breast cancers can overexpress a molecule called the HER2/neu oncogene independently. This type of cancer can be treated with targeted biologic drugs (immunotherapy), such as trastuzumab, which are highly effective but expensive, as they are antibodies rather than chemicals. These biological treatments are combined with chemotherapy to increase their effectiveness in killing cancer cells^3^.

Radiotherapy is used to treat any remaining microscopic tumours in the breast tissue or lymph nodes to reduce the likelihood of a recurrence that may involve the chest wall^3.^

Radiotherapy plays a key role in the treatment of breast cancer. In early stages, it can prevent the need for a mastectomy. In more advanced stages, even after a mastectomy, it can decrease

the risk of recurrence. In advanced cases, in certain situations, radiotherapy can reduce the likelihood of mortality due to the disease^3^.

- 1. **Genitourinary syndrome in breast cancer**

Women diagnosed with breast cancer experience significant emotional distress due to physical, psychological and social sequelae, including mastectomy, lymphoedema, early menopause, infertility, fear of recurrence, emotional stress and changes in family and work relationships^10^.

Side effects of these treatments include dysfunction in women's sex lives^11^, infertility, anovulation, amenorrhoea, vaginal atrophy, hot flushes and early menopause^12^. The symptoms of menopause affect various areas of women's lives, diminishing their quality of life^13^.

Genitourinary syndrome of menopause (GUS) is common in 50% of menopausal women, but its incidence is higher in women with breast cancer, mainly due to surgery, chemotherapy, radiation and hormone therapy^14^. SGU is a set of symptoms and signs caused mainly by oestrogen depletion and leads to changes in the vagina and external genitalia as well as in the urethra and bladder^15^. UGS is characterised by vaginal dryness, vulvar and vaginal burning and irritation, lack of lubrication, dyspareunia, dysuria, urinary urgency and recurrent urinary tract infections^14^, as well as pelvic floor and intestinal tract dysfunctions such as constipation and diarrhoea^16^.

Sousa *et al.*^17^ noted that 59% of women with breast cancer surveyed in their study experienced genitourinary problems, with stress urinary incontinence (SUI) and voiding urgency being the most common. In addition, many reported increased urinary frequency, nocturia, recurrent urinary tract infections, prolapse, burning, discomfort/pain, overactive bladder and dyspareunia, worsening with adjuvant endocrine therapy^17^.

In another study, Cordoba de Juan *et al*.^18^ found that 40.2% of women with breast cancer surveyed had symptoms of pelvic floor dysfunction. However, other authors indicate lower rates of pelvic floor dysfunction in female breast cancer survivors compared to a control group, despite the fact that these women have undergone a greater number of pelvic floor‐related surgical interventions due to breast cancer^19^.

- 1. **Impact on pelvic function**

Urinary incontinence (UI) is the most common genitourinary dysfunction among women with breast cancer, followed by faecal incontinence (FI) and, lastly, prolapse^20^. Both Stahlschmidt *et al*.^21^ and Cordoba‐de Juan *et al*.^18^ found that 40% to 47% of the women surveyed had UI, while 7.3% had urge urinary incontinence (UUI), 25% had SUI and 25% had overactive bladder (OAB). No significant differences were found between women receiving aromatase inhibitors and those taking tamoxifen. In addition, they concluded that older patients with lower adherence to treatment had a higher incidence of UI^21^.

On the other hand, after five years of tamoxifen treatment, 93.33% of the women surveyed had UI, 21.43% of whom had UI, 71.43% had SUI and 7.14% had mixed UI (MUI), with a frequency four times higher in women who had had children^22^. However, due to the sample size, an association with age and adjuvant therapies could not be established. This percentage dropped to 38% after 10 years from breast cancer diagnosis, with no difference between those taking aromatase inhibitors and those who did not^(20).^ Therefore, Robinson *et al*.^20^ could not confirm that the higher testosterone levels produced by these drugs protect against UI. As for FI, one study found no FI among women with breast cancer, but 6.1% gas incontinence^18^. However, the percentage of women with FI 10 years after diagnosis was 17.6%, being more common in those

taking aromatase inhibitors (29.8%) compared to those who did not (16.4%)^(20).^ In addition, age was found to be associated with FI, but no direct association was found between tamoxifen, body mass index or pelvic surgery with FI.

Some authors reported that between 7.3% and 8.1% of the women surveyed had prolapse, with no significant differences between those who took aromatase inhibitors and those who did not^18,20^. Therefore, there is still a need for further studies to generate knowledge in order to understand the association between the type of treatment received and pelvic dysfunction in patients diagnosed with breast cancer.

- 1. **Impact on sexual function**

According to Oberguggenberger *et al*.^23^, 68.8% of women with breast cancer experienced problems in sexual desire or interest, compared to 58.8% of the control group. In addition, sexual desire was the most affected aspect according to the Female Sexual Function Index (FSFI) questionnaire, both immediately after treatment and six months later, mainly in women older than 55 years, postmenopausal, married and with children.

Significant differences in sexual desire were found to be related to the type of surgery (radical mastectomy, skin sparing and nipple sparing), the method of removal (axillary lymphadenectomy or sentinel node) and the woman's menopausal status. Specifically, in women who underwent mastectomy, decreased sexual desire increased at both six and twelve months after diagnosis^23^.

In a study by Robinson *et al*.^20^ of women diagnosed with breast cancer, ten years after diagnosis, no differences were found in hypoactive sexual desire between sexually inactive women who were taking tamoxifen at the time and those who were not. However, there were variations depending on aromatase inhibitor use, with lower sexual desire in those taking aromatase inhibitors.

On the other hand, 17.3% of the women surveyed expressed lack of interest in having sex, mainly due to menopausal symptoms such as vaginal dryness and feelings of dissatisfaction with their body in the last four weeks. However, more sexual problems were observed after treatment (72.09%) compared to the time of diagnosis (51.35%) and twelve months after diagnosis (59.26%).

According to the study by Oberguggenberger *et al*.^23^, sexual interest and desire were positively correlated with partner satisfaction and different dimensions of quality of life, while they were negatively related to depression.

More than 50% of women with breast cancer experienced discomfort during intercourse, which was associated with vaginal dryness common in this group. In addition, time was found to be a significant factor in dyspareunia, with women reporting greater pain just after treatment (41.864%) and six months later (40.74%) compared to the time of diagnosis (32.43%). This was attributed as a consequence of chemotherapy^18^.

Women diagnosed with breast cancer experienced more discomfort during sex compared to women without breast cancer, and the researchers suggested that this may be related to self‐ reported femininity issues^23^. Another study by Robinson *et al*.^20^ examined how taking aromatase inhibitors affected this variable. Ten years after diagnosis, women taking aromatase inhibitors were found to have greater sexual distress (70%) compared to women not taking aromatase

inhibitors (52.7%). The authors associated this finding with vulvovaginal atrophy secondary to this treatment^20^.

- 1. **Treatment for the SGU**

There are different treatment options for the symptomatology associated with UGS, ranging from conservative approaches to more invasive options such as surgery, depending on the severity of the symptoms to be treated.

Women who have survived breast cancer experience more intense symptoms of UGS compared to those who are postmenopausal and have not faced the disease.

Although hormone therapy is widely recognised as the most widely supported treatment for UGS, there are restrictions and challenges associated with its use. Estrogen is contraindicated in individuals with a history of breast cancer, endometrial cancer, deep vein thrombosis, pulmonary embolism, liver disease and unexplained vaginal bleeding. Therefore, hormone therapy, whether systemic or local, cannot be used because oestrogen therapy combined with progesterone increases the risk of stroke, pulmonary embolism and deep vein thrombosis, but not coronary heart disease^24^.

- - 1. **Non‐conservative treatment**

Among the most invasive options to address UGS is surgery to correct SUI. This intervention aims to strengthen the pubourethral ligaments and paraurethral connective tissue in the mid‐ urethra^25^.

Surgical treatment is generally categorised into abdominal procedures (open or laparoscopic), vaginal procedures and urethral bulking agents. Abdominal procedures include the Marshall Marchetti Krantz (MMK) technique, Burch colposuspension and the pubovaginal sling^25,26^. Vaginal procedures, on the other hand, include the modified Pereyra procedure (PPM) and tension‐free suburethral bands (TBS), which can be either retropubic (TVT) or transobturator (TOT)^26,27.^ BLTs, usually made of polypropylene, are placed vaginally to pass the mesh through the obturator foramen and under the midurethra with as little damage to surrounding tissues as possible. In addition, the technique of single incision bands, fixed to the obturator muscle or its fascia through a small vaginal incision, was introduced, associated with less pain, but with similar adverse effects compared to conventional BLTs^25^.

The urethral volume augmentation technique involves the injection of synthetic materials, such as collagen, into the urethral mucosal layer to provide support and narrow the opening of the bladder neck. This procedure is performed in an office setting under local anaesthesia. Two to three injections are often required to achieve significant improvement in symptoms^28^.

The most common complications following surgical treatment are voiding difficulty, urinary tract infection, postoperative dyspareunia, mesh erosion, persistent or recurrent UI or pelvic organ prolapse, genitourinary tract injury (including bladder perforation) and gastrointestinal tract injury^29,30^. Short‐term complications associated with the TVT technique have been reported to be as high as 19% and include urinary retention, wound infection and bladder laceration during surgery. Other possible complications of TVT sling surgery include iatrogenic perforation of the lower urinary tract or bowel, significant bleeding, mesh exposure, erosion (of both the urethra and bladder), implant infections, occurrence of voiding dysfunction (such as urinary urgency or bladder outlet obstruction), retention and urinary tract infections. While many of these complications are short‐term and can be resolved, others, such as erosion and organ

perforation, can be more difficult to treat and often require additional surgeries with increased risk of complications^31.^

For menopausal symptoms after breast cancer, regardless of the type of breast cancer (hormonal, Her2 or triple negative), systemic hormone treatment remains contraindicated. This is valid for oestrogen, progesterone, dehydroepiandrosterone (DHEA) but also for testosterone treatments that are regularly tested to improve sexual function in women in the USA. However, it is not yet approved due to limited efficacy and lack of long‐term safety data. Lifestyle optimisation and regular physical exercise are the most effective therapies for alleviating all menopausal symptoms^32^.

Oral ospemifene is approved for use in the treatment of dyspareunia due to menopause. Once‐ daily oral dosing improves vulvovaginal symptoms similar to local oestrogen therapy, but is not approved for use in breast cancer and may increase the risks of venous thromboembolism^33^.

Among the approved pharmacological options is oxybutynin which is an anticholinergic that blocks the muscarinic receptor in the smooth muscle of the bladder and inhibits detrusor contractions. Anticholinergics often cause dry mouth and constipation as predominant side effects. In addition, there is a possibility that may aggravate pre‐existing cardiac arrhythmias and worsen narrow‐angle glaucoma^34^. In non‐hormonal treatment, selective serotonin reuptake inhibitors (SSRIs), serotonin and norepinephrine reuptake inhibitors (SNRIs) and gabapentin can be used to treat vasomotor symptoms of menopause. SSRIs and SNRIs, such as paroxetine, escitalopram and venlafaxine, are antidepressants that treat vasomotor symptoms. Paroxetine, in particular, is the only drug approved by the FDA (US Food and Drug Administration) for this indication and symptoms decrease one week after starting treatment. However, paroxetine and fluoxetine should not be used concomitantly with tamoxifen due to inhibitory effects on the CYP2D6 enzyme^35,36^.

- - 1. **Conservative treatment**

The conservative approach encompasses various lifestyle interventions, normalisation of the pelvic floor musculature, as well as other strategies such as bladder re‐education, electro‐ stimulation, radiofrequency or the use of moisturising and lubricating gels. Lifestyle modifications and regular physical exercise have been suggested as the most effective therapies to mitigate all menopausal symptoms. These treatments are mostly non‐invasive, with little or no side effects and do not hinder possible future treatments if needed. Furthermore, their cost is usually affordable, in any case, much lower than the cost of pharmacological or surgical treatment. For all these reasons, conservative treatment should be considered the first line of management of UGS

Physiotherapy is fundamental in the treatment of pelvic floor dysfunctions. It involves a wide range of therapeutic techniques including the implementation of therapeutic physical exercise and joint mobilisation techniques to improve muscular and respiratory fitness

Within the therapeutic exercise approach is CORE normalisation and pelvic floor muscle training (PFMT). CORE stabilisation is an important approach in physiotherapy, suggesting that lumbopelvic dysfunction may be caused by an imbalance between the larger supporting structures and the smaller, weaker ones, such as the pelvic floor, transverse abdominis and respiratory diaphragm. The aim of this approach is to restore balance and strength to the core region of the body. Abnormal pelvic postures can lead to increased stress on the joints and excessive muscle activation. Therefore, incorrect posture can lead to trigger points, hypertonicity and pelvic pain. It is essential that physiotherapy design a comprehensive

treatment plan that addresses these musculoskeletal irregularities. This plan should include muscle strengthening exercises, stretching, postural techniques, treatment of abdominal diastasis and pelvic floor re‐education. PFMT involves the active renewal of neuromuscular control in the muscles of this area. This process can involve strengthening weakened muscles, decreasing muscle tension and re‐coordinating overall muscle control^37^

The mechanisms by which therapeutic exercise and PFMT exert their beneficial effects in breast cancer patients are not yet fully understood. However, it is postulated that these interventions may act through several pathways, including strengthening pelvic floor musculature, improving proprioception, decreasing abdomino‐pelvic symptomatology and improving quality of life^38‐40^.

Within their scope of competence, the physiotherapist may work directly with the pelvic floor musculature, providing education and training to improve motor control in this area. The physiotherapist will employ techniques that promote active relaxation and contraction of the pelvic floor. This learned response to relax and contract the pelvic floor can be cultivated during physical therapy through verbal cues, manual therapy and biofeedback^3(7).^

A 2019 study by R. Schvartzman *et al*.^41^ explored the hypothesis that pelvic floor physiotherapy could improve pain, sexual function and quality of life in climacteric women suffering from dyspareunia. The findings of this study help to determine the crucial role of physiotherapy in these conditions. According to the cited article, it was concluded that physiotherapy intervention in women with climacteric dyspareunia resulted in significant improvements in pain, quality of life, sexual function and pelvic floor musculature^41^.

Pelvic floor rehabilitation in postmenopausal women with UI improves symptoms and signs of pelvic dysfunction associated with UGS, as well as showing a positive impact on activities of daily living, quality of life and sexual function^42‐44^.

Radiofrequency (RF) is another conservative technique that has gained recognition due to its practicality and feasibility as a safe and effective therapy for treating vaginal symptoms and sexual dysfunction. This technique uses high‐frequency electromagnetic waves to generate thermal micro‐points in the superficial and deep dermis, with limited thermal action in terms of laterality and depth. This approach allows adequate regeneration without scarring or sequelae, and stimulates tissue repair by promoting the production and reorganisation of collagen, elastic fibres and vascularisation, thus improving trophism. In gynaecology, it is used therapeutically to improve function in several areas, including the vaginal walls, vulvar vestibule, urethral meatus, labia minora, labia majora, perineum and perianal region. The first pilot study on vaginal RF (Linly™ Loktal Medical Electronics) investigated the clinical response of 14 patients with SGU symptoms after undergoing RF to the vagina and vaginal introitus. "RF proved to be effective in the treatment of vaginal dryness and dyspareunia, eliminating the need for vaginal lubricant during the observation period and improving vaginal tropism”^45^. A subsequent study examined the effect of RF on vaginal health, microbiota and cellularity in postmenopausal women, demonstrating that RF is effective and safe in restoring vaginal balance, promoting the predominance of Lactobacillus species and an acidic pH in the vaginal fluid, which may protect these women from vaginal infections, inflammation and urogenital tract infections^46.^

A randomised clinical trial evaluated the effect of RF for SUI compared to PFMT and the combination of both therapies (RF + PFMT) in 117 climacteric women with SUI. After treatment, a significant improvement in urinary scores was observed in all three groups, with a greater benefit in the RF + PFMT group. Vaginal symptoms and vaginal laxity improved with RF and the vaginal health index was higher in the RF and RF + PFMT groups. In addition, sexual function

improved in the RF and PFMT groups. The combination of RF and PFMT demonstrated significant improvement in SUI symptoms and vaginal dryness, while vaginal laxity improved similarly. However, the combination of RF and PFMT on sexual function did not show benefits superior to those achieved with the individual therapies^47^.

Another study showed that RF, in addition to being an effective and safe treatment for vulvar lichen sclerosus, causes a significant increase in the vulvar concentration of type III collagen relative to type I collagen after treatment, which was associated with a marked improvement in symptoms and elasticity^48^. In 2010, Millheiser *et al*.^49^ demonstrated its efficacy in the treatment of postpartum vaginal laxity syndrome. Evidence suggests usefulness and efficacy in the treatment of atrophic vaginitis, orgasmic dysfunction and SUI^49,50^.

Currently no studies have evaluated the effectiveness of RF as a treatment for the symptoms associated with pelvic dysfunction associated with the characteristic UBS in breast cancer patients. Due to the proven safety and efficacy of RF as a conservative therapy for pelvic dysfunction, it is necessary to evaluate its therapeutic action in patients with breast cancer, and compare it with the reference conservative treatment such as PMSCS, to see if it could be a coadjuvant treatment.

Hypothesis

Conservative RF‐based treatment should be effective in improving symptoms associated with the onset of UGS and impaired pelvic function in women diagnosed with breast cancer, and may be an adjuvant therapy to PMSCS.

1. **Objectives**

The aim of the study is to compare the effectiveness of RF‐based treatment with PMSCS‐based treatment on UGS‐related pelvic dysfunction in breast cancer patients.

Specific objectives

To evaluate the effect of RF, PMSC and the combination of both therapies on UI, FI and prolapse in women diagnosed with breast cancer.

To evaluate the effect of RF therapy and PMSCS on the quality of life of women diagnosed with breast cancer.

To analyse the effect of RF and PMSCS on sexual function in women diagnosed with breast cancer.

To find out the impact of RF therapy and PMSCS on the perception of body self‐image in women diagnosed with breast cancer.

1. **Methodology and work plan**
   1. **Study design**

A randomised, double‐blind clinical trial will be conducted among women diagnosed with breast cancer with pelvic dysfunction who will undergo therapy based on PMSCS and RF, alone or in combination. The study will be carried out at the facilities of the Asociación de Mujeres Afectadas por el Cáncer de Mama de Elche y Comarca (AMACMEC), with registered office at Calle Olegario Domarco Seller, 93. Entresuelo de Elche (Alicante), and holder of tax identification number G53270146. This association, which currently has a total of 812 members, currently has a collaboration agreement signed with the CEU Cardenal Herrera University and has signed the authorisation to carry out this specific study. The study will start in September 2024, with an estimated duration of two years as detailed in a timeline below. AMACMEC has a physiotherapy room that ensures privacy and suitability for conducting RF assessments and treatment, as well as a physical exercise room in which the PMSC‐based groups will be conducted.

The study will be carried out in accordance with the Declaration of Helsinki and in accordance with current Spanish legislation (Royal Decree 223/2004 and the Biomedical Research Act of 2007). Blinding of the results recorded will be guaranteed, as no personal data allowing the identification of the subject or data referring to email accounts will be collected. The data will be stored in the OneDrive cloud associated with the FUNDACIÓN UNIVERSITARIA SAN PABLO CEU (hereinafter, FUSP‐CEU), to which access will only be granted to the researchers responsible for the analysis, extraction and handling of the data, by means of an access code (Jesús Sánchez Más and Cristina Orts). The data will be identified by a code, so that it does not include information that could identify the patient. The data will be processed in accordance with the provisions of Regulation (EU) 2016/679 of the European Parliament and of the Council of 27 April 2016 on the protection of individuals with regard to the processing of personal data and on the free movement of such data and repealing Directive 95/46/EC. The data will be processed under the responsibility of the FUSP‐CEU, for the purpose of managing your participation in this research project. You may contact the FUSP‐CEU Data Protection Officer by sending your request in writing to the postal address C/ Tutor nº 35 ‐ 28008 Madrid or to the e‐mail address [dpd@ceu.es.](mailto:dpd@ceu.es) Approval will be requested from the Ethics Committee for Biomedical Research of the CEU Cardenal Herrera University. Once approval has been obtained from the Committee, the study will be registered on clinicaltrials.gov

- 1. **Participants**

Women diagnosed with breast cancer from AMACMEC will be invited to participate voluntarily in the study. In order to recruit participants, informative group talks will be held. Subsequently, information about the project will be expanded through a personal interview to be conducted on the day of the participant's assessment for inclusion in the study.

Inclusion criteria are: women of legal age with a clinical history of breast cancer, who agree to participate in the study and who present pelvic dysfunction as assessed by the *Pelvic Floor Distress Inventory*^51^ (PFDI20) questionnaire ≥ 100, criterion established by previous results obtained by

the research group that characterised the pelvic floor‐related symptomatology in 250 women diagnosed with breast cancer and associated with AMACMEC (project "Abdomino‐ pelvic dysfunctions in women affected by breast cancer, IDOC22‐07, unpublished data).

Exclusion criteria are: having performed PMSCT or received RF in the last 12 months, use of vaginal oestrogens in the last 6 months, systemic hormone therapy in the last 6 months, laser therapy in the last 6 months, absence of pelvic floor contraction according to the Modified Oxford Scale, pacemaker use, decompensated heart or metabolic diseases, cognitive deficits, peripheral or central neurological disorders, previous surgeries in the pelvic region, skin pathologies or wounds in the treatment area or presence of an active urinary tract and/or vaginal infection.

- 1. **Sample calculation**

The sample size was calculated according to previous studies demonstrating statistically significant difference in pelvic floor symptomatology and pelvic floor‐related quality of life after RF treatment^52^. Considering a 30% loss to follow‐up in the sample, with an alpha risk (α) of 5% and a power (1‐β) of 80%, a sample size of 117 women (39 in each group) is calculated.

- 1. **Study design**

The first contact with the patient who is likely to participate in the project will always be made by the principal investigator (PI), who will duly inform them of the study and give them the information sheet for participants (ANNEX 1) and the informed consent form (APPENDIX 2). Once they have accepted to participate in the study, they will be given the PFDI20 questionnaire that will allow them to know the patient's pelvic dysfunction and their suitability to be included in the study. They will then be provided with an *ad hoc* questionnaire (ANNEX 3) to ascertain socio‐demographic characteristics, as well as information related to the diagnosis and treatment of breast cancer, as well as other specific validated questionnaires to assess pelvic function, vaginal symptoms, sexual function and perception of body image, which will be detailed later on.

Then, always performed by a physiotherapist specialised in the pelvic floor area, the strength of the MSP in women will be measured.

Another investigator blinded to the above determinations will perform randomisation to the intervention groups. Participants will be assigned to one of three intervention groups: fractionated non‐ablative RF therapy, PMSCS therapy and combined RF and PMSC (RF + PMSC) therapies. Randomisation will be performed using SAS 9.4 software (SAS Institute, Cary, NC, USA) with a 1:1:1 allocation. The information on the treatment to be used will be kept inside a sealed opaque envelope identified by a number.

One physiotherapist will perform the RF‐based intervention, another physiotherapist will perform the PMSE‐based intervention. Another physiotherapist, who will not know the intervention group, will perform the post‐treatment determinations. It will not be possible to blind participants to the treatment

- 1. **Radiofrequency‐based treatment**

The proposed therapeutic process will consist of applying RF in the modality of capacitive electrical transfer (Capernergy® device model C500 Urogyne). This is a device designed mainly for addressing dysfunctions in the urogynaecological area, where the increase in tissue temperature is regulated by a temperature sensor, with 3 frequencies (0.8MHz, 1MHz and 1.2 MHz) that will allow different tissue depths to be addressed, and a power of 310w. This device consists of two electrodes: an active capacitive electrode to be placed in the vaginal area with a probe cover and water‐soluble gel and another dispersive electrode or return plate to be positioned in the lumbosacral region.

The protocol to be performed is based on the treatment described by Pinheiro *et al*.^53^ for the treatment of postmenopausal SGU, in which the participants will be placed in a lithotomic position. The treatment temperature will be set at 41ºC, with a frequency of 1Mhz and power of 75KJ. Once the indicated temperature has been reached, the physiotherapist will perform semicircular movements on the vaginal wall for 2 minutes on the anterior vaginal side and for 4 minutes on the posterior vaginal wall. A total of 5 sessions will be carried out with an interval of 7 days between each one.

- 1. **EMS‐based treatment**

The EMSP will consist of a targeted programme, with a protocol of CORE and pelvic floor exercises established based on the assessment of the patients' strength, endurance and fatigue, with a frequency of twice a week led by a physiotherapist and a duration of 45 minutes each day and a period of 16 weeks. It will be carried out in groups of 8 people and accompanied by a physiotherapist experienced in therapeutic exercise.

Each session will consist of exercises to activate the pelvic floor muscles in isolation and in association with the CORE muscles, both static and dynamic.

The design of the exercise protocol and sequences is based on an adaptation of the programme described in a previous study carried out in 117 climacteric women with pelvic dysfunction, which showed significant improvement in UI symptoms, vaginal symptoms and sexual function similar to what we intend to analyse in this project^47^, and on previous studies on therapeutic exercise in the prevention and treatment of pelvic floor pathologies based on EMSP, hypopressive technique, CORE work and the use of unstable bases^54‐63.^

The PMSC protocol will be expanded by monthly targets and will have two different sessions per week, to encourage adherence and motivation based on the variability of the exercises. A detailed exercise programme is attached as ANNEX 4.

- 1. **Determinations**

The determinations indicated in this section shall be made before starting the intervention, 1 week after the end of the intervention, 6 months after the end of the intervention and 1 year after the end of the intervention.

- 1. **Assessment of the strength of the MSP**

Prior to the measurement, participants will be asked to go to the toilet to urinate, thus allowing the bladder volume to be standardised as far as possible^64^. Once the women are in the place where they are to be measured, they will be allowed a resting period of 3 minutes in a sitting position, as this time corresponds to twice the duration of sympathetic system deactivation^65^.

For the force recording, women shall be placed in the gynaecological or lithotomic position, with the genital region and legs unclothed, covered by a sheet. They will then be instructed to remain relaxed^66^.

Assessment of MSP strength and function appears to be best determined by a combination of observation, palpation and intravaginal pressure. Therefore, in this study MSP strength will be assessed both bidigitally with the Oxford scale and via pressure probe.

The first examination will consist of a bidigital palpation to estimate MSP strength during maximal contraction using the Oxford scale based on previous studies indicating the influence of strength, endurance and fatigability on pelvic floor competence and its relationship to the synergistic musculature. The following will be requested:

^‐^ MSP FUNCTION: best of 3 attempts graded as 0= no contraction, 1= partial contraction, 2= contraction of the SP+ contraction with related muscles, 3= isolated contraction of the SP^67^

‐ MUSCLE STRENGTH: is defined as the maximum force that the muscle can generate and is described as the maximum force that a muscle can exert^68^, being the best of the 3 attempts, assessed with the modified OXFORD scale^69^. ORDER: "contract the musculature as hard as possible".

‐ STATIC MUSCLE ENDURANCE: understood as the capacity to maintain an optimal (isometric) contraction60 for as long as possible, measuring the seconds until fatigue is reached^70^. ORDER: "maintain the contraction", being the average of the three assessments.

‐ FATIGILITY OR DYNAMIC ENDURANCE: maximum number of contractions in a unit of time, taking 10 seconds as the average. ORDER: "contract as many times as possible and at the highest speed", the measure being the average of the three assessments^70^.

The Oxford scale is used to assess the contractile capacity of the pelvic floor muscles. It is scored from 0 to 5, as follows: no contraction is 0, very weak contraction is 1, weak contraction is 2, moderate contraction/with tension/and maintained is 3, good contraction and maintaining tension with resistance is 4, and strong contraction and maintaining tension against a resistant force is 5^71^ .

All data will be recorded on an MSP force record sheet (ANNEX 5).

In order to complement the tactile and pelvic measurement based on previous reviews^72^, we will use the inflatable vaginal probe connected to a Phenix manometer, which connected to a screen will allow us to assess the concepts of strength and resistance evaluated.

For this purpose, the therapeutic neuromuscular stimulation and manometry device of the PHENIX series (model: PHENIX LIBERTY; manufacturer: ELECTRONIC CONCEPT LIGNON INNOVATION, Montpeilier, France) will be used. For this manometry, the air probe, connected

to the Phenix biofeedback system, covered by a latex probe cover lubricated with gel, will be used. In the procedure, the labia majora are opened with one hand and slowly rotated into the vagina by holding the back of the manometric probe with the other hand. Basal tone in g will be measured prior to MSP force measurements. The manometry probe will extract pelvic floor pressure signals by measuring both your basal tone and the maximum pressure held for 10 seconds in three measurements, the average of the three being calculated at the command, "contract as hard as you can for as long as possible".

- 1. **Pelvic function and quality of life**

To assess pelvic function and the impact of pelvic dysfunction on quality of life, the following questionnaires will be provided (ANNEX 6):

‐ The PFIQ‐20 (Pelvic Floor Impact Questionnaire Short Form) is used to assess the impact of urinary symptoms, colo‐rectal‐anal symptoms and genital prolapse symptoms^51^. This questionnaire will also be used as an inclusion criterion, to homogenise the characteristics of the population in terms of pelvic dysfunction prior to the intervention, as indicated above.

‐ The ICIQ‐SF (International Consultation on Incontinence Questionnaire ‐ Short Form) is a four‐ question self‐administered questionnaire that identifies individuals with urinary incontinence by assessing frequency, severity and impact on quality of life. It consists of five questions assessing frequency, severity and impact of UI, plus a set of eight self‐diagnostic items related to UI situations experienced by patients. The maximum sum of the response values indicates a score of 21 points, referring to the high impact of UI on an individual's life^73^.

‐ Sandvik severity test. It provides information on the severity of UI by means of two questions. The interpretation based on the score is classified as: 1‐2 mild UI, 3‐6 moderate UI, 8‐9 severe UI, 12 very severe UI^74^.

- 1. **Vaginal symptoms**

The following questionnaire will be provided to assess vaginal symptoms (ANNEX 7):

‐ The vaginal health index (VHI), which consists of a graduated scale of 1 to 5 for each item (vaginal elasticity, fluid volume, pH, epithelial integrity and moisture), is assessed by physical examination^53^. Vaginal elasticity ranges from 1 (no elasticity) to 5 (excellent elasticity), assessed by distension of the mucosa on palpation and placement of the speculum. The volume of fluid, assessed during inspection, varies between 1 (no secretion) and 5 (normal secretion) (white flocculent). The integrity of the epithelium varies between 1 (petechiae already detected on inspection) and 5 (non‐friable tissue and normal mucosa). Moisture ranges from 1 (no moisture detected on inspection and presence of inflamed mucosa) to 5 (normal moisture). The pH will be quantified using a pH indicator strip between 0 and 14 (MColorpHast™‐PH indicator strips) to be placed directly on the right lateral vaginal wall for one minute, giving 1 point for pH 6.1, 2 for pH 5.6‐6.0, 3 for pH 5.1‐5.5, 4 for pH 4.7‐5.0 and 5 for pH ≤ 4.6. The sum of all items represents the vaginal health score, where 25 represents the best vaginal health^76^.

- 1. **Sexual function and self‐esteem**

The following questionnaires will be provided to assess sexual function and self‐esteem (ANNEX 8):

‐ The FSFI (Female Sexual Function Index) questionnaire assesses sexual function over the past 4 weeks. A cut‐off point ≤ 26.5 is considered sexual dysfunction and an increase in the score is considered an improvement^53^.

‐ Dyspareunia assessed using the Visual Analogue Scale (VAS), which allows the intensity of pain described by the patient to be measured with maximum reproducibility between observers. It consists of a 10‐centimetre horizontal line, at the ends of which are the extreme expressions of a symptom. At the left end is the absence or lowest intensity and at the right end the highest intensity. The patient is asked to mark on the line the point that indicates the intensity of the pain during sexual intercourse and it is measured with a millimetre ruler. The intensity is expressed in centimetres or millimetres. The rating will be: 1 Mild pain if the patient scores the pain as less than 3; 2 Moderate pain if the rating is between 4 and 7; 3 Severe pain if the rating is equal to or greater than 8.

‐ Body Image Scale (S‐BIS). It consists of 10 items that assess various dimensions of body image in cancer patients, evaluating: affective, behavioural and cognitive. The items are scored on a four‐point scale (0: not at all; 1: a little; 2: quite a lot; 3: a lot) with a maximum possible score of 3 points. The higher the score, the higher the body image problem. Its brevity facilitates rapid assessment in both clinical and research settings. It shows a stable factor structure between samples, and good psychometric properties with high reliability (Cronbach's alpha=0.93), suggesting that it is a suitable and useful measure for assessing body image in breast cancer patients.

‐ At the end of the intervention, a five‐point Likert scale questionnaire will be administered to determine satisfaction with the treatment, which will be classified as follows: 1 (very dissatisfied), 2 (dissatisfied), 3 (no change), 4 (satisfied), 5 (very satisfied). Women who miss any of the RF sessions and/or whose attendance to the face‐to‐face physiotherapy sessions (RF and/or PMSS) does not reach 80% will be considered as not complying with the study protocol and their participation will be terminated, although they will be included in the analysis (by intention to treat).

- 1. **Statistical analysis**

The Kolmogorov‐Smirnov test will be used to assess the normality of the sample. Comparative analyses between groups will be performed by analysis of variance (ANOVA) or Kruskal‐Wallis test. Associations of categorical variables will be analysed using the Chi‐square test and Fisher's exact test. Intra‐group assessment will be performed using Wilcoxon's test or Student's t‐test for paired samples for continuous variables, and McNemar's test or Bowker's symmetry test for categorical variables. The data will also be evaluated using ANOVA for repeated measures in order to simultaneously verify the influence of the three study groups (between‐group effect), the two assessments (within‐group effect) and to estimate the group × time interaction effect for each of the variables. The results will be analysed by intention‐to‐treat. The significance level will be 5%. SPSS v.29 will be used. The statistical analysis of the data collected will be carried out by a researcher (Jesús Sánchez) blinded for the intervention and for the data collection.

- 1. **Material used**

The registered research group (GIR) at the UCH CEU University with the title Active Physiotherapy, Manual Therapy and Image Analysis (FAMI) has the appropriate equipment and staff to ensure the feasibility of the project. The facilities of the AMACMEC association will be used for the inclusion of the participants, implementation of the intervention and data collection.

With regard to equipment, the following is available in connection with the project:

‐ Radiofrequency equipment: Capernergy® device model C500 Urogyne

‐ Biofeedback equipment: PHENIX (model: PHENIX LIBERTY; manufacturer: ELECTRONIC CONCEPT LIGNON INNOVATION, Montpeilier, France).

- 1. **Timeline**

**Table 1.** Estimated timetable for a duration of two years, depending on the starting month from approval by the Committee.


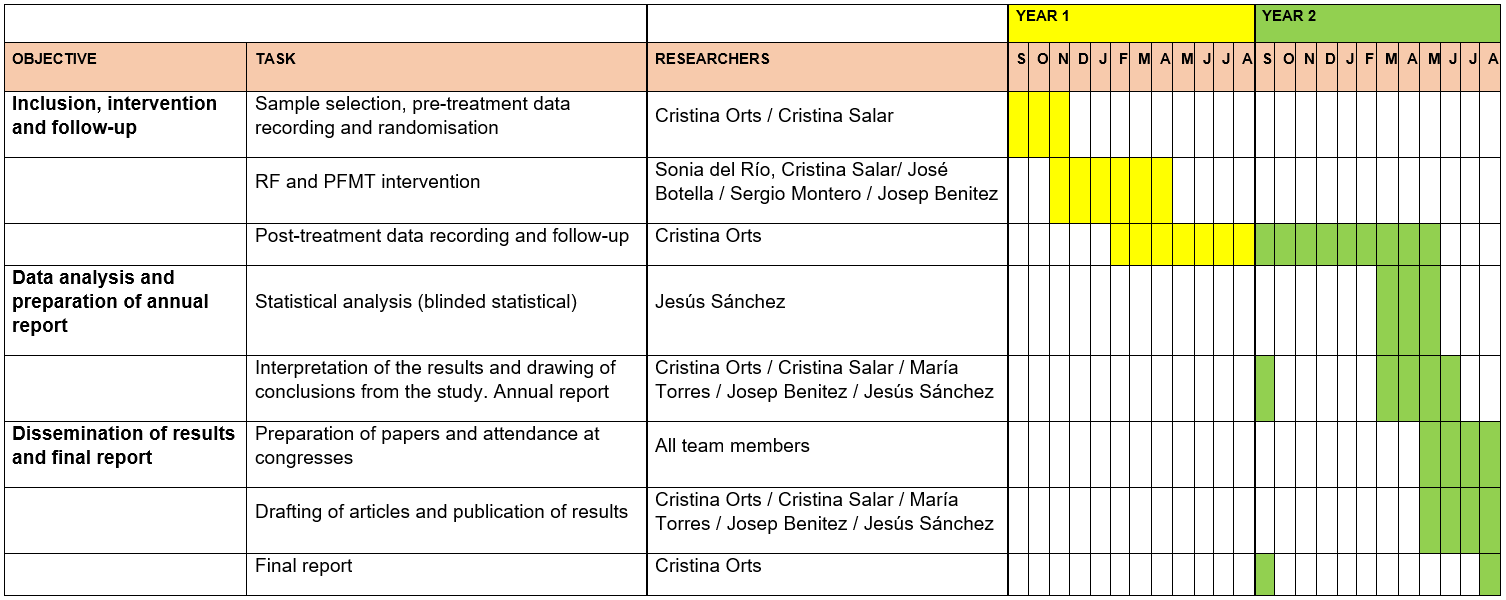


1. **Expected impact of the results**
   1. **Scientific and technical impact**

The scientific‐technical impact of this project lies in the fact that it will provide scientific evidence of the effect of conservative and non‐invasive treatments such as radiofrequency and therapeutic physical exercise in oncological patients, making it possible to reduce the impact of both the oncological process and the effects of the pharmacological and surgical treatments proposed on the quality of life of this population.

All of this will contribute to the availability of greater scientific evidence of physiotherapy in the field of oncology, making it possible to develop new lines of research into both radiofrequency and therapeutic physical exercise in patients with other types of oncological processes, as well as in patients with genitourinary syndrome derived both from the menopause and from the use of other types of treatments and/or interventions that derive from it.

With the conclusions obtained from this study, it will be possible to develop treatment protocols for the prevention and management of pelvic floor dysfunctions in oncology patients, as well as to improve the training of health professionals in the field of physiotherapy in the treatment of oncology patients.

In 2020, the Europe Region World Physiotherapy (ER‐WCPT) pointed out the importance of specialisation in physiotherapy in response to the needs of both professionals and society. For this reason, in recent years the ER‐WCPT has developed a specialisation process for physiotherapists and this study aims to contribute to the specialisation of physiotherapy by applying physiotherapy techniques in the field of oncology.

**4.2. Social and economic impact**

This study will make it possible to develop health promotion campaigns, raising awareness both in society and among health professionals of the need for women diagnosed with breast cancer to prevent the appearance of pelvic floor dysfunctions that develop in them, mainly as a result of the surgical and pharmacological treatments to which they are subjected.

In general, there is a lack of knowledge among health professionals about the relationship between pelvic floor dysfunctions and cancer treatments. This situation leads to a reduction in the quality of life of these women, so the results of this study aim to provide scientific evidence so that other health professionals are aware of the effects that both radiofrequency and therapeutic physical exercise produce in women with breast cancer, which may contribute not only to a better functionality of the pelvic floor but also to a possible improvement in the self‐image of these patients, who often suffer a significant detriment as a result of the treatments received.

Likewise, the expected results of this study would allow a reduction in the costs of medical and psychological care for these patients, which would translate into significant savings for the health system, as well as for the patients' own economy. Associated with this, the number of sick leaves would be reduced, contributing to a reduction in the economic costs derived from oncological processes, having a positive economic and social impact.

**4.3. Project feasibility and applicability**

Regarding the feasibility of this study, both radiofrequency and pelvic floor muscle training are techniques that have shown scientific evidence in patients with genitourinary symptoms not

diagnosed with breast cancer. For this reason, it is suggested that these physiotherapy techniques be implemented in this population with the aim of finding out the effects they produce in women diagnosed with breast cancer.

The fact that this study is a continuation of a previous study carried out by the group (IDOC22‐07) in which the pelvic dysfunction presents in breast cancer patients associated with AMACMEC was characterised, where information and awareness workshops on pelvic floor dysfunctions were also developed, ensures that the sample proposed for this study was reached in the different intervention groups.

The study will have a direct clinical application in the event that positive results can be obtained in pelvic floor dysfunctions, as well as in the improvement of the quality of life and self‐perception of these women. The achievement of this study will allow the design of new studies to collaborate with this association, as well as to incorporate other national associations of breast cancer patients and associations related to other types of cancer where the evolution of the disease and the treatments used have a similar impact on the pelvic floor.

1. **ANNEXES**

**Annex 1. Patient information sheet**

**Annex 2. Informed Consent**

**Annex 3. Questionnaire**

**Annex 4. Pelvic floor musculature strength record sheet**

1. **BIBLIOGRAPHY**

1.Cancer Today [Internet]. [citado 22 de abril de 2024]. Disponible en: <https://gco.iarc.who.int/today/>

2.Dimensiones del cáncer | AECC Observatorio [Internet]. [citado 22 de abril de 2024]. Disponible en: <https://observatorio.contraelcancer.es/explora/dimensiones-del-cancer>.

3.Cáncer de mama [Internet]. [citado 22 de abril de 2024]. Disponible en: <https://www.who.int/es/news-room/fact-sheets/detail/breast-cancer>

4.Toxicidad financiera del cáncer de mama | AECC Observatorio [Internet]. [citado 22 de abril de 2024]. Disponible en: <https://observatorio.contraelcancer.es/informes/toxicidad-financiera-del-cancer-de-mama>

5.Knaul FM, López Carrillo L, Lazcano Ponce E, Gómez Dantés H, Romieu I, Torres G. Cáncer de mama: un reto para la sociedad y los sistemas de salud. Salud Pública México. enero de 2009;51:s138-40.

6.Marzo-Castillejo M, Vela-Vallespín C, Bellas-Beceiro B, Bartolomé-Moreno C, Melús-Palazón E, Vilarrubí-Estrella M, et al. Marzo-Castillejo M, Vela-Vallespín C, Bellas-Beceiro B, Bartolomé-Moreno C, Melús-Palazón E, Vilarrubí-Estrella M, Nuin-Villanueva M. Recomendaciones de prevención del cáncer. Actualización PAPPS 2018;50 Suppl 1(Suppl 1):41-65. Spanish. doi: 10.1016/S0212-6567(18)30362-7.

7.Rui L, Guijuan Z, Fengjie B. Eugenol supresses the development of estrogen receptor-positive precan- cerous breast lesions and regulates estrogen receptor-related proteins. Acta Medica Mediterr.2018;(6):1821-7.

8.Boswell EN, Dizon DS. Breast cancer and sexual function. Transl Androl Urol. 2015;4(2):160-8.

9.WHO-MHP-HPS-EML-2021.02-eng.pdf [Internet]. [citado 23 de mayo de 2024]. Disponible en: <https://iris.who.int/bitstream/handle/10665/345533/WHO-MHP-HPS-EML-2021.02-eng.pdf>

10.Vivar CG. Impacto psicosocial del cáncer de mama en la etapa de larga supervivencia: propuesta de un plan de cuidados integral para supervivientes. Aten Primaria. 2012;44(5):288-92.

11.Jun EY, Kim S, Chang SB, Oh K, Kang HS, Kang SS. The effect of a sexual life reframing program on marital intimacy, body image, and sexual function among breast cancer survivors. Cancer Nurs. 2011;34(2):142-9.

12.Quality of Life after Breast Cancer: Survivorship and Sexuality - Dizon - 2009 - The Breast Journal - Wiley Online Library [Internet]. [citado 22 de abril de 2024]. Disponible en: <https://onlinelibrary.wiley.com/doi/10.1111/j.1524-4741.2009.00766.x>

13.Abdi F, Rahnemaei FA, Roozbeh N, Pakzad R. Impact of phytoestrogens on treatment of urogenital menopause symptoms: A systematic review of randomized clinical trials. Eur J Obstet Gynecol Reprod Biol. 2021;261:222-35.

14.Crean-Tate KK, Faubion SS, Pederson HJ, Vencill JA, Batur P. Management of genitourinary syndrome of menopause in female cancer patients: a focus on vaginal hormonal therapy. Am J Obstet Gynecol. 2020;222(2):103-13.

15.Portman DJ, Gass MLS, Vulvovaginal Atrophy Terminology Consensus Conference Panel. Genitourinary syndrome of menopause: new terminology for vulvovaginal atrophy from the International Society for the Study of Women’s Sexual Health and the North American Menopause Society. Menopause N Y N. 2014;21(10):1063-8.

16.Colombage UN, Lin KY, Soh SE, Frawley HC. Prevalence and impact of bladder and bowel disorders in women with breast cancer: A systematic review with meta-analysis. Neurourol Urodyn. 2021;40(1):15-27.

17.Sousa M, Peate M, Lewis C, Jarvis S, Willis A, Hickey M, et al. Exploring knowledge, attitudes and experience of genitourinary symptoms in women with early breast cancer on adjuvant endocrine therapy. Eur J Cancer Care (Engl). 2018;27(2):e12820.

18.Córdoba-de Juan C, Arranz-Martín B, Torres-Lacomba M. Disfunción sexual en mujeres diagnosticadas y tratadas de cáncer de mama. Estudio descriptivo longitudinal. Fisioterapia. 2019;41(2):73-82.

19.Pennycuff JF, Desale S, Wang H, Zhang G, Richter LA. Prevalence of pelvic floor disorders, associations of endocrine therapy, and surgical intervention among breast cancer survivors. Int Urogynecology J. 2022;33(9):2421-6.

20.Robinson PJ, Bell RJ, Christakis MK, Ivezic SR, Davis SR. Aromatase Inhibitors Are Associated With Low Sexual Desire Causing Distress and Fecal Incontinence in Women: An Observational Study. J Sex Med. 2017;14(12):1566-74.

21.Stahlschmidt R, Ferracini AC, Medeiros LM de, Souza CM de, Juliato CRT, Mazzola PG. Urinary Incontinence and Overactive Bladder Symptoms in Women with Breast Cancer Being Treated with Oral Hormone Therapy. Rev Bras Ginecol E Obstet Rev Fed Bras Soc Ginecol E Obstet. 2020;42(11):726-30.

22.Cárcamo M, Baquedano H, Díaz D, Díaz G P. Caracterización de incontinencia urinaria en mujeres premenopáusicas con cáncer de mama en tratamiento con tamoxifeno por cinco años en el Instituto Nacional del Cáncer. Rev Médica Clínica Las Condes. 2020;31(3):352-7.

23.Oberguggenberger A, Martini C, Huber N, Fallowfield L, Hubalek M, Daniaux M, et al. Self-reported sexual health: Breast cancer survivors compared to women from the general population - an observational study. BMC Cancer. 2017;17(1):599.

24 .Crandall CJ, Mehta JM, Manson JE. Management of Menopausal Symptoms: A Review. JAMA. 2023;329(5):405-20.

25.Riggs JA. Retropubic cystourethropexy: a review of two operative procedures with long-term follow-up. Obstet Gynecol. 1986;68(1):98-105.

26.Lapitan MCM, Cody JD, Grant A. Open retropubic colposuspension for urinary incontinence in women. Cochrane Database Syst Rev. 2009;(2):CD002912.

27.Intervenciones con cabestrillos mediouretrales para la incontinencia urinaria de esfuerzo en mujeres [Internet]. [citado 24 de abril de 2024]. Disponible en: <https://www.cochrane.org/es/CD006375/INCONT_intervenciones-con-cabestrillos-mediouretrales-para-la-incontinencia-urinaria-de-esfuerzo-en-mujeres>

28.McGuire EJ. Urethral bulking agents. Nat Clin Pract Urol. 2006;3(5):234-5.

29.Kuuva N, Nilsson CG. A nationwide analysis of complications associated with the tension-free vaginal tape (TVT) procedure. Acta Obstet Gynecol Scand. 2002;81(1):72-7.

30.Nilsson CG, Falconer C, Rezapour M. Seven-year follow-up of the tension-free vaginal tape procedure for treatment of urinary incontinence. Obstet Gynecol. 2004;104(6):1259-62.

31.Eisner H, McIntosh GV. Pubovaginal Sling. En: StatPearls [Internet]. Treasure Island (FL): StatPearls Publishing; 2024 [citado 25 de abril de 2024]. Disponible en: http://www.ncbi.nlm.nih.gov/books/NBK572150/

32.Santen RJ, Stuenkel CA, Davis SR, Pinkerton JV, Gompel A, Lumsden MA. Managing Menopausal Symptoms and Associated Clinical Issues in Breast Cancer Survivors. J Clin Endocrinol Metab. 2017;102(10):3647-61.

33.Shifren JL. Genitourinary Syndrome of Menopause. Clin Obstet Gynecol. 2018;61(3):508-16.

34.Lukacz ES, Santiago-Lastra Y, Albo ME, Brubaker L. Urinary Incontinence in Women: A Review. JAMA. 2017;318(16):1592-604.

35.The 2023 Nonhormone Therapy Position Statement of The North American Menopause Society. Advisory Panel. The 2023 nonhormone therapy position statement of The North American Menopause Society. Menopause N Y N. 2023;30(6):573-90.

36.Menown SJ, Tello JA. Neurokinin 3 Receptor Antagonists Compared With Serotonin Norepinephrine Reuptake Inhibitors for Non-Hormonal Treatment of Menopausal Hot Flushes: A Systematic Qualitative Review. Adv Ther. 2021;38(10):5025-45.

37.Wiley.com [Internet]. [citado 25 de abril de 2024]. Female Sexual Pain Disorders: Evaluation and Management, 2nd Edition | Wiley. Disponible en: <https://www.wiley.com/en-us/Female+Sexual+Pain+Disorders%3A+Evaluation+and+Management%2C+2nd+Edition-p-9781119482598>

38.Colombage UN, Soh SE, Lin KY, Kruger J, Frawley HC. The feasibility of pelvic floor training to treat urinary incontinence in women with breast cancer: a telehealth intervention trial. Breast Cancer Tokyo Jpn. 2023;30(1):121-30.

39.Seav SM, Dominick SA, Stepanyuk B, Gorman JR, Chingos DT, Ehren JL, et al. Management of sexual dysfunction in breast cancer survivors: a systematic review. Womens Midlife Health. 2015;1:9.

40.Pérez-López FR, Phillips N, Vieira-Baptista P, Cohen-Sacher B, Fialho SCAV, Stockdale CK. Management of postmenopausal vulvovaginal atrophy: recommendations of the International Society for the Study of Vulvovaginal Disease. Gynecol Endocrinol Off J Int Soc Gynecol Endocrinol. 2021;37(8):746-52.

41.Schvartzman R, Schvartzman L, Ferreira CF, Vettorazzi J, Bertotto A, Wender MCO. Physical Therapy Intervention for Women With Dyspareunia: A Randomized Clinical Trial. J Sex Marital Ther. 2019;45(5):378-94.

42.The NAMS 2020 GSM Position Statement Editorial Panel. The 2020 genitourinary syndrome of menopause position statement of The North American Menopause Society. Menopause N Y N. 2020;27(9):976-92.

43.Espitia de la Hoz FJ, Orozco Gallego H. Abordaje diagnóstico y terapéutico del síndrome genitourinario en la menopausia; actualización. Rev Médica Univ Costa Rica. 2017;11(2):67-84.

44.Mercier J, Morin M, Zaki D, Reichetzer B, Lemieux MC, Khalifé S, et al. Pelvic floor muscle training as a treatment for genitourinary syndrome of menopause: A single-arm feasibility study. Maturitas. 2019;125:57-62.

45.Kamilos MF, Borrelli CL. New therapeutic option in genitourinary syndrome of menopause: pilot study using microablative fractional radiofrequency. Einstein Sao Paulo Braz. 2017;15(4):445-51.

46.Sarmento AC, Fernandes FS, Marconi C, Giraldo PC, Eleutério-Júnior J, Crispim JC, et al. Impact of microablative fractional radiofrequency on the vaginal health, microbiota, and cellularity of postmenopausal women. Clin Sao Paulo Braz. 2020;75:e1750.

47.Slongo H, Lunardi ALB, Riccetto CLZ, Machado HC, Juliato CRT. Microablative radiofrequency versus pelvic floor muscle training for stress urinary incontinence: a randomized controlled trial. Int Urogynecology J. 2022;33(1):53-64.

48.Derbyshire M. Radiofrecuencia fraccionada microablativa como opción terapéutica para el liquen escleroso vulvar: estudio piloto [Internet]. International Menopause Society. 2022 [citado 23 de mayo de 2024]. Disponible en: <https://www.imsociety.org/2022/08/09/radiofrecuencia-fraccionada-microablativa-como-opcion-terapeutica-para-el-liquen-escleroso-vulvar-estudio-piloto/>

49.Millheiser LS, Pauls RN, Herbst SJ, Chen BH. Radiofrequency treatment of vaginal laxity after vaginal delivery: nonsurgical vaginal tightening. J Sex Med. 2010;7(9):3088-95.

50.Wańczyk-Baszak J, Woźniak S, Milejski B, Paszkowski T. Genitourinary syndrome of menopause treatment using lasers and temperature-controlled radiofrequency. Przegla̜d Menopauzalny Menopause Rev. 2018;17(4):180-4.

51.Sánchez-Sánchez B, Torres-Lacomba M, Yuste-Sánchez MJ, Navarro-Brazález B, Pacheco-da-Costa S, Gutiérrez-Ortega C, et al. Cultural adaptation and validation of the Pelvic Floor Distress Inventory short form (PFDI-20) and Pelvic Floor Impact Questionnaire short form (PFIQ-7) Spanish versions. Eur J Obstet Gynecol Reprod Biol. 2013;170(1):281-5.

52.Lalji S, Lozanova P. Evaluation of the safety and efficacy of a monopolar nonablative radiofrequency device for the improvement of vulvo‐vaginal laxity and urinary incontinence. J Cosmet Dermatol. 2017;16(2):230-4.

53.Pinheiro C, Costa T, Amorim de Jesus R, Campos R, Brim R, Teles A, et al. Intravaginal nonablative radiofrequency in the treatment of genitourinary syndrome of menopause symptoms: a single-arm pilot study. BMC Womens Health. 2021;21(1):379.

54.Zachovajeviene B, Siupsinskas L, Zachovajevas P, Venclovas Z, Milonas D. Effect of diaphragm and abdominal muscle training on pelvic floor strength and endurance: results of a prospective randomized trial. Sci Rep. 2019; 9(1):19192.

55.Molina-Torres G, Moreno-Muñoz M, Rebullido TR, Castellote-Caballero Y, Bergamin M, Gobbo S, Hita-Contreras F, Cruz-Diaz D. The effects of an 8-week hypopressive exercise training program on urinary incontinence and pelvic floor muscle activation: A randomized controlled trial. Neurourol Urodyn. 2023;42(2):500-509.

56.Skaug KL, Engh ME, Bø K. Pelvic floor muscle training in female functional fitness exercisers: an assessor-blindedrandomised controlled trial. Br J Sports Med. 2024.

57. Jorge CH, Bø K, Chiazuto Catai C, Oliveira Brito LG, Driusso P, Kolberg Tennfjord M. Pelvic floor muscle training as treatment for female sexual dysfunction: a systematic review and meta-analysis. Am J Obstet Gynecol. 2024.

58. Tibaek S, Dehlendorff C. Pelvic floor muscle function in women with pelvic floor dysfunction: a retrospective chart review, 1992-2008. Int Urogynecol J. 2014;25(5):663-9.

59. Gimenez MM, Fitz FF, de Azevedo Ferreira L, Bortolini MAT, Lordêlo PVS, Castro RA. Pelvic floor muscle functiondiffers between supine and standing positions in women with stress urinary incontinence: an experimental crossover study. J Physiother. 2022;68(1):51-60.

60. McClurg D, Frawley H, Hay-Smith J, Dean S, Chen SY, Chiarelli P, Mair F, Dumoulin C. Scoping review of adherencepromotion theories in pelvic floor muscle training - 2011 ICS state-of-the-science seminar research paper i of iv. Neurourol Urodyn. 2015;34(7):606-14.

61. Dakic JG, Cook J, Hay-Smith J, Lin KY, Frawley H. Pelvic floor disorders stop women exercising: A survey of 4556 symptomatic women. J Sci Med Sport. 2021;24(12):1211-1217.

62. Sapsford RR, Hodges PW, Richardson CA, Cooper DH, Markwell SJ, Jull GA. Co-activation of the abdominal and pelvic floor muscles during voluntary exercises. Neurourol Urodyn. 2001; 20(1):31-42.

63. Radzimińska A, Strączyńska A, Weber-Rajek M, Styczyńska H, Strojek K, Piekorz Z. The impact of pelvic floor muscle training on the quality of life of women with urinary incontinence: a systematic literature review. Clin Interv Aging. 2018; 13:957-965.

64.Sapsford RR, Richardson CA, Stanton WR. Sitting posture affects pelvic floor muscle activity in parous women: an observational study. Aust J Physiother. 2006;52(3):219-22.

65.Fisiología Clínica del Ejercicio de José López Chicharro | España | Editorial Médica Panamericana [Internet]. [citado 23 de mayo de 2024]. Disponible en: https://www.medicapanamericana.com/es/libro/fisiologia-clinica-del-ejercicio

66.Romero-Cullerés G, Peña-Pitarch E, Jané-Feixas C, Arnau A, Montesinos J, Abenoza-Guardiola M. Intra-rater reliability and diagnostic accuracy of a new vaginal dynamometer to measure pelvic floor muscle strength in women with urinary incontinence. Neurourol Urodyn. 2017;36(2):333-7.

67. Tibaek, S., Gard, G., Dehlendorff, C., Iversen, H. K., Erdal, J., Biering-Sorensen, F. Jensen, R. 2015. The effect of pelvic floor muscle training on sexual function in men

with lower urinary tract symptoms after stroke. Topics in Stroke Rehabilitation, 22, 185-193.

68.Kenney WL, Wilmore JH, Costill DL, Wilmore JH. Physiology of sport and exercise. 5th ed. Champaign, IL: Human Kinetics; 2012. 621.

69. Laycock, J. Clinical evaluation os the pelvic floor. In B. Schüssler, J. Laycock, P. Norton, & S. L. Stanton (Eds.), Pelvic floor re-education. London, England: Springer-Verlag.1994: 42-28.

70.Tibaek S, Gard G, Dehlendorff C, Iversen HK, Biering-Soerensen F, Jensen R. Is Pelvic Floor Muscle Training Effective for Men With Poststroke Lower Urinary Tract Symptoms? A Single-Blinded Randomized, Controlled Trial. Am J Mens Health. septiembre de 2017;11(5):1460-71.

71. Laycok, J. Haslam, J. Patient assesment in therapeutic management of incontinence and pelvic pain. Springer.2002.

72. Worman R, Stafford R.e., Cowley D, Hodges P.W Methods used to investigate tone of pelvic floor muscles in pelvic health conditions: A systematic review. Elsevier. 2023.

73.Espuña Pons M, Rebollo Alvarez P, Puig Clota M. [Validation of the Spanish version of the International Consultation on Incontinence Questionnaire-Short Form. A questionnaire for assessing the urinary incontinence]. Med Clin (Barc). 2004;122(8):288-92.

74.Sandvik H, Seim A, Vanvik A, Hunskaar S. A severity index for epidemiological surveys of female urinary incontinence: comparison with 48-hour pad-weighing tests. Neurourol Urodyn. 2000;19(2):137-45.

75.Tamanini JTN, Almeida FG, Girotti ME, Riccetto CLZ, Palma PCR, Rios LAS. The Portuguese validation of the International Consultation on Incontinence Questionnaire-Vaginal Symptoms (ICIQ-VS) for Brazilian women with pelvic organ prolapse. Int Urogynecol J Pelvic Floor Dysfunct. 2008;19(10):1385-91.

76. Bachmann G. Urogenital ageing: an old problem newly recognized. Maturitas. diciembre de 1995;22 Suppl:S1-5.

**ANEXES**

**ANNEX 1. INFORMATION SHEET**

**INFORMATION SHEET FOR PARTICIPANTS IN THE RESEARCH PROJECT**

**STUDY TITLE:** Effectiveness of radiofrequency and exercise-based rehabilitation on the symptomatology associated with pelvic floor dysfunctions in breast cancer patients. Randomised clinical trial.

**PRINCIPAL RESEARCHER:** *Ms. Beatriz Pina Bernabeu CEU Cardenal Herrera University*

**INTRODUCTION**

We are writing to inform you about a research study in which you are invited to participate.

Our intention is that you receive sufficient and correct information so that you can decide whether or not to participate in this study. To do this, please take the time to read this information sheet carefully and thoroughly and discuss it with whomever you feel appropriate. Ask the study staff to explain any words or information that you do not understand clearly, as well as any questions you may have.

If you decide that you wish to participate, we will ask you to sign the attached informed consent document. We will provide you with an original copy of this signed and dated document for you to keep and the original document will be kept on file with the rest of the study documentation.

The study has been approved by the Research Ethics Committee of the CEU Cardenal Herrera University of Valencia.

It has also been designed and will be carried out in accordance with the recommendations set out in the Declaration of Helsinki and the Standards of Good Clinical Practice.

You should be aware that your participation in this study is voluntary and that you may decide NOT to participate. If you decide to participate, you can change your decision and withdraw your consent at any time, without altering your relationship with your physiotherapist.

You should also be aware that you may be withdrawn from the study if the sponsor or investigators deem it appropriate, either for safety or other reasons. In either case, you will receive an adequate explanation of the reason for your withdrawal from the study.

**WHY IS THE STUDY BEING CARRIED OUT?**

It is known that pelvic function, vaginal health and sexual function are altered in patients with breast cancer due to the development of the disease and the treatments received. The aim of the study is to determine the effectiveness of physiotherapy treatment based on activation of the pelvic floor muscles using radiofrequency-induced heat and physical exercise to improve the symptoms associated with cancer. These are non-invasive and painless treatments that have been very efficient in the treatment of these symptoms in other groups of women but have not yet been tested in patients with breast cancer.

**HOW WILL THE STUDY BE CONDUCTED?**

A personalised treatment will be conducted by physiotherapists with extensive experience in the pelvic floor clinic. Participants will be randomly assigned to one of three treatment groups: heat stimulation (radiofrequency) (group 1), physical exercise (group 2) or radiofrequency + physical exercise (group 3).

The intervention will take place at the facilities of the Asociación de Mujeres Afectadas por el Cáncer de Mama de Elche y Comarca (AMACMEC), with registered office at Calle Olegario Domarco Seller, 93. Entresuelo de Elche (Alicante), and holder of tax identification number G53270146.

The study will begin in September 2024. Radiofrequency is a therapy based on electrotherapy which consists of applying a porcelain electrode, specifically designed for the treatment of dysfunctions in the urogynaecological area, covered by a latex probe cover and introduced into the vagina, to generate an increase in temperature in the tissues, causing the patient to feel a localised sensation of heat in the area of application, always maintaining a perception of intense, non-burning heat. The radiofrequency treatment will consist of 5 sessions of 30 minutes with an interval of 7 days between each one. The physical exercise will consist of a programme of exercises that include pelvic floor activation led by a physiotherapist with a frequency of twice a week, a duration of 45 minutes each day and a period of 16 weeks. In this

case, the patient will be recommended to continue with the exercises at home and will be monitored as to whether or not she complies with the programme at home, this compliance not being a requirement, by filling in a diary which will be provided in paper format for her to complete at home.

**WHAT CRITERIA MUST BE MET IN ORDER TO PARTICIPATE?**

The criteria for participation in the study are: women of legal age with a clinical history of breast cancer and pelvic floor dysfunction such as urinary incontinence, faecal incontinence, prolapse or sexual dysfunction. Women who agree to participate in the study.

The criteria for not being able to participate in the study are: pelvic floor muscle training or radiofrequency sessions within the last 12 months, use of vaginal oestrogen within the last 6 months, systemic hormone therapy within the last 6 months, vaginal laser therapy within the last 6 months, absence of pelvic floor contraction which will be assessed at the time of consent for participation in this study, pacemaker use, decompensated heart or metabolic disease, cognitive deficits, peripheral or central neurological disorders, previous surgeries in the pelvic region, skin pathologies or wounds in the treatment area or presence of an active urinary tract and/or vaginal infection.

**WHAT IS MY PARTICIPATION?**

If you agree to participate in the study, we will ask you:

- Sign the informed consent form. All information collected will be treated confidentially and its use will be purely scientific, safeguarding your identity at all times. This will require you to sign your express consent by means of written authorisation.
- Once participation has been accepted, an initial clinical examination will be carried out to assess the strength of the pelvic floor muscles, as well as to acquire socio- demographic data (age, weight, work, education), data related to the diagnosis and treatment of breast cancer, pelvic function and its impact on quality of life, vaginal status, sexual function and self-esteem using standard questionnaires validated for this type of study.
- You will be randomly assigned to one of the three intervention groups.
- A clinical check-up as described above will be done again 1 week after the end of treatment, 6 months and 1 year after the end of treatment.

**WHAT RISKS OR DISCOMFORTS MIGHT I SUFFER FROM PARTICIPATING IN THE STUDY?**

These are conservative, non-invasive and painless treatments.

With regard to non-ablative radiofrequency, the way it works is by selectively increasing the temperature of the tissues in a controlled manner. In this study, the temperature will not exceed 41ºC, so its possible adverse effects will be almost completely reduced, which could be small erythema or oedema due to the heating of the skin. Although these adverse effects are not related to this low temperature, if they occur they would disappear in 1 or 2 days.

With regard to physical exercise, this will be directed by a professional, guaranteeing the correct performance of each exercise, thus reducing the appearance of potential injuries or pain due to the incorrect practice of physical exercise. On the contrary, it is established that the practice of physical exercise is a recommendation for physical and mental health.

**WHAT BENEFITS WILL BE GAINED FROM THE STUDY?**

The participant will receive a complete non-invasive and painless treatment which, according to previous studies in other groups with similar symptoms, will have a positive impact on her pelvic function and vaginal condition, and will therefore have a positive impact on her quality of life, sexual function and her perception and self- esteem.

The results will make it possible to compare the efficacy of safe, non-invasive and low- cost treatments, obtaining data of great clinical value by allowing the development of conservative therapy programmes for the treatment of symptomatology characteristic of patients who have suffered the consequences of breast cancer and its treatment.

**WHAT TREATMENT OPTIONS DO I HAVE IF I DO NOT PARTICIPATE IN THE STUDY?**

This section is not relevant, as the breast cancer patients have already received their treatment from the medical team that treated them.

**HOW ARE MY RIGHTS PROTECTED?**

The study will be conducted in compliance with all applicable ethical and legal standards.

**Confidentiality**

The researchers undertake that your personal data will be treated confidentially and will be processed in accordance with current legislation on personal data protection (Organic Law 3/2018, on the Protection of Personal Data and Guarantee of Digital Rights, and Regulation [EU] 2016/679 of the European Parliament and of the Council on the protection of natural persons with regard to the processing of personal data and on the free movement of such data). To comply with this regulation, the data collected for the study will be identified by a code, so that it does not include information that can identify you, and only the researchers will be able to relate this data to you and your medical history. Therefore, your identity will not be disclosed to any person except in the case of a medical emergency or legal requirement. The processing, communication and transfer of personal data of all participants will be in accordance with the provisions of the law.

Access to your personally identifiable information will be restricted to investigators, health authorities, the Research Ethics Committee and personnel authorised by the sponsor (study monitors, auditors), when needed to verify study data and procedures, but always maintaining confidentiality in accordance with current legislation.

Questionnaires containing personal data will be kept under lock and key at all times and only researchers will have access to them.

The data will be collected anonymously in a research file under the responsibility of the researchers and will be processed on network computers that can only be accessed with a personal password.

In accordance with the provisions of data protection legislation, you may exercise your rights of access, modification, opposition and cancellation of data by contacting your study doctor.

If you decide to withdraw your consent to participate in this study, no new data will be added to the database, but data already collected will be used.

The coded data may be passed on to third parties, but under no circumstances will they contain information that can directly identify you, such as name and surname, initials, medical history number, etc. In the event of this transfer, it will be for the same purposes of the study described or for use in scientific publications, but always maintaining the confidentiality of the same in accordance with current legislation.

The data collected will never be used for any other purpose, therefore all data will be destroyed and/or deleted once the research results have been published in scientific journals.

**WHO CAN I CONTACT IN CASE OF DOUBT?**

If you have any questions, please contact Cristina Orts Ruiz (CEU Cardenal Herrera University), on telephone number 965 42 64 86 Extension: 403, who is responsible for this research and who will answer any questions you may have regarding this study. Whatever your decision, the research team would like to thank you for your time and attention.

**ANNEX 2**

**CONSENT FORM FOR PARTICIPATION IN A RESEARCH PROJECT**

***PROJECT TITLE: Effectiveness of radiofrequency and exercise-based rehabilitation on symptoms associated with pelvic floor dysfunctions in breast cancer patients. Randomised clinical trial***

***PRINCIPAL INVESTIGATOR. Dr. Cristina Orts Ruiz***

**Dr._______________________________________________________**

**With DNI Nº____________________**

**Freely and voluntarily**

**I DECLARE:**

1. I have read and understood the information sheet that is the subject of the study.
2. I have had the opportunity to ask questions.
3. My questions have been answered satisfactorily.
4. I have received sufficient information about the study and the tests to be performed.
5. I understand that participation is voluntary and I may leave the study at any time without explanation and without affecting my medical care.
6. In accordance with the provisions of Regulation (EU) 2016/679 of the European Parliament and of the Council of 27 April 2016 on the protection of individuals with regard to the processing of personal data and on the free movement of such data and repealing Directive 95/46/EC, I have been informed that my personal data, obtained through the completion of this form as well as those resulting from my participation in the project will be processed under the responsibility of the FUNDACIÓN UNIVERSITARIA SAN PABLO CEU (hereinafter, FUSP-CEU), for the purpose of managing my participation in this research project. In addition, I have been informed of the following aspects:
   1. That profiling is envisaged for the purpose of analysing or predicting aspects of my health.
   2. That the aforementioned processing is legitimised by the consent given by me.
   3. That my personal data, obtained by filling in this form, as well as those resulting from my participation in the project will be kept for the time necessary for the development of this research, which is estimated to be 10 months, and will subsequently be destroyed, without being able to be kept without having been previously anonymised. In any case, they may not be transferred without my express consent, which I do not give in this act.
   4. That I can contact the Data Protection Delegate of FUSP-CEU, by sending my request in writing to the postal address C/ Tutor nº 35 - 28008 Madrid or to the e- mail address [.dpd@ceu.es](mailto:.dpd@ceu.es)
   5. That in accordance with the rights conferred on me by current data protection legislation, I may contact the competent Control Authority to file the claim I

consider appropriate, and I may also exercise my rights of access, rectification, limitation of processing, deletion, portability and opposition to the processing of my personal data and withdraw the consent given for the processing of the same, by addressing my request to the researcher responsible at the contact address given in this document.

1. I agree that my written consent and other data will be made available to the clinical research project in which I am participating, and to the researcher responsible for it, Dr. Cristina Orts Ruiz, but always respecting confidentiality and the guarantee that my data will not be publicly available in a way that could lead to my identification.
2. The data collected for this study will be included, together with those of other persons participating in this study, in a personal database of the CEU Cardenal Herrera University, to which only the researchers approved for this project will have access, all of them being subject to the secrecy inherent to their profession or derived from a confidentiality agreement.
3. I am signing this information and consent document voluntarily to indicate my willingness to participate in this research study on RADIOFREQUENCY AND THERAPEUTIC PHYSICAL EXERCISE FOR GENITOURINARY SYNDROME OR PELVIC FOUNDATION DYSFUNCTION AFTER BREAST CANCER, until I decide

otherwise. By signing this consent I do not waive any of my rights. I will receive a copy of this document for my records for future reference.

I therefore agree and consent to the detailed study being carried out with the assistance of suitably qualified and specialised personnel as required.

The participant

(Signature) Name, Surname

Valencia, at ……… of ………………… of 2024

**AUTHORISATION OF THE RELATIVE OR GUARDIAN**

In view of the impossibility of Mr./Mrs.

with DNI to freely, voluntarily and knowingly give authorisation for the processing of the data described in this document.

Mr./Ms. with DNI

As (husband, wife, child, sibling, legal guardian, relative, relative, caregiver), I decide, within the clinical options available, to give my free, voluntary and informed consent to the technique described for the treatments described in this document.

, de de

**RESEARCHER**

Ms. Dr. Cristina Orts Ruiz with DNI: 74241971H

E-mail: [cristina.orts@uchceu.es](mailto:cristina.orts@uchceu.es) Telephone: 96 542 64 86 | Ext. 67403

Researcher at the CEU-Cardenal Herrera University of Valencia, I declare that I have provided the study participant and/or authorised person with all the information necessary to carry out the intervention described in this document and I declare that I have confirmed, immediately prior to the application of the technique, that the participant does not have any of the contraindications listed above, and that I have taken all the necessary precautions to ensure that the intervention is carried out correctly.

, de de

**REVOCATION OF INFORMED CONSENT**

Mr/Ms. with DNI

I revoke the consent given on of of

And I do not wish to continue the treatment which I hereby terminate.

, de de

**ANNEX 3 AD HOC QUESTIONNAIRE**

**QUESTIONNAIRE TO STUDY THE EFFECTIVENESS OF RADIOFREQUENCY AND EXERCISE-BASED REHABILITATION ON SYMPTOMS ASSOCIATED WITH PELVIC FLOOR DYSFUNCTIONS IN BREAST CANCER PATIENTS. RANDOMISED CLINICAL TRIAL**

Patient identification code:

1. Age (years):
2. Weight (Kg):
3. Height (metres):
4. Nationality:
5. Country of residence:
6. Higher level of education attained:
   1. *Compulsory Secondary Education (ESO)*
   2. *Basic Vocational Education and Training (VET)*
   3. *Baccalaureate*
   4. *Intermediate level training courses*
   5. *Higher level training courses*
   6. *University studies*
   7. *Uneducated*
7. Are you currently working?
   1. *Yes*
   2. *Retired*
   3. *Unemployed*
   4. *Student*
8. Type of work:
   1. *I do not work*
   2. *Sanitary*
   3. *Social humanities*
   4. *Services*
9. Number of pregnancies carried to term
10. Number of pregnancies not carried to term
11. If you have been pregnant, please indicate the age at which you had your first pregnancy
12. Indicate the number of vaginal deliveries
13. Indicate the number of caesarean sections
14. Have you been breastfeeding?
    1. *Yes*
    2. *No*
    3. *Only some of my children*
15. Do you have menopause?
    1. *Yes, since before cancer treatment for breast cancer*
    2. *Yes, since after cancer treatment for breast cancer*
    3. *No*
16. If yes, please indicate how long you have been in menopause
17. Do you smoke?
    1. *Yes*
    2. *No*
    3. *Occasionally*
18. Do you drink alcohol?
    1. *Yes*
    2. *No*
    3. *Occasionally*
19. Please indicate how often you have practised moderate‐high intensity sport during the last 3 months (equivalent or more than a brisk walk of at least 30min).
    1. *Every day*
    2. *2‐4 times per week*
    3. *Less than 2 times per week*
    4. *I do not play sport*
20. How many years ago were you first diagnosed with breast cancer?
21. *What kind of treatment have you received for breast cancer?*
22. *Chemotherapy*
23. *Radiotherapy*
24. *Hormone Therapy*
25. *Breast surgery, without reconstruction*
26. *Breast surgery, with reconstruction*
27. *Axillary surgery*
28. *Immunotherapy*
29. *None*
30. Are you currently undergoing treatment for breast cancer?
    1. *Yes*
    2. *No*
31. If you are currently receiving treatment, please indicate which one you are receiving
    1. *Chemotherapy*
    2. *Radiotherapy*
    3. *Hormone therapy*
    4. *Immunotherapy*
32. Please indicate which of the following symptoms you have experienced following treatment (tick as many as you can think of)
    1. *Upper limb pain*
    2. *Lymphoedema*
    3. *Superficial lymphatic thromboses*
    4. *Fatigue*
    5. *Urinary incontinence*
    6. *Faecal incontinence*
    7. *Pelvic pain*
    8. *Sexual dysfunction*
    9. *None*
33. Indicate if you have any of the following symptoms (tick as many as you think you have)
    1. *Vaginal dryness*
    2. *Pain during sexual intercourse*
    3. *Reduced vaginal laxity (elasticity)*
    4. *Vaginal itching*
    5. *Burning sensation*
    6. *Pain at the vaginal entrance*
34. Have you had a recurrence of breast cancer?
35. *Yes*
36. *No*
37. Have you had any other type of cancer?
38. *Yes*
39. *No*
40. If you have had another type of cancer, which one(s)?
41. How many years ago were you diagnosed with this cancer?
42. what kind of treatment have you received for this other type of cancer:
    1. *Chemotherapy*
    2. *Radiotherapy*
    3. *Hormone therapy*
    4. *Surgery*
    5. *Immunotherapy*
    6. *None*
43. Are you receiving psychological treatment?
    1. *Yes, as a result of the diagnosis of breast cancer.*
    2. *Yes, for causes other than breast cancer*
    3. *No*
44. Are you receiving or have you received physiotherapy treatment as a result of breast cancer?
    1. *Yes, currently*
    2. *Yes, but not currently*
    3. *No*
45. Have you done pelvic floor rehabilitation after cancer?
    1. *Yes, currently*
    2. *Yes, but not currently*
    3. *No*
46. Do you work on your pelvic floor through exercises?
    1. *Yes, I am currently working on it*
    2. *No, but I have worked on it*
    3. *No*
47. Have you ever received oestrogen treatment?
48. *Yes, currently*
49. *Yes, but not currently*
50. *No*

**ANNEX 4. PHYSICAL EXERCISE PROGRAMME**

**Physical Exercise Programme focused on the pelvic floor.**

The first 4 weeks will include one day of CORE and pelvic floor training in clinic and individual session to ensure understanding of concepts and correct performance of the technique. After this, the therapeutic exercise protocol will be based on proprioception, mobilisation and activation of the structures responsible for the CORE, isometric work, voluntary activation of the CORE muscles, pelvic floor and synergistic muscles such as the gluteus. The work will be both static and dynamic. The exercises will be performed in resisted exhalation and apnoea to facilitate the activation of the lumbo-pelvic complex, as previous programmes for patients with abdomino-pelvic dysfunctions have already demonstrated their effectiveness.

The individual pictured in the physical exercise programme description has provided written informed consent (as outlined in PLOS consent form) to publish their image alongside the manuscript.

| ***Ejercicios*** | ***Descripción*** | ***Intensidad y frecuencia*** | ***Ilustraciones*** |
| --- | --- | --- | --- |
| **MONTH 1, Day 1** | | | |
| Individual session in a cabin, where anatomical and breathing concepts will be explained and how to carry out the exercises will be explained, checking their correct performance by means of a digital test. The number of sustained and rapid contractions and apnoea time will be determined according to the initial assessment. | | | |
| **MONTH 1, Days 2, 4, 6, 8** | | | |
| **Pelvic floor and transverse floor activation.** | Face up, legs flexed with heel support and dorsal flexion of the ankle. In resisted exhalation, self- elongation and activation of the transversus abdominis and pelvic floor. | 10 breaths. | 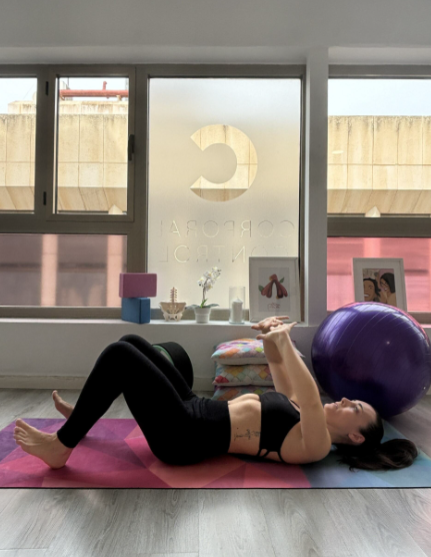 |
| **Gluteal bridge.** | Face up, legs flexed with heel support and dorsal flexion of the ankle. In resisted exhalation, self- elongation and activation of the transversus abdominis and pelvic floor together with gluteal elevation (gluteal bridge). | 10 repetitions. | 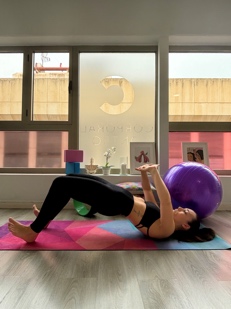 |
| **Gluteal bridge with alternate leg extension.** | Face up, legs bent with heel support. In resisted exhalation, self-elongation and activation of the  transverse and pelvic floor and lifting of the buttocks (gluteal bridge) with unilateral leg stretch. Same exercise with the other leg. | 10 repetitions. | 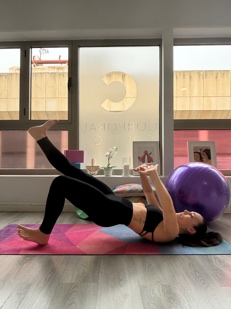 |
| **V-bend abdominals with ball.** | Face up, legs at 90 degrees with ball between them, hip and knee flexion-extension movement is performed with adductor activation.  This is performed with exhalation on descent and stretching of the legs. | 10 repetitions. | 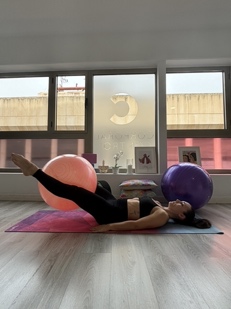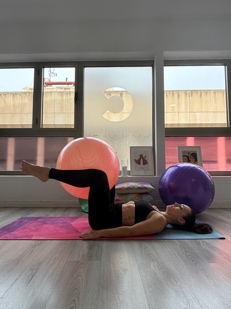 |
| **Leg raise lying on the side.** | On the side with legs straight, activation of the transverse and pelvic floor, perform lifting and lowering movement of the upper leg with maximum amplitude and slow speed and then with minimum amplitude of the movement and maximum speed. | 10 repetitions. | 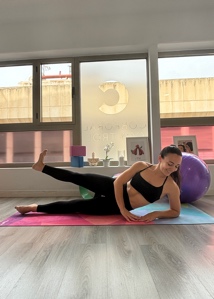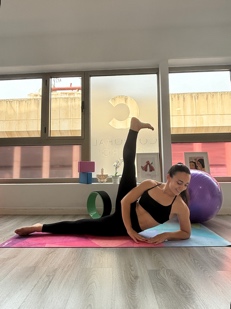 |
| **Leg circling sideways.** | On the side with legs stretched out, activation of the transversus abdominis and awareness of the pelvic floor, perform a circling movement with the upper  leg in both directions. | 10 repetitions. | 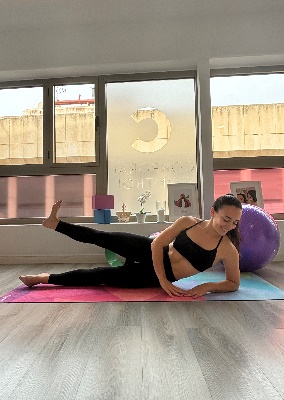 |
| **Side leg flexion and extension.** | On the side, legs aligned with the trunk, activate the transverse and pelvic floor, perform hip and knee flexion and extension movements. | 10 repetitions. | 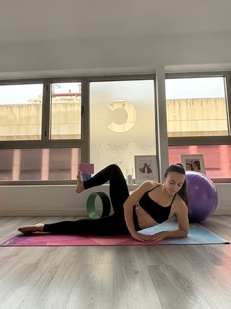 |
| **Sitting contractions on Fitball.** | Sitting on the Fitball, perform pelvic floor contractions with activation of the transversus abdominis, fast and sustained in expiratory time. Do not stay in apnoea. | According to the assessment parameters that we made in consultation. | 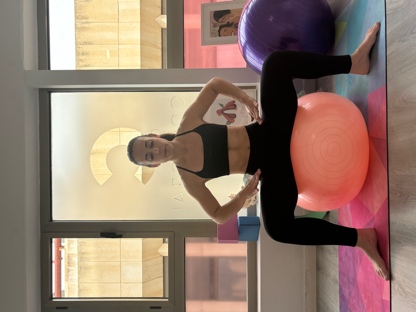 |
| **Pelvic mobility on Fitball.** | On Fitball, awareness and pelvic proprioception movements (perform circles in both directions and infinite circles) to give mobility to the pelvis and relax the CORE and pelvic floor. | 60 seconds. | 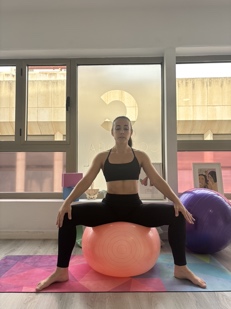 |
| **MONTH 1, Days 3, 5, 7** | | | |
| **Pelvic mobility on Fitball.** | On Fitball, awareness and pelvic proprioception movements (perform circles in both directions and infinite circles) to give mobility to the pelvis and relax the CORE and pelvic floor. | 60 seconds. | 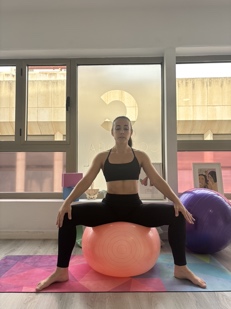 |
| **Pelvic contractions on Fitball.** | Sitting on the Fitball, perform pelvic floor contractions with activation of the transversus abdominis, fast and sustained in expiratory time. Do not stay in apnoea. | According to the assessment parameters that we made in consultation. | 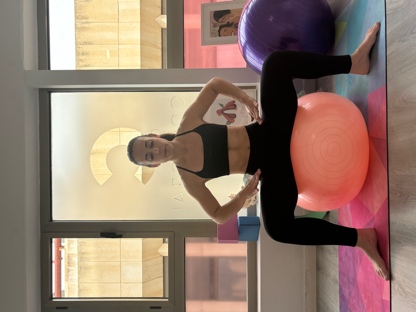 |
| **Self-stretching on Fitball.** | Sitting on the Fitball with heels supported and dorsal flexion of the ankle. After 2 full breaths, stay in apnoea and self-stretch, growing with transverse and pelvic floor activation. | Maintenance of 10sec. | 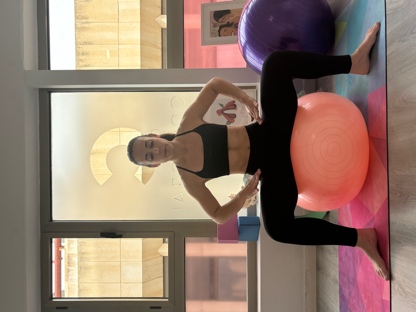 |
| **Self stretching on Fitball with alternating leg lifts.** | Sitting on the Fitball after 2 full breaths, stay in apnoea and self-stretch, growing, with transverse and pelvic floor activation with  alternate leg raises. | 10 sec. maintenance | 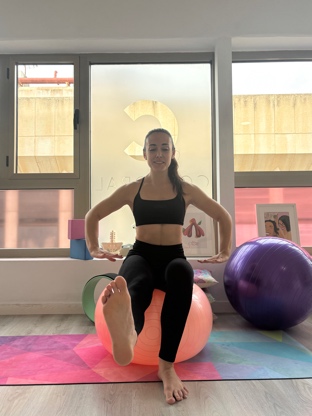 |
| **Quadruped pelvic scales.** | In quadruped movement of pelvic scales with transversus activation and pelvic floor awareness. | 10 repetitions. | 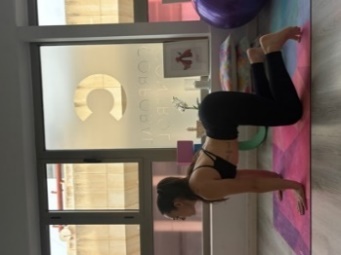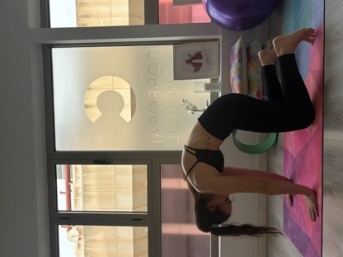 |
| **Lifting of opposite limbs in quadruped.** | In quadruped position, raise one arm and one contralateral leg in resisted expiratory time. Alternate limbs. | 10 repetitions. | 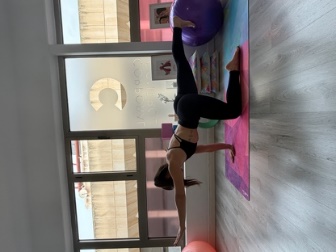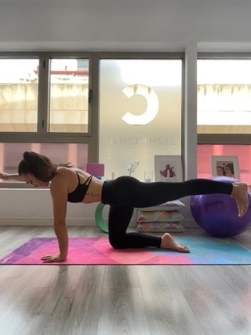 |
| **Hypopressives and self-stretching in quadruped with alternating arm flexion and leg .** | On quadruped, perform hypopressive exercise (after 2 full breaths stay in apnoea, self-elongation and activation of the transverse and pelvic floor and hold for 10 seconds) with flexion of the arms and stretching of one leg.  Repeat with the other leg. | Maintenance of 10sec. | 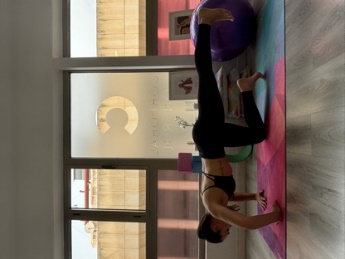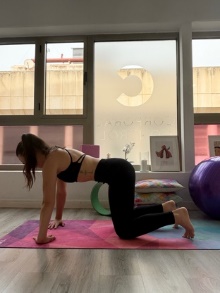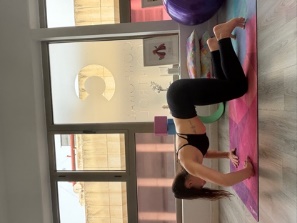 |
| **Mohammedan stance.** | In quadruped position, pelvis mobilisation and  dynamic stretching, ending with the posture of the Mohammedan and stretching of the posterior chain. Sit on your heels, rest your head on the floor and stretch your arms in front of you. |  | 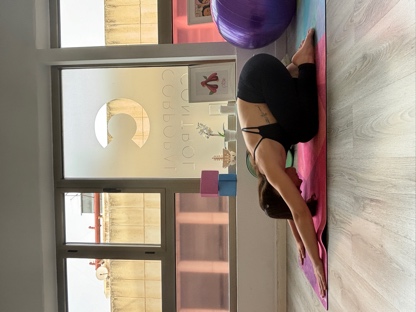 |
| **MONTH 2, Days 9, 11, 13, 15** | | | |
| **Pelvic mobility.** | Standing, pelvic awareness exercise. Make infinite circles in both directions with the pelvis, hands on hips. | 60 seconds | 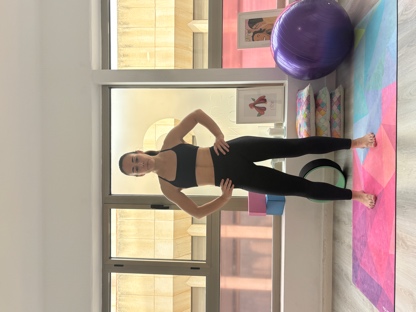 |
| **Pelvic floor contractions.** | Standing, neutral pelvis position, rapid pelvic floor contractions, then held while exhaling. Do not stay in apnoea. | Adapt the number of repetitions according to the assessment made in the  consultation. | 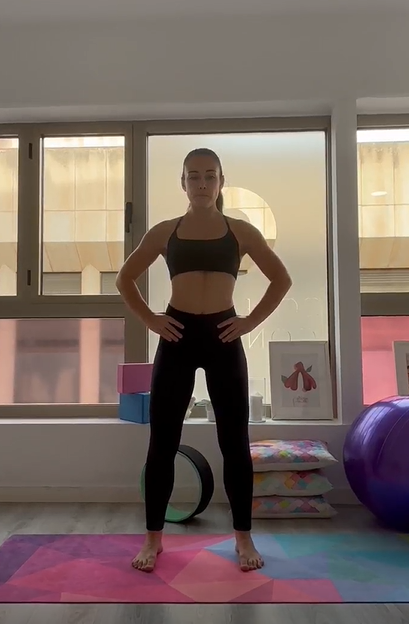 |
| **Balance and motor control on unstable bases.** | Balance and motor control exercises, with conscious breathing on trunk with stable base, on trunk with unstable base and on roller. | Hold each posture for 60 seconds. | 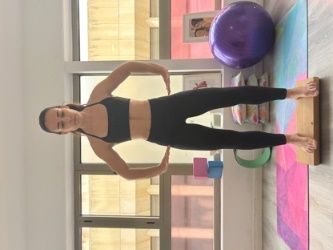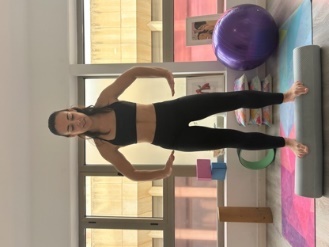 |
| **Deep squats.** | Feet placed wider than hip width apart. Deep squat and raise, activating transverse and pelvic floor. | 10 repetitions. | 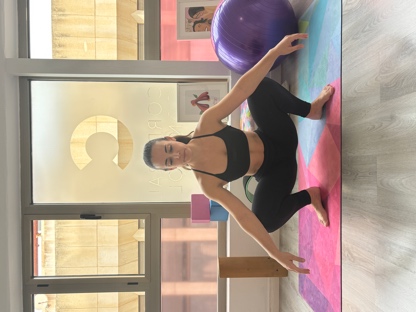 |
| **Isometric squats** | Feet placed wider than hip width apart. Squat held for 3 seconds and raise, all while activating the transverse and pelvic floor. | 10 repetitions. | 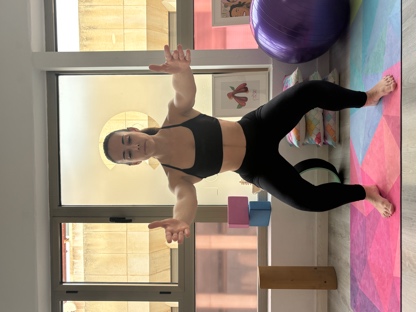 |
| **Alternating strides** | Standing, arms outstretched in front. Forward lunges  alternating legs. Activate transverse and pelvic floor. | 10 repetitions. | 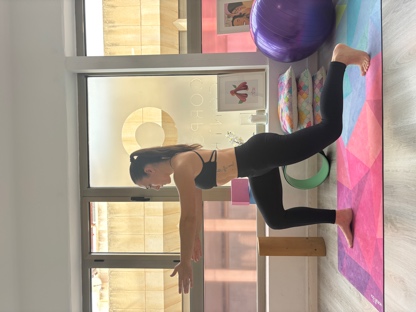 |
| **Self-stretching on the ground.** | Face up, legs flexed with heel support and dorsal flexion of the ankle. In resisted exhalation, self- elongation and activation of the transversus abdominis and pelvic floor. | 10 breaths. | 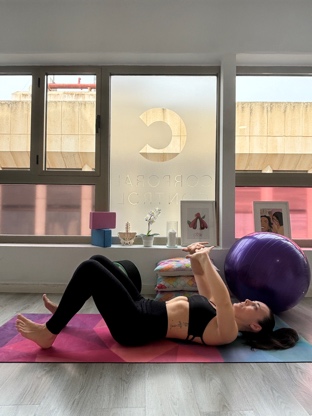 |
| **Gluteal bridge.** | Face up, legs flexed with heel support and dorsal flexion of the ankle. On exhalation resisted self- elongation and activation of the transversus abdominis and pelvic floor together with gluteal lift (gluteal  bridge). | 10 repetitions. | 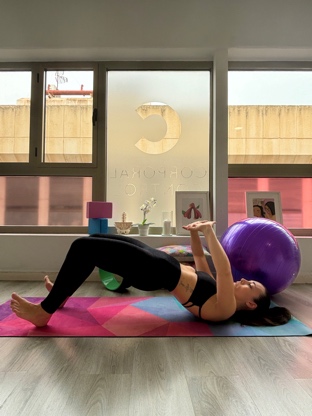 |
| **Gluteal bridge with alternate leg extension.** | Face up, legs bent with heel support. In resisted exhalation, self-elongation and activation of the transversus abdominis and pelvic floor and gluteus lift (gluteus bridge) with unilateral leg stretch. Same exercise with the other leg. | 10 repetitions. | 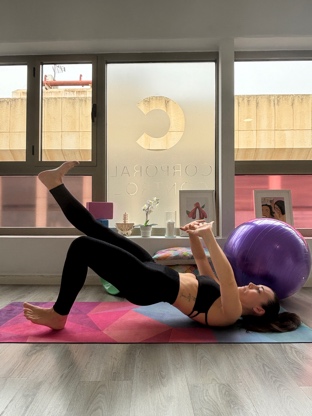 |
| **Activation of adductors with a ball.** | Face up, legs extended at 90° with a ball between them, hip and knee flexion and extension movement with adductor activation. Exercise with normalised breathing. | 10 repetitions. | 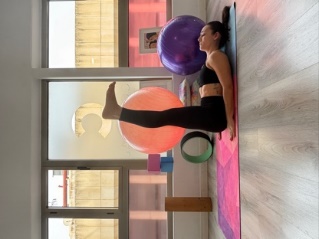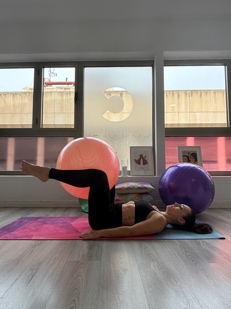 |
| **V-squat alternating bent- leg stretches with ball.** | Face up, legs at 90° with ball between them, hip and knee flexion-extension movement is performed with adductor activation. This is performed with exhalation on descent and  stretching of the legs. | 10 repetitions. | 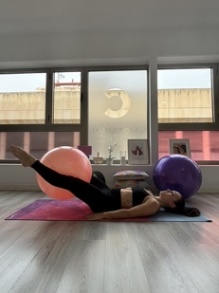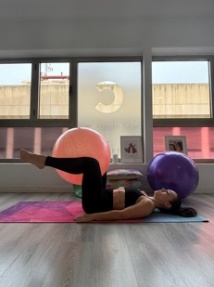 |
| **Active stretching and relaxation and body awareness session.** | Stretching arms, legs, neck, lower back, standing, sitting, calmly, paying attention to your breathing. Return to calm. | 5 minutes. | 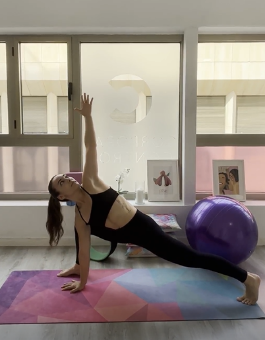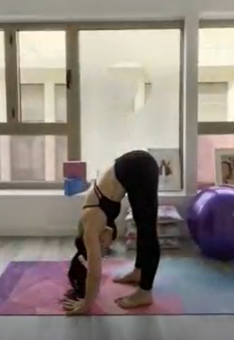  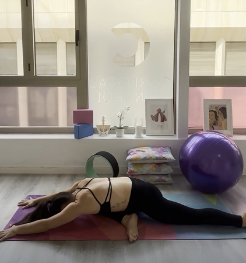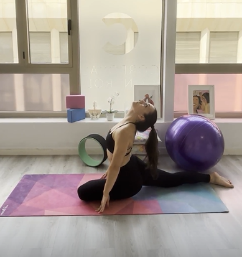 |
| **MONTH 2, Days 10, 12, 12, 14, 16** | | | |
| **Awareness and proprioception.** | Face up, awareness exercises and pelvic proprioception, performing sustained and rapid contractions. | According to initial patient assessment. | 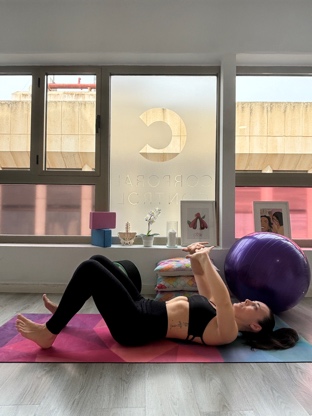 |
| **Self-elongation.** | Face up, legs flexed with heel support and dorsal flexion of the ankle. In resisted exhalation, self- elongation, growing and activation of the transversus abdominis and pelvic floor. | 10 breaths. | 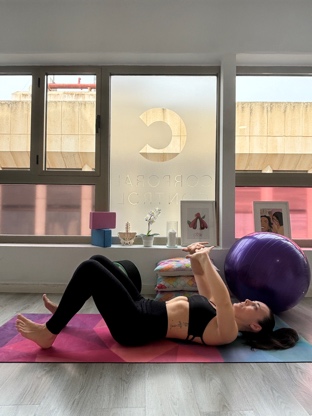 |
| **Gluteal bridge.** | Face up, legs flexed with support of heels and dorsal flexion of the ankle. In resisted exhalation, self- elongation and activation of the transversus abdominis  and pelvic floor together with gluteal elevation (gluteal bridge). | 10 repetitions. | 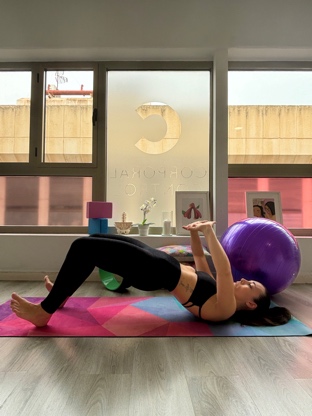 |
| **Gluteal bridge with alternate leg extension.** | Face up, legs bent with heel support. In resisted exhalation, self-elongation and activation of the transversus abdominis and pelvic floor and gluteus lift (gluteus bridge) with unilateral leg stretch. Same exercise with the other leg. | 10 repetitions. | 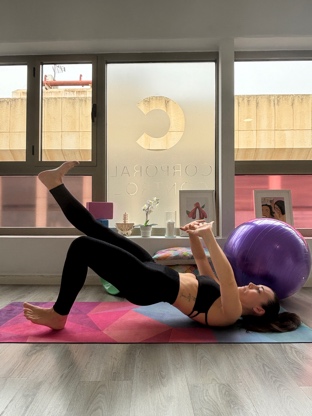 |
| **Leg raise lying on the side.** | On the side with legs straight, activation of the transverse and pelvic floor, perform lifting and lowering movement of the upper leg with maximum amplitude and slow speed and then with minimum amplitude of the movement and maximum speed. | 10 repetitions. | 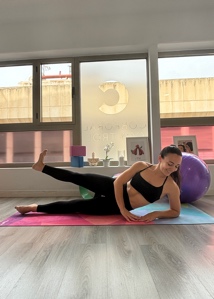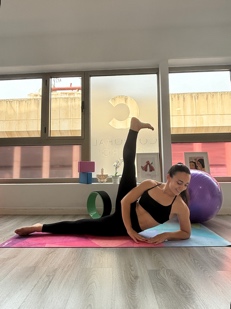 |
| **Circumduction of the leg sideways.** | On the side with legs stretched out, activation of the transversus abdominis and awareness of the pelvic floor, perform a circling movement with the upper leg in both directions. | 10 repetitions. | 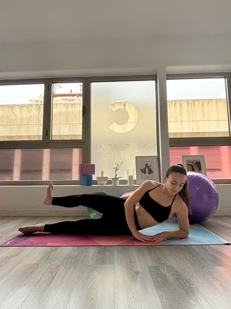 |
| **Side leg flexion and extension.** | On the side, legs aligned with the trunk, activate the transverse and pelvic floor, perform hip and knee flexion and extension movements. | 10 repetitions. | 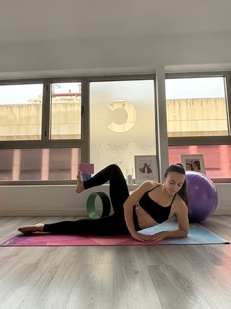 |
| **Hypopressives and self-stretching in inverted dog.** | On the quadruped perform hypopressive exercise (after 2 full breaths, go into apnoea and activate transverse and pelvic floor and hold for 10 seconds) with  push-up and leg stretching  (mountain or dog yoga position inverted). | 10 seconds. | 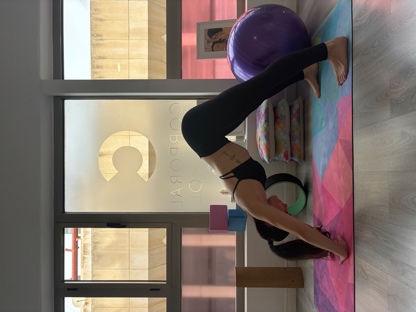 |
| **Hypopressives and self-stretching in inverted dog with alternating leg lifts.** | On the quadruped perform hypopressive exercise (after 2 full breaths, remain in apnoea and activate transversus abdominis and pelvic floor and hold for 10 seconds) with flexion of upper limbs and stretching of the lower limbs (mountain or dog yoga position Inverted) and add one leg lift to the ceiling and hip and knee flexion-extension movement. Repeat with the other leg. | 10 seconds. | 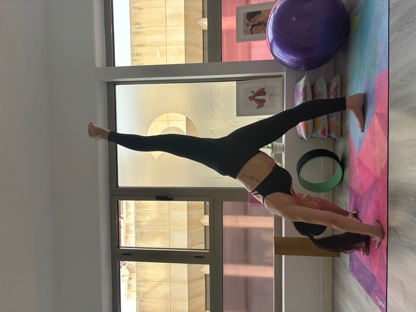 |
| **Front plank with arms bent.** | Front plank on toes and support on forearms. Back and legs aligned, abdominals strong. | 60 seconds. | 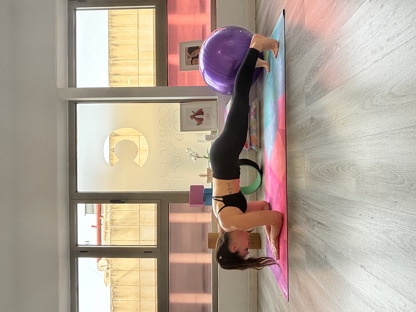 |
| **Front plank with arms outstretched.** | Front plank on toes and support on hands, arms extended. Back and legs aligned. Strong abdominals. | 60 seconds. | 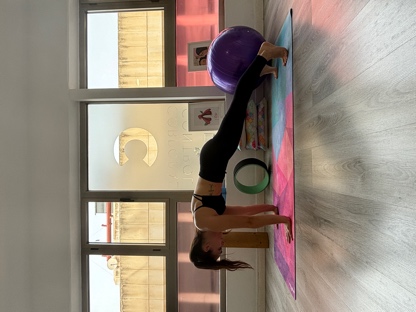 |
| **Side plates.** | Lateral plank, feet together, support on one arm stretched out and the other stretched up. Keep pelvis aligned with trunk. Repeat on the other side. | 60 seconds with  30 seconds rest between each. | 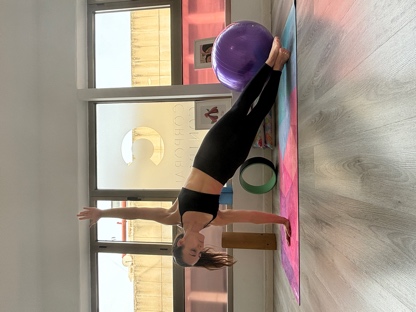 |
| **Front plank with alternating arm and leg raises.** | Front plate with alternating elevation of left arm and right leg and their opponents with strong abdominals and pelvic floor awareness. | 10 repetitions (5 on each side). | 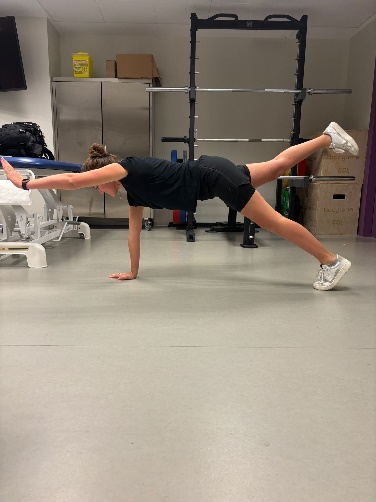 |
| **Front plank - climber.** | Front plank on outstretched arms. Bring the knee to the elbow on the same side, alternating legs. | 10 repetitions. | 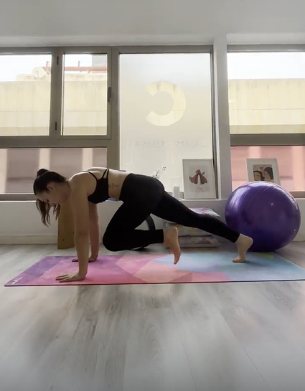 |
| **Exhalation crunch.** | Crunch on exhalation, activating abdominals, head lift with hands behind the head. | 10 repetitions. | 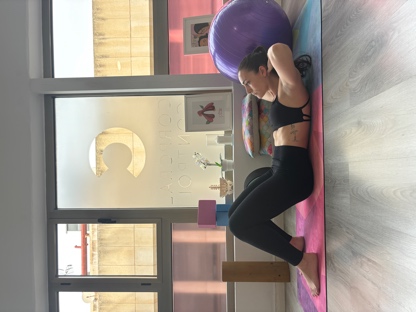 |
| **Exhalation crunch with lateral tilt.** | Exhalation crunch with lateral tilt. Hands crossed behind the head, elbow reaching for the opposite knee. | 10 repetitions on each side. | 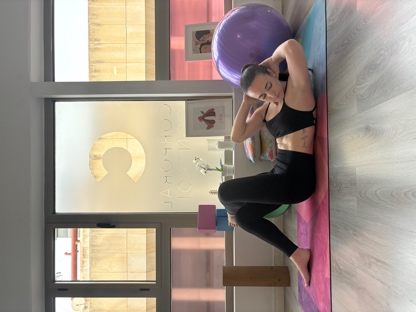 |
| **Crunch with trunk elevation and laterality.** | Legs bent without supporting heels. Trunk upright, contracting abdominals, trunk rolls to each side. | 10 repetitions on each side. | 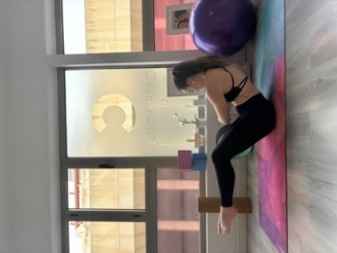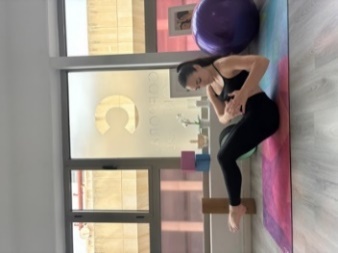 |
| **Oblique.** | Face up, legs bent, resting on heels. Slight elevation of the head and hands reaching for each heel alternately. | 10 repetitions on each side. | 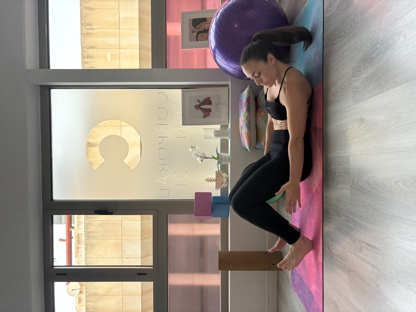 |
| **Active stretching**  **and relaxation**  **and body**  **awareness session.** | Stretching arms, legs, neck,  lower back, standing,  sitting, calmly, paying  attention to your breathing. | 5 minutes. | 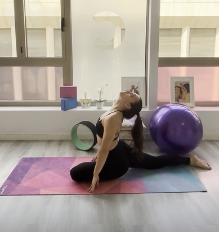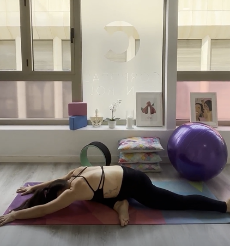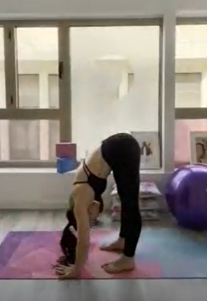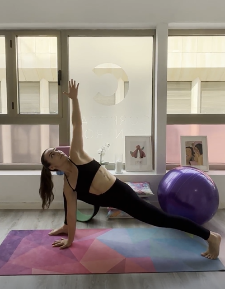 |

During this 3rd month, we will perform mobility, proprioception and pelvic floor muscle training exercises with increased loading through posture and movement. External load up to 60% of your RM will be introduced according to the progression of loads in terms of strength, endurance and health.

| **MONTH 3, Days 17, 19, 21, 23** | | | |
| --- | --- | --- | --- |
| **Gluteal bridge face** | Face up, legs flexed with heel support and dorsal flexion of the ankle. In resisted exhalation, self- elongation and activation of the transversus abdominis and pelvic floor together with gluteal elevation (gluteal bridge). | 10 repetitions. | 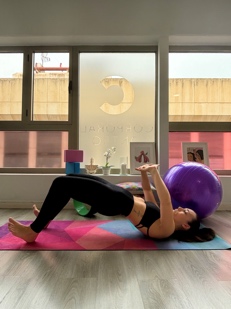 |
| **Gluteal bridge with alternating leg stretching on the back with alternating leg stretching** | Face up, legs bent with heel support. In resisted exhalation, self- elongation and activation of the transversus abdominis and pelvic floor and gluteus lift (gluteus bridge) with unilateral leg stretch. Same exercise with the other leg. | 10 repetitions. | 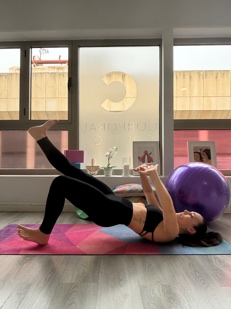 |
| **V-shape sit-ups alternating bent-leg stretches with a ball.** | Face up, legs at 90° with ball between them.  Perform hip and knee flexion-extension movement with adductor activation. This is performed with exhalation on the descent and stretching of the legs. | 10 repetitions. | 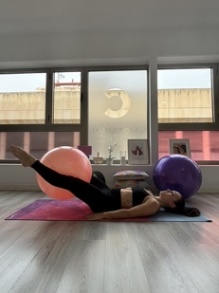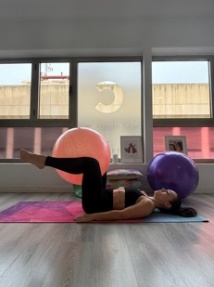 |
| **Front plank with arms bent.** | Front plank on toes and support on forearms.  Back and legs aligned, abdominals strong. | 90 seconds.  Rest: 30 sec | 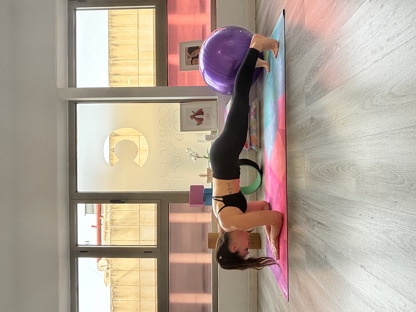 |
| **Front plank with arms outstretched.** | Front plank on toes and support on hands, arms extended. Back and legs aligned. Strong abdominals. | 90 seconds. Rest: 30sec. | 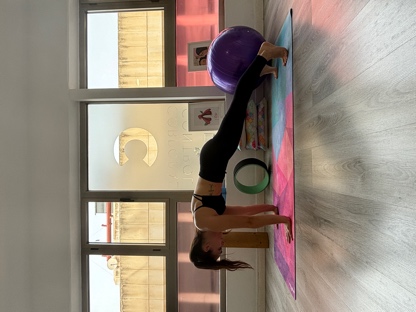 |
| **Lateral plank.** | Lateral plank, feet together, support on one arm stretched out and  the other arm stretched up. Repeat on the other side. | 90 seconds.  Rest: 30 | 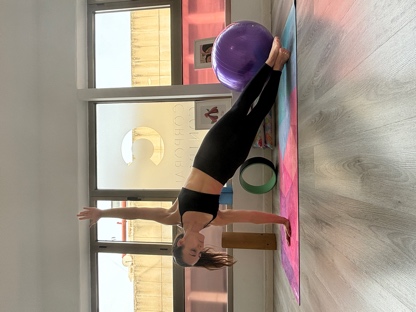 |
| **Front plank with alternating arm and leg raises.** | Front plate with alternating elevation of left arm and right leg and their opposites with strong abdominals and pelvic floor awareness.  Repeat with the other side. | 10 repetitions (5 on each side). | 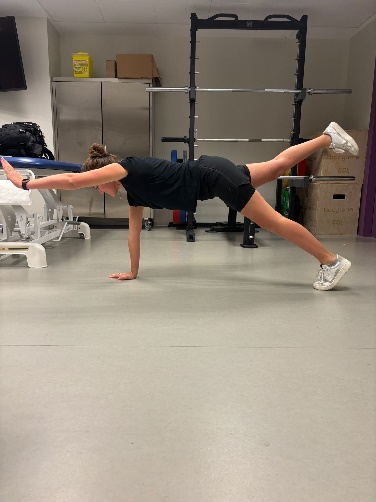 |
| **Front plank - climber.** | Front plank on outstretched arms. Bring the knee to the elbow on the same side,  alternating legs. | 10 repetitions. | 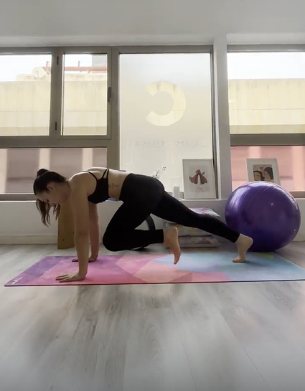 |
| **Active stretching and relaxation and body awareness session.** | Stretching arms, legs, neck, lower back, standing, sitting, calmly, paying attention to your breathing.  Return to calm. | 5 minutes. | 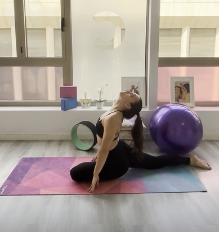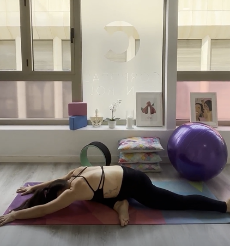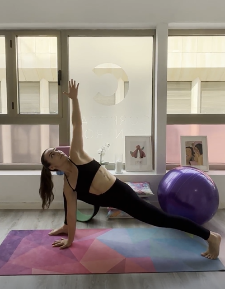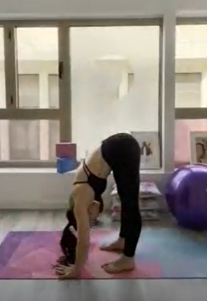 |
| **MONTH 3, Days 18, 20, 20, 22, 24** | | | |
| **Pelvic mobility.** | Standing, pelvic awareness exercise. Make infinite circles in both directions with the pelvis, hands on hips. | 60 seconds. | 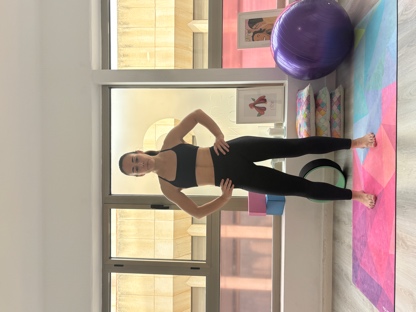 |
| **Pelvic floor contractions.** | Standing, neutral pelvis position, quick pelvic floor contractions, then  hold and exhale. Do not stay in apnoea. | Adapt the number of repetitions according to the  assessment made in the consultation. | 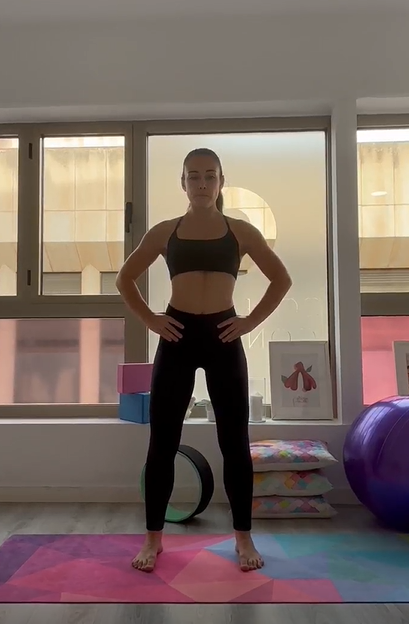 |
| **Balance and motor control on trunk with stable base.** | On the trunk, with the base stable on the floor, do ketellball push-ups and then rotate the ketellball around your abdomen. | 10 repetitions of each. | 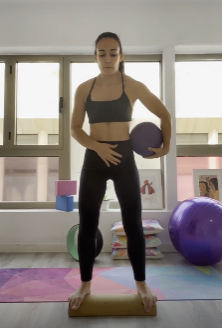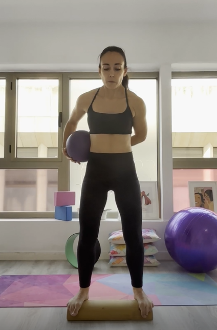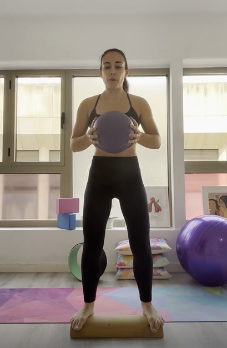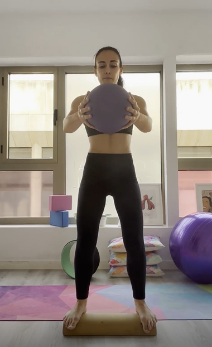 |
| **Balance and motor control on trunk**  **with unstable base.** | On the trunk, with the base unstable on the  floor, do ketellball push- ups and then rotate the ketellball around your abdomen. | 10 repetitions of each. | 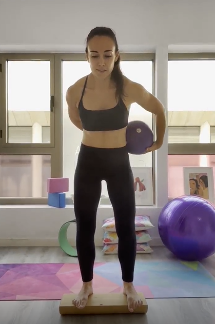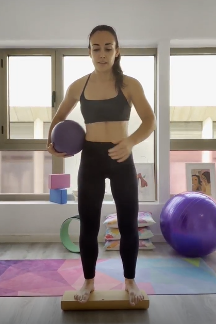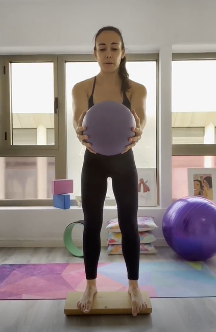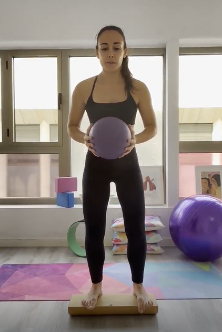 |
| **Balance and motor control on a roller.** | On the roller, do ketellball push-ups and then rotate the ketellball around your abdomen. | 10 repetitions of each. | 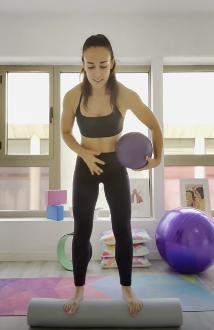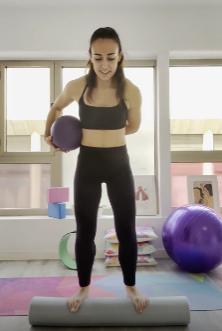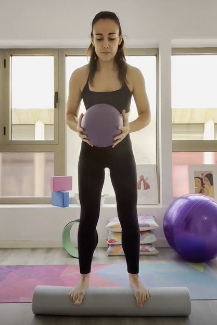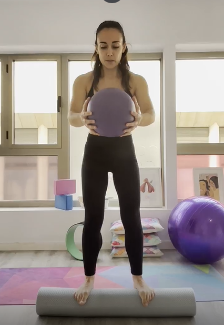 |
| **Deep squat with ketellball.** | Feet placed wider than hip width apart. Hold ketellball with arms extended in front. Deep squat and raise, activating transverse and pelvic floor. | 10 repetitions. | 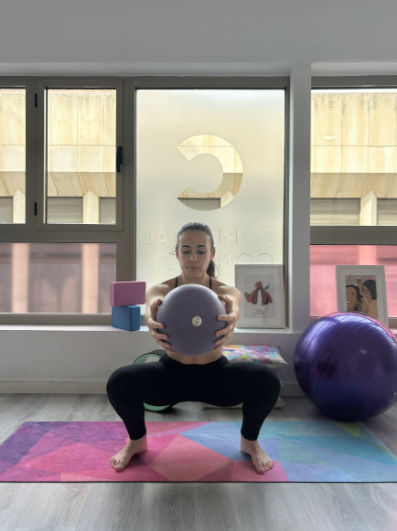 |
| **Isometric squat with ketellball.** | Feet placed wider than hip width apart. Hold ketellball with arms extended in front of you. Deep squat, hold for 3 seconds and raise, all activating transverse and pelvic floor. | 10 repetitions. | 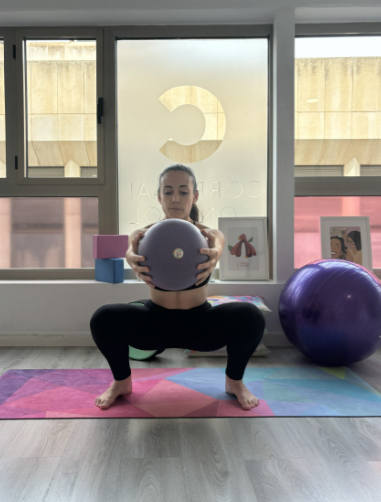 |
| **Alternating stride with** | Standing, catch ketellball with arms extended in front. Lunge forward alternating legs. Activating transverse  and pelvic floor. | 10 repetitions of each leg. | 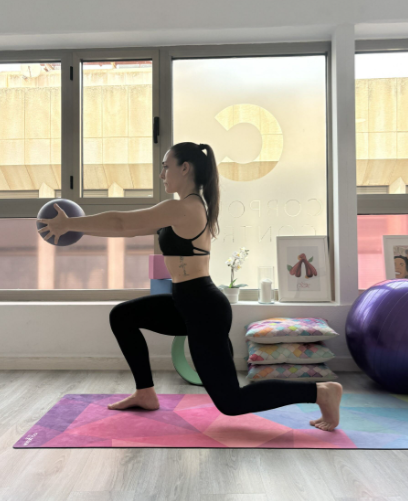 |
| **Hypopressives and self-stretching in inverted dog.** | On the quadruped perform hypopressive exercise  (after 2 full breaths, go into apnoea and activate transverse and pelvic floor and hold for 10 seconds) with flexion of upper limbs and stretching of the lower limbs (mountain or dog yoga position inverted). | 10 seconds. | 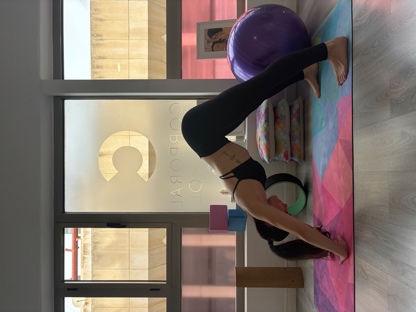 |
| **Hypopressives and self-stretching in inverted dog with alternating leg lifts.** | On the quadruped perform hypopressive exercise (after 2 full breaths, remain in apnoea and activate transversus abdominis and pelvic floor and hold for 10 seconds) with flexion of upper limbs and stretching of the lower limbs (mountain or dog yoga position  Inverted) and add one leg lift to the ceiling and hip and knee flexion- extension movement.  Repeat with the other leg. | 10 seconds with  each leg (20 seconds). | 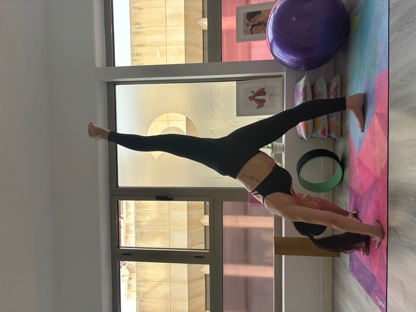 |
| **Crunch with ketellball.** | Lie on your back, support your heels, take the ketellball and raise the trunk a few degrees, activating the transverse and pelvic floor in expiratory time. | 10 repetitions. | 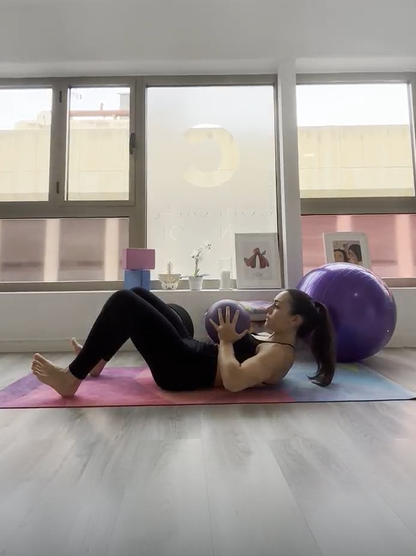 |
| **Crunch with side bends.** | Same exercise, but with 2 ketellballs (1 in each hand) or weights, and instead of raising the trunk, do inclines by bringing the ketellball closer to each heel. | 10 repetitions. | 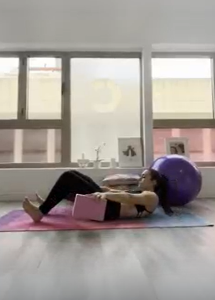 |
| **Crunch with trunk lift and laterality.** | Same position, but place the lateral side of the left foot on the opposite knee. Lifting the trunk with the ketellball, bring the right shoulder to the left knee. Repeat on the other side. | 10 repetitions. |  |
| **Oblique with lift and weight.** | Sitting, with the trunk leaning backwards and without supporting the legs, carry the ketellball  alternately to the left and right. | 10 repetitions on each side. |  |
| **Active stretching and relaxation and body awareness session.** | Stretching arms, legs, neck, lower back, standing, sitting, calmly, paying attention to your breathing. | 5 minutes |  |

During the 4th month, we will re-evaluate the RM for load management. We will increase it to 75% and focus on working and activating the CORE and pelvic floor in a dynamic way, with normalised breathing and introducing impact and fatigue, which are demonstrable risk factors in pelvic pathology, with the aim of automating the abdominopelvic synergy and its competence in everyday situations, as well as physical exercise.

| **MONTH 4, Days 25, 27, 29, 31** |
| --- |

| **Gluteal bridge with alternate leg**  **extension.** | Face up, legs bent with heel support. In resisted  exhalation, self-elongation and activation of the transversus abdominis and pelvic floor and gluteus lift (gluteus bridge) with unilateral leg stretch.  Same exercise with the other leg. | 10 repetitions. |  |
| --- | --- | --- | --- |
| **"Hip thrust with ketellball** | Face up, knees bent with heels on the floor, lifting toes to the ceiling, slowly release the air while contracting the abdomen and pelvic floor and using the ketellball to lift the  pelvis. | 10 repetitions. |  |
| **V-shape sit-ups alternating bent-leg stretches with a ball.** | Face up, legs at 90°° with a ball between them, hip and knee flexion-extension movement is performed with adductor activation. This is performed with exhalation in the descent and stretching of the legs. | 10 repetitions. |  |
| **Abdominals in hip and knee flexion and extension with ball.** | Face up, knees with hips at 90° and a ball between the knees squeezing to activate the inner thigh muscles, bring the feet towards the ceiling and lower the feet to the  floor without touching it. | 10 repetitions. |  |
| **Front plank with arms bent.** | Front plank on toes and support on forearms.  Back and legs aligned, abdominals strong. | 90 seconds. Rest : 30s |  |
| **Front plank with arms outstretched.** | Front plank on toes and support on hands, arms extended. Back and legs aligned. Strong abdominals. | 90 seconds. Rest : 30s |  |
| **Side planks** | Lateral plank, feet together, support on one arm stretched out and the other arm stretched up. Repeat on the other side. | 60 seconds with 30 seconds rest between each. |  |
| **Front plank with alternating arm and leg raises.** | Front plate with alternating elevation of left arm and right leg and their opposites with strong abdominals and pelvic floor awareness. Repeat with the other side. | 10 repetitions (5 on each side). |  |
| **Front plank - climber.** | Front plank on outstretched arms. Bring the knee to the elbow on the same side,  alternating legs. | 10 repetitions. |  |
| **Hypopressives and self-stretching in inverted dog with alternating leg lifts.** | On the quadruped perform hypopressive exercise  (after 2 full breaths, remain in apnoea and activate transversus abdominis and pelvic floor and hold for 10 seconds) with flexion of upper limbs and  stretching of the lower limbs (mountain or dog yoga position Inverted) and add one leg lift to the ceiling and hip and knee flexion- extension movement.  Repeat with the other leg. | 10 seconds. |  |
| **Active stretching and relaxation and body awareness session.** | Stretching arms, legs, neck, lower back, standing, sitting, calmly, paying attention to your breathing. Return to calm. | 5 minutes. |  |

| **MONTH 4, Days 26, 28, 30, 32** |
| --- |

| **Pelvis mobility and relaxation of the CORE and pelvic floor** | Standing, pelvic awareness exercise. Make infinite circles in both directions with the pelvis, hands on hips. | 60 seconds |  |
| --- | --- | --- | --- |
| **Ambulation Exercise with Sustained Contractions** | Walking at a comfortable pace while performing fast, sustained contractions, without going apnoeic. | Each person will do a specific number of repetitions according to their assessment. |  |
| **"Skeeping" with transverse activation and SP awareness** | Lift the knees alternately as if we were running in place, paying special attention to activating the CORE and maintaining awareness of the  pelvic floor. | Each person will do a specific number of repetitions according to their assessment. |  |
| **Balance and motor control exercises on trunk with** | Initially perform this exercise on a stable base, later on a trunk with an unstable base or on a roller. Bring the ketellball between the legs and push the hips forward and bring the ketellball above the chest, lower the ketellball in a controlled manner. | 10 repetitions. |  |
| **Isometric squat with ketellball.** | Feet placed wider than hip width apart. Hold ketellball with arms extended in front of you. Deep squat, hold for 3 seconds and raise, all activating transverse and pelvic floor. | 10 repetitions. |  |
| **Deep squat with ketellball** | Feet placed wider than hip width apart. Pick up ketellball with arms extended in front. Deep squat and pull up, all activating transverse  and pelvic floor. | 10 repetitions. |  |
| **Alternating stride with** | Standing, catch ketellball with arms extended in front. Lunge forward alternating legs.  Activating transverse and pelvic floor. | 10 repetitions of each leg. |  |
| **Burpees** | Standing, with feet shoulder width apart and arms at your sides, activate the transverse abdominis by drawing the navel in and up and contracting the pelvic floor muscles at the same time. From the standing position, lower into a squat position and place hands on the floor, jump up and bring feet into plank position, hold plank position and return to start. | 10 repetitions. |  |
| **Jump into the drawer** | Feet together, jump over a crate and jump down.  It can be done like climbing stairs. Raise awareness of pelvic  floor activation. | Repetitions: 10 |  |
| **Exhalation crunch with ketellball** | Crunch on exhalation, activating abdominals, head lift with hands  behind the head. | 10 repetitions. |  |
| **Crunch with side bends.** | Same exercise, but with 2 ketellballs (1 in each hand) or weights, slight flexion of the head, and instead of raising the trunk, do inclines bringing the  ketellball close to each heel. | 10 repetitions. |  |
| **Crunch with trunk lift and laterality.** | Same position, but place the lateral side of the left foot on the opposite knee.  Lifting the trunk with the ketellball, bring the right shoulder to the left knee. Repeat on the other side. | 10 repetitions. |  |
| **Oblique with lift and weight.** | Sitting, with the trunk leaning backwards and without supporting the legs, carry the ketellball alternately to the left and right. | 10 repetitions on each side. |  |
| **Active stretching and relaxation and body awareness session.** | Stretching arms, legs, neck, lower back, standing, sitting, calmly, paying attention to your breathing.  Return to calm. | 5 minutes. |  |

**ANNEX 5. MUSCLE STRENGTH RECORD SHEET**

Patient identification code:

| Manual assessment | | |
| --- | --- | --- |
| Balance Manual strength (oxford) | | |
|  | pre‐intervention | post‐intervention |
| 1 |  |  |
| 2 |  |  |
| 3 |  |  |
| Resistance  *"hold the contraction" count the seconds* | | |
|  | pre‐intervention | post‐intervention |
| 1 |  |  |
| 2 |  |  |
| 3 |  |  |
| Fatigue  *"contract as many times as possible".*  *6 sec contraction‐6s relaxation, no. of repetitions until unable to maintain contraction.* | | |
|  | pre‐intervention | post‐intervention |
| 1 |  |  |
| 2 |  |  |
| 3 |  |  |

| Phenix pelvimetre evaluation | | |
| --- | --- | --- |
| Maximum strength  *"contract as hard as you can for 10seconds" max peak* | | |
|  | pre‐intervention | post‐intervention |
| 1 |  |  |
| 2 |  |  |
| 3 |  |  |

**ANNEX 6. PELVIC FUNCTION AND QUALITY OF LIFE QUESTIONNAIRES**

‐ **PFIQ-20 (PELVIC FLOOR IMPACT QUESTIONNARIE Short Form)**

This questionnaire is about certain bowel, urinary or pelvic symptoms; you will be asked whether you feel these symptoms and, if so, how much they bother you. Please answer the questions below by placing an (X) in the appropriate box(es). If you are in doubt about any answer, please select the one that best suits your case. When answering this questionnaire, please take into account the symptoms you have felt in the last 3 months.

Please answer all questions in the following survey:

1. **Do you usually feel pressure in your lower abdomen?**

No ; Yes

If yes, how much does it bother you?

1 2 3 4

Nothing

A little Moderately A lot

1. **Do you usually have a feeling of heaviness in the pelvic area?**

No ; Yes

If yes, how much does it bother you?

1 2 3 4

Nothing

A little Moderately A lot

1. **Do you usually feel a "bump" or something sticking out that you can touch or see in the area of your vagina?**

No ; Yes

If yes, how much does it bother you?

1 2 3 4

Nothing

A little Moderately A lot

1. **Do you ever have to push on your vagina or around your rectum in to defecate or to finish a bowel movement?**

No ; Yes

If yes, how much does it bother you?

1 2 3 4

Nothing

A little Moderately A lot

1. **Do you often have a feeling of not completely emptying your bladder?**

No ; Yes

If yes, how much does it bother you?

1 2 3 4

Nothing A little Moderately A lot

1. **Do you ever have to push with your fingers on a "bulge" in the vaginal area to initiate or complete urination?**

No ; Yes

If yes, how much does it bother you?

1 2 3 4

Nothing

A little Moderately A lot

1. **Do you have the feeling that you have to push a lot in order to defecate?**

No ; Yes

If yes, how much does it bother you?

1 2 3 4

Nothing

A little Moderately A lot

1. **Do you have the feeling that you have not completely emptied your bowel after a bowel movement?**

No ; Yes

If yes, how much does it bother you?

1 2 3 4

Nothing

A little Moderately A lot

1. **Do you usually have involuntary faecal leakage when your stools are solid?**

No ; Yes

If yes, how much does it bother you?

1 2 3 4

Nothing

A little Moderately A lot

1. **Do you usually have involuntary faecal leakage when your stools are very soft or liquid?**

No ; Yes

If yes, how much does it bother you?

1 2 3 4

Nothing A little Moderately A lot

1. **Do you regularly have involuntary gas leakage (farting)?**

No ; Yes

If yes, how much does it bother you?

| 1 | 2 | 3 | 4 |
| --- | --- | --- | --- |
| Nothing | A little | Moderately | A lot |

1. **Do you usually feel pain when you defecate?**

No ; Yesi

If yes, how much does it bother you?

| 1 | 2 | 3 | 4 |
| --- | --- | --- | --- |
| Nothing | Un poco | Moderadamente | Mucho |

1. **Do you often have a strong sense of urgency so that you have to rush to the toilet to defecate?**

No ; Yes

If yes, how much does it bother you?

| 1 | 2 | 3 | 4 |
| --- | --- | --- | --- |
| Nothing | A little | Moderately | A lot |

1. **Does part of your bowel ever protrude from your anus while you are defecating or after you have just defecated?**

No ; Yes

If yes, how much does it bother you?

| 1 | 2 | 3 | 4 |
| --- | --- | --- | --- |
| Nothing | A little | Moderately | A lot |

1. **Do you usually urinate frequently?**

No ; Yes

If yes, how much does it bother you?

1 2 3 4

Nothing A little Moderately A lot

1. **Do you regularly experience involuntary urine leakage associated with a sense of urgency, i.e. a strong feeling of having to go to the toilet to urinate?**

No ; Yes

If yes, how much does it bother you?

1 2 3 4

Nothing A little Moderately A lot

1. **Do you regularly leak urine when coughing, sneezing or laughing?**

No ; Yes

If yes, how much does it bother you?

1 2 3 4

Nothing A little Moderately A lot

1. **Do you usually have the sensation of losing small amounts of urine (i.e. drops)?**

No ; Yes

If yes, how much does it bother you?

1 2 3 4

Nothing A little Moderately A lot

1. **Do you often have difficulty emptying your bladder?**

No ; Yes

If yes, how much does it bother you?

1 2 3 4

Nothing A little Moderately A lot

1. **Do you regularly experience pain or discomfort the lower abdomen or genital area?**

No ; Yes

If yes, how much does it bother you?

1 2 3 4

Nothing A little Moderately A lot

‐ **ICIQ-SF (International Consultation on Incontinence Questionnarie - Short Form)** There are many people who leak urine at any given time. We are trying to determine how many people have this problem and to what extent they are concerned about it. We would be very grateful if you could answer the following questions, thinking about how you

have been feeling in the LAST FOUR WEEKS.

1. How often do you leak urine (tick one)?

1 never

2 once a week or less

3 two or three times a week

- 1. once a day
  2. several times a day

6 continuously

1. We would like to know your impression of how much urine you think you leak. Amount of urine you usually leak (whether you are using protection or not) (Tick only one option)
2. nothing escapes me
3. too little quantity
4. a moderate amount

4 a lot.

1. How much do these urine leaks affect your daily life?

Please tick a number between 0 (I am not affected at all) and 10 (I am affected a lot) in the following list.

0 1 2 3 4 5 6 7 8 9 10

nothing much

ICI-Q score: add the scores of questions 3+4+5:

1. When do you leak urine (point out everything that happens to you)?
2. never leaks urine
3. leaks urine before reaching the WC

3 leaks urine when coughing or sneezing

1. loses when sleeping
2. leaks urine during physical exertion/exercise
3. leaks urine after urinating and is already dressed
4. leaks urine for no obvious reason
5. continuously leaks urine

‐ **Sandvick Severity Test**

This test assesses the severity of urinary incontinence symptoms in women.

1. How often do you leak urine (check one)?

1 Less than once a month

2 A few times a month

3 A few times a week 4 Every day and/or night

2.How much urine leaks each time (tick one).

1. Drops (very small amount)
2. Small squirt (a moderate amount)

3 A lot of quantity

**ANNEX 7. VAGINAL SYMPTOMS QUESTIONNAIRES**

‐ **VHI (Vaginal Health Index)**

**Mark the answer that indicates the status of the different vaginal aspects she presents:**

- 1. **Elasticity**
     1. **Absent**
     2. **Low**
     3. **Acceptabl**
     4. **Good**
     5. **Excellent**
  2. **Flow volume**
     1. **Absent**
     2. **Scarce**
     3. **Thin film on the surface**
     4. **Moderate layer**
     5. **Normal**
  3. **PH**
     1. **>6,1**
     2. **5,6 a 6**
     3. **5,5 a 5**
     4. **4,7 a 6**
     5. **<4,6**
  4. **Integrity of the epithelium**
     1. **Spontaneous petechiae**
     2. **Bleeds at the slightest touch**
     3. **Bleeds when scratched**
     4. **Non-friable epithelium**
     5. **Normal**
  5. **Humidity**
     1. **Absent. Swollen surface**
     2. **Absent. Normal surface**
     3. **Minima**
     4. **Moderate**
     5. **Normal**

**ANNEX 8. SEXUAL FUNCTION AND SELF-ESTEEM QUESTIONNAIRES**

‐ **FSFI (Female Sexual Function Index)**

The following questions are about your sexual feelings and responses during the last 4 weeks. Please answer the following questions as honestly and clearly as possible. Your answers will be kept completely confidential.

PLEASE TICK ONLY ONE ANSWER TO EACH QUESTION:

Sexual desire or interest: is the feeling that includes the desire to have a sexual experience, feeling receptive to a partner's sexual initiation, and thinking or fantasising about having sex.

1. **In the last 4 weeks, how often did you experience sexual desire or interest?**

Almost always or always

Most of the time (more than half of the time) Sometimes (about half the time)

Rarely (less than half of the time) Hardly ever or never

1. **In the last 4 weeks, how would you rate your level (degree) of sexual desire or interest?**

Very high Alto Moderate Under

Very low or not at all

Sexual arousal: is the sensation that includes physical and mental aspects of sexual arousal. It may include sensations of heat or throbbing in the genitals, lubrication (wetness) or muscle contractions.

1. **In the last 4 weeks, how often did you feel sexual arousal during sexual activity or vaginal intercourse?**

No sexual activity Almost always or always

Most of the time (more than half of the time) Sometimes (about half the time)

Rarely (less than half of the time) Hardly ever or never

1. **In the last 4 weeks, how would you rate your level of sexual arousal during sexual activity or vaginal intercourse?**

No sexual activity Very high

Alto Moderate Under

Very low or not at all

1. **In the last 4 weeks, how confident were you in becoming aroused during sexual activity or vaginal intercourse?**

No sexual activity Very high confidence High confidence Moderate confidence Low confidence

Very low or no confidence

1. **In the last 4 weeks, how often were you satisfied with your arousal during sexual activity or vaginal intercourse?**

No sexual activity Almost always or always

Most of the time (more than half of the time) Sometimes (about half the time)

Rarely (less than half of the time) Hardly ever or never

1. **In the last 4 weeks, how often did you get vaginal lubrication (vaginal wetness) during sexual activity or vaginal intercourse?**

No sexual activity Almost always or always

Most of the time (more than half of the time) Sometimes (about half the time)

Rarely (less than half of the time) Hardly ever or never

1. **In the last 4 weeks, how much difficulty did you have in getting lubricated (vaginal wetness) during sexual activity or vaginal intercourse?**

No sexual activity

Extremely difficult or impossible Very difficult

Difficult

A bit difficult No difficulty

1. **In the last 4 weeks, how often did you maintain vaginal lubrication (vaginal wetness) until the end of sexual activity or vaginal intercourse?**

No sexual activity Almost always or always

Most of the time (more than half of the time) Sometimes (about half the time)

Rarely (less than half of the time) Hardly ever or never

1. **In the last 4 weeks, how much difficulty did you have in maintaining vaginal lubrication (vaginal wetness) until the end of sexual activity or vaginal intercourse?**

No sexual activity

Extremely difficult or impossible Very difficult

Difficult

A bit difficult No difficulty

1. **In the last 4 weeks, when you had sexual stimulation or vaginal intercourse, how often did you achieve orgasm (climax)?**

No sexual activity Almost always or always

Most of the time (more than half of the time) Sometimes (about half the time)

Rarely (less than half of the time) Hardly ever or never

1. **In the last 4 weeks, when you had sexual stimulation or vaginal intercourse, how difficult was it for you to reach orgasm (climax)?**

No sexual activity

Extremely difficult or impossible Very difficult

Difficult

A bit difficult No difficulty

1. **In the last 4 weeks, how satisfied were you with your ability to reach orgasm (climax) during sexual activity or vaginal intercourse?**

No sexual activity Very satisfied Moderately satisfied

Neither satisfied nor dissatisfied Moderately dissatisfied

Very dissatisfied

1. **In the last 4 weeks, how satisfied were you with the emotional closeness with your partner during sexual activity?**

No sexual activity Very satisfied Moderately satisfied

Neither satisfied nor dissatisfied Moderately dissatisfied

Very dissatisfied

1. **In the last 4 weeks, how satisfied were you with your sexual relationship with your partner?**

Very satisfied Moderately satisfied

Neither satisfied nor dissatisfied Moderately dissatisfied

Very dissatisfied

1. **In the last 4 weeks, how satisfied were you with your overall sex life?**

Very satisfied Moderately satisfied

Neither satisfied nor dissatisfied Moderately dissatisfied

Very dissatisfied

1. **In the last 4 weeks, how often did you experience discomfort or pain during vaginal intercourse?**

Without vaginal intercourse Almost always or always

Most of the time (more than half of the time) Sometimes (about half the time)

Rarely (less than half of the time) Hardly ever or never

1. **In the last 4 weeks, how often did you experience discomfort or pain after vaginal intercourse?**

Without vaginal intercourse Almost always or always

Most of the time (more than half of the time) Sometimes (about half the time)

Rarely (less than half of the time) Hardly ever or never

1. **In the last 4 weeks, how would you rate your level (degree) of pain or discomfort during or after the last 4 weeks?**

Without vaginal intercourse

Veri high

High

Moderate

Under

Very low or not at all

‐ **VAS (Visual Analogue Scale)**

Mark on the line the point that indicates the intensity of pain during sexual intercourse. The rating will be: 1 Mild pain if the patient scores the pain as less than 3; 2 Moderate pain if the rating is between 4 and 7; 3 Severe pain if the rating is 8 or more.

0 1 2 3 4 5 6 7 8 9 10

‐ **S-BIS (Body Image Scale)**

In this questionnaire you will be asked about how you feel about your physical appearance and any changes that may have occurred as a result of your illness or treatment.

Please read each item carefully and mark the answer that best fits how you have been feeling during the past week.

1. **Have you ever felt embarrassed or self-conscious about your physical appearance?**
   1. **Not at all**
   2. **A little**
   3. **Quite**
   4. **A lot**

**Have you felt physically less attractive as a result of the disease or its treatment?**

1. **Not at all**
2. **A little**
3. **Quite**
4. **A lot**
5. **Have you ever felt unhappy with the way you look when you are dressed?**
   1. **Not at all**
   2. **A little**
   3. **Quite**
   4. **A lot**
6. **Have you felt less feminine/masculine as a result of your illness or treatment?**
   1. **Not at all**
   2. **A little**
   3. **Quite**
   4. **A lot**
7. **Do you find it difficult to look at yourself when you are naked?**
   1. **Not at all**
   2. **A little**
   3. **Quite**
   4. **A lot**
8. **Have you felt less sexually attractive as a result of your illness or treatment?**
9. **Not at all**
10. **A little**
11. **Quite**
12. **A lot**
13. **Have you avoided someone because of the way you felt about their appearance?**
    1. **Not at all**
    2. **A little**
    3. **Quite**
    4. **A lot**
14. **Have you felt that the treatment has left your body "less complete"?**
    1. **Not at all**
    2. **A little**
    3. **Quite**
    4. **A lot**
15. **Have you ever felt dissatisfied with your body?**
    1. **Not at all**
    2. **A little**
    3. **Quite**
    4. **A lot**
16. **Have you been dissatisfied with the appearance of your scar?**
17. **Not at all**
18. **A little**
19. **Quite**
20. **A lot**

‐ **Treatment Satisfaction Scale**

How satisfied are you with the treatment you received?

- 1. Totally dissatisfied
  2. Dissatisfied
  3. Somewhat dissatisfied
  4. Satisfied

Fully satisfied
