# Supplementary material for: Effectiveness of radiofrequency and exercise-based rehabilitation on symptoms associated with pelvic floor dysfunction in breast cancer patients: A study protocol
Source: PLoS One. 2025 Aug 29;20(8):e0330156. doi: 10.1371/journal.pone.0330156 (PMC12396660; doi:10.1371/journal.pone.0330156)
Supplement: S4 File — (DOCX) [file pone.0330156.s004.docx]

**Memoria solicitud comité de ética para la investigación biomédica**

**Título:**

Efectividad de la radiofrecuencia y la rehabilitación basada en el ejercicio físico sobre la sintomatología asociada a las disfunciones del suelo pélvico en pacientes con cáncer de mama. Ensayo clínico aleatorizado.

**Nombre de IP proyecto de investigación:**

Cristina Orts Ruiz

**EQUIPO INVESTIGADOR:**

Dra. Da. Cristina Salar Andreu

Dr. D. Sergio Montero Navarro

Dra. Da. Sonia del Río Medina

Dr. D. José M. Botella-Rico

Dra. Da. María Torres Lacomba

Dr. D. Josep C. Benítez Martínez

Dr. D. Jesús Sánchez Más

**RESUMEN**

El cáncer de mama tiene un elevado impacto, afectando a 2,3 millones de mujeres en el mundo en 2022. En España, en 2023, hubo 40.203 nuevos casos. Además de los costos económicos y sociales, las pacientes presentan disfunciones genitourinarias debido a los tratamientos oncológicos. El Síndrome Genitourinario de la Menopausia (SGU) afecta al 50% de las mujeres menopáusicas, siendo mayor la prevalencia en mujeres con cáncer de mama.

Este estudio tiene como objetivo comparar la efectividad de la radiofrecuencia (RF) y el ejercicio de la musculatura del suelo pélvico (EMSP) en el tratamiento de las disfunciones pélvicas asociadas al SGU en pacientes con cáncer de mama. Se realizará un ensayo clínico aleatorizado, doble ciego, en colaboración con la Asociación de Mujeres Afectadas por el Cáncer de Mama de Elche y Comarca y la Universidad CEU Cardenal Herrera. Participarán mujeres diagnosticadas con cáncer de mama y disfunción pélvica, divididas en tres grupos: uno recibirá terapia basada en EMSP, otro en RF y otro EMSP+RF.

Se espera que ambas técnicas mejoren la calidad de vida de las pacientes, donde la RF podría ofrecer beneficios adicionales debido a su efectividad demostrada en el tratamiento de la sequedad vaginal y la dispareunia. Los resultados esperados contribuirán a establecer protocolos de tratamiento más efectivos y menos invasivos. El proyecto presentado tiene el potencial de impactar positivamente en la salud y bienestar de las mujeres con cáncer de mama, reduciendo la sintomatología asociada a la enfermedad y su tratamiento, y mejorando su calidad de vida.

Palabras clave

Cáncer de mama, síndrome genitourinario, radiofrecuencia, ejercicio físico

## **Introducción**

## **Cáncer de mama: incidencia e impacto económico.**

El cáncer de mama es, sin duda, una enfermedad de gran impacto a nivel mundial, con importantes implicaciones tanto en términos de salud como económicas y sociales. Así, en 2022, cerca de 2.3 millones de mujeres en todo el mundo fueron diagnosticadas con cáncer de mama, de las cuales 666103 fallecieron a causa de esta enfermedad^1^. En el año 2023, se registraron 40203 nuevos casos de cáncer de mama en España y 6759 fallecieron^2^. Estos datos indican que, aunque la tasa de supervivencia comenzó a mejorar en la década de 1990, gracias a la implementación de programas de detección temprana del cáncer y a tratamientos integrales que incluían medicamentos eficaces en diversos países^3^, es necesario realizar un esfuerzo aún mayor para mejorar la detección precoz y la eficiencia de los tratamientos que redunde en datos más alentadores.

Se calcula que el cáncer de mama puede llegar a tener un coste total de hasta 42000 euros, entre gastos directos, ocultos y pérdidas de ingresos por bajas, despidos y reducciones de horario. Concretamente, el 96 % de los pacientes ha gastado una media de 3590 euros en gastos médicos como servicios de nutrición, pruebas y consultas en la sanidad privada, servicios de psicología o rehabilitación^4^. Además, el cáncer de mama no solo afecta a las mujeres individualmente, sino que también tiene un impacto en el equilibrio familiar, la sociedad y el sistema de salud^5^, por lo que el impacto negativo socio-económico es aún mayor y se escapa de una estimación real.

### **Etiología y grupos de riesgo**

El cáncer de mama es una enfermedad caracterizada por el crecimiento descontrolado de células mamarias alteradas, que pueden formar tumores.

Este tipo de cáncer generalmente se origina en los conductos galactóforos o en los lobulillos que producen leche en la mama. En su etapa inicial, conocida como estadio 0 o in situ, el cáncer no representa una amenaza mortal. Sin embargo, a medida que las células cancerosas se propagan al tejido mamario circundante, pueden formar nódulos o causar engrosamiento^3^. Los cánceres invasivos tienen el potencial de diseminarse a los ganglios linfáticos cercanos u otros órganos, lo que se conoce como metástasis. Estas metástasis pueden ser fatales si no se tratan adecuadamente.

Algunas mutaciones genéticas hereditarias, como las mutaciones en los genes BRCA1, BRCA2 y PALB2, aumentan significativamente el riesgo de padecer cáncer de mama. Las mujeres con estas mutaciones pueden considerar estrategias para reducir el riesgo, como la mastectomía preventiva de ambos senos^3,6^.

Ser mujer es el factor de riesgo principal para desarrollar cáncer de mama. Otros factores que incrementan la probabilidad de padecer esta enfermedad son el envejecimiento, la obesidad, el consumo excesivo de alcohol, antecedentes familiares de cáncer de mama, historial de exposición a radiación, historial reproductivo (como la edad de la menarquia y el primer embarazo), consumo de tabaco y tratamiento hormonal posmenopáusico. Aproximadamente la mitad de los casos de cáncer de mama se dan en mujeres que no presentan ningún factor de riesgo identificable, a excepción del género (femenino) y la edad (más de 40 años)^3,6^. Los antecedentes familiares aumentan el riesgo, aunque la mayoría de las mujeres diagnosticadas no los tienen por lo que la ausencia de antecedentes familiares no implica necesariamente un menor riesgo de padecer la enfermedad^3^.

### **Síntomas**

Los síntomas del cáncer de mama suelen no manifestarse en sus etapas iniciales, por lo que su detección suele ocurrir mediante programas de detección, como la mamografía de rutina, o como resultado del seguimiento de otras afecciones. En los casos sintomáticos, es común la presencia de una masa en la mama, que suele ser firme, con bordes irregulares y no dolorosa. Sin embargo, también pueden presentarse otros síntomas como descamación de la areola y la piel, formación de costras, enrojecimiento, hinchazón de la mama, hoyuelos en la piel, dolor en el pecho, dolor óseo, úlceras en la piel, ganglios linfáticos inflamados, pérdida de peso y secreción de sangre^7^.

### **Tratamiento**

El enfoque del tratamiento varía según las características individuales de la paciente, el subtipo de la enfermedad y su extensión ya sea limitada al seno y los ganglios linfáticos (estadios II o III) o se haya propagado a otras partes del cuerpo (estadio IV).

En el ámbito médico se emplean una combinación de tratamientos para disminuir las posibilidades de recurrencia del cáncer, tales como:

• Cirugía para extirpar el tumor del seno.

• Radioterapia para reducir el riesgo de recurrencia en los tejidos mamarios y regiones circundantes.

• Tratamientos farmacológicos dirigidos a eliminar las células cancerosas y prevenir su propagación, que pueden incluir terapias hormonales, quimioterapia o tratamientos específicos con productos biológicos.

La intervención quirúrgica puede consistir en la extirpación del tejido afectado por el cáncer (tumorectomía o mastectomía parcial) o la remoción completa del seno (mastectomía total). En la cirugía de cánceres invasivos, se realiza la extirpación de los ganglios linfáticos. En el pasado, se consideraba indispensable una extirpación completa del lecho de los ganglios linfáticos axilares (llamada disección axilar completa) para prevenir la propagación del cáncer. Sin embargo, en la actualidad, se opta por realizar una intervención menos invasiva conocida como "biopsia del ganglio centinela", ya que conlleva menos complicaciones^3,8^.

La elección de los fármacos para tratar el cáncer de mama se basa en las características biológicas específicas del cáncer, que se identifican mediante pruebas especializadas, como la determinación de marcadores tumorales. La mayoría de los medicamentos empleados en el tratamiento del cáncer ya están incluidos en la Lista Modelo de Medicamentos Esenciales de la Organización Mundial de la Salud^9^.

Los tratamientos farmacológicos (quimioterapia) contra el cáncer de mama pueden ser administrados previamente (neoadyuvantes) o posteriormente (adyuvantes) de la cirugía, y se determinan según la subtipificación biológica del cáncer. Aquellos cánceres que expresan receptores de estrógeno (RE) o receptores de progesterona (RP) suelen responder favorablemente a tratamientos endocrinos, como el tamoxifeno o los inhibidores de la aromatasa. Estos medicamentos se administran por vía oral durante un periodo de tiempo comprendido entre 5 a 10 años y reducen significativamente las posibilidades de recurrencia en cánceres con receptores hormonales positivos, aunque pueden causar síntomas de la menopausia^3^.

Por otro lado, los cánceres que no expresan RE ni RP, llamados negativos para receptores hormonales, se tratan con quimioterapia, a menos que el tumor de un tamaño muy reducido. Las pautas de quimioterapia actuales son altamente efectivas para reducir las posibilidades de propagación o recurrencia del cáncer y generalmente se administran de forma ambulatoria. En condiciones normales, la quimioterapia para el cáncer de mama no requiere hospitalización^3^.

Algunos cánceres de mama pueden sobre expresar una molécula llamada oncogén HER2/neu de forma independiente. Este tipo de cáncer puede tratarse con fármacos biológicos dirigidos (inmunoterapia), como el trastuzumab, que son altamente efectivos pero costosos, ya que son anticuerpos y no productos químicos. Estos tratamientos biológicos se combinan con quimioterapia para aumentar su eficacia en la eliminación de las células cancerosas^3^.

La radioterapia se emplea para tratar los posibles tumores microscópicos remanentes en el tejido mamario o los ganglios linfáticos, con el fin de disminuir las probabilidades de una recurrencia que pueda afectar la pared torácica^3^.

La radioterapia juega un papel fundamental en el tratamiento del cáncer de mama. En etapas tempranas, puede evitar la necesidad de una mastectomía. En etapas más avanzadas, incluso después de una mastectomía, puede disminuir el riesgo de recurrencia. En casos avanzados, en ciertas situaciones, la radioterapia puede reducir la probabilidad de mortalidad debido a la enfermedad^3^.

## **Síndrome genitourinario en cáncer de mama**

Las mujeres diagnosticadas con cáncer de mama experimentan una afectación emocional significativa debido a las secuelas físicas, psicológicas y sociales, que incluyen mastectomía, linfedema, menopausia temprana, infertilidad, miedo a la recurrencia, estrés emocional y cambios en las relaciones familiares y laborales^10^.

Entre los efectos secundarios de estos tratamientos se encuentran disfunciones en la vida sexual de las mujeres^11^, esterilidad, anovulación, amenorrea, atrofia vaginal, sofocos y menopausia precoz^12^. Los síntomas de la menopausia afectan diversas áreas de la vida de las mujeres, disminuyendo su calidad de vida^13^.

El síndrome genitourinario de la menopausia (SGU) es frecuente en el 50% de las mujeres menopáusicas, pero su incidencia es mayor en mujeres con cáncer de mama, principalmente debido a la cirugía, quimioterapia, radiación y terapia hormonal^14^. El SGU es un conjunto de síntomas y signos provocados principalmente por la disminución de estrógenos y que provoca cambios tanto en la vagina y los genitales externos como en la uretra y en la vejiga^15^. El SGU se caracteriza por sequedad vaginal, ardor e irritación vulvar y vaginal, falta de lubricación, dispareunia, disuria, urgencia urinaria e infecciones recurrentes del tracto urinario^14^, así como disfunciones del suelo pélvico y del tracto intestinal tales como estreñimiento y diarrea^16^.

Sousa *et al.*^17^ señalaron que el 59% de las mujeres con cáncer de mama encuestadas en su estudio experimentaron problemas genitourinarios, siendo la incontinencia urinaria de esfuerzo (IUE) y la urgencia miccional los más comunes. Además, muchas de ellas reportaron mayor frecuencia urinaria, nicturia, infecciones recurrentes del tracto urinario, prolapso, ardor, malestar/dolor, vejiga hiperactiva y dispareunia, empeorando con la terapia endocrina complementaria^17^.

En otro estudio, Córdoba de Juan *et al*.^18^ observaron que el 40,2% de las mujeres con cáncer de mama encuestadas presentaban síntomas de disfunción del suelo pélvico. Sin embargo, otros autores indican tasas más bajas de disfunciones del suelo pélvico en mujeres sobrevivientes de cáncer de mama en comparación con un grupo control, a pesar de que estas mujeres han sido sometidas a un mayor número de intervenciones quirúrgicas relacionadas con el suelo pélvico debido al cáncer de mama^19^.

### **Impacto en la función pélvica**

La incontinencia urinaria (IU) es la disfunción genitourinaria más común entre las mujeres con cáncer de mama, seguida de la incontinencia fecal (IF) y, en último lugar, el prolapso^20^. Tanto Stahlschmidt *et al*.^21^ como Córdoba-de Juan *et al*.^18^ observaron que entre el 40% y el 47% de las mujeres encuestadas presentaron IU, mientras que un 7,3% presentaba incontinencia urinaria de urgencia (IUU), un 25% presentaba IUE y otro 25% vejiga hiperactiva (VH). No se encontraron diferencias significativas entre las mujeres que recibían inhibidores de aromatasa y aquellas que consumían tamoxifeno. Además, concluyeron que las pacientes de mayor edad y con una adherencia inferior al tratamiento tenían una mayor incidencia de IU^21^.

Por otro lado, tras cinco años de tratamiento con tamoxifeno, el 93,33% de las mujeres encuestadas presentaba IU, siendo el 21,43% IUU, el 71,43% IUE y el 7,14% de tipo mixta (IUM), con una frecuencia cuatro veces mayor en mujeres que habían tenido hijos^22^. Sin embargo, debido al tamaño muestral, no se pudo establecer una asociación con la edad ni con las terapias adyuvantes. Este porcentaje se redujo al 38% después de 10 años desde el diagnóstico de cáncer de mama, sin diferencias entre aquellas que tomaban inhibidores de aromatasa y las que no^20^. Por lo tanto, Robinson *et al*.^20^ no pudieron confirmar que los niveles más altos de testosterona producidos por estos medicamentos protegieran contra la IU. En cuanto a la IF, un estudio no detectó IF entre las mujeres con cáncer de mama, pero sí un 6,1% de incontinencia de gases^18^. Sin embargo, el porcentaje de mujeres que la padecían después de 10 años desde el diagnóstico era del 17,6%, siendo más común en aquellas que tomaban inhibidores de aromatasa (29,8%) en comparación con las que no lo hacían (16,4%)^20^. Además, se estableció que la edad estaba asociada con la IF, pero no se encontró una relación directa entre el tamoxifeno, el índice de masa corporal ni la cirugía pélvica con esta disfunción.

Algunos autores señalaron que entre el 7,3% y el 8,1% de las mujeres encuestadas presentaban prolapso, sin diferencias significativas entre aquellas que consumían inhibidores de aromatasa y las que no^18,20^. Por todo esto, aún resulta necesario realizar más estudios que generen conocimiento con el objetivo de comprender la asociación que existe entre el tipo de tratamiento recibido y las disfunciones pélvicas en pacientes diagnosticadas de cáncer de mama.

### **Impacto en la función sexual**

Según Oberguggenberger *et al*.^23^, el 68,8% de las mujeres con cáncer de mama experimentaron problemas en el deseo o interés sexual, en comparación con el 58,8% del grupo control. Además, el deseo sexual fue el aspecto más afectado según el cuestionario Índice de la Función Sexual Femenina (FSFI), tanto inmediatamente después del tratamiento como seis meses después, principalmente en mujeres mayores de 55 años, posmenopáusicas, casadas y con hijos.

Se encontraron diferencias significativas en el deseo sexual relacionadas con el tipo de cirugía (mastectomía radical, preservación de piel y preservación de pezón), el método de extracción (linfadenectomía axilar o ganglio centinela) y el estado menopáusico de la mujer. Específicamente, en mujeres sometidas a mastectomía, la disminución del deseo sexual aumentó tanto a los seis como a los doce meses después del diagnóstico^23^.

En un estudio de Robinson *et al*.^20^ realizado en mujeres diagnosticadas de cáncer de mama, diez años después del diagnóstico, no se encontraron diferencias en el deseo sexual hipoactivo entre mujeres sexualmente inactivas que estaban tomando tamoxifeno en ese momento y las que no lo estaban. Sin embargo, hubo variaciones dependiendo del consumo de inhibidores de aromatasa, siendo menor el deseo sexual en aquellas que los consumían.

Por otro lado, un 17,3% de las mujeres encuestadas expresaron falta de interés en tener relaciones sexuales, principalmente debido a síntomas menopáusicos como la sequedad vaginal y sentimientos de insatisfacción con su cuerpo en las últimas cuatro semanas. Sin embargo, se observó que había más problemas sexuales después del tratamiento (72,09%) en comparación con el momento del diagnóstico (51,35%) y doce meses después de este (59,26%).

Según el estudio de Oberguggenberger *et al*.^23^.El interés y el deseo sexual se correlacionaron positivamente con la satisfacción con la pareja y diferentes dimensiones de calidad de vida, mientras que se relacionaron negativamente con la depresión.

Más del 50% de las mujeres con cáncer de mama experimentaron molestias durante las relaciones sexuales, lo que se asoció con la sequedad vaginal común en este grupo. Además, se observó que el tiempo era un factor significativo en la dispareunia, ya que las mujeres reportaron mayor dolor justo después del tratamiento (41,864%) y seis meses después (40,74%) en comparación con el momento del diagnóstico (32,43%). Esto se atribuyó como consecuencia de la quimioterapia^18^.

Las mujeres diagnosticadas con cáncer de mama experimentaban más incomodidad durante las relaciones sexuales en comparación con las mujeres sin esta enfermedad, y los investigadores sugirieron que esto podría estar relacionado con los problemas de feminidad informados por las propias pacientes^23^. En otro estudio realizado por Robinson *et al*.^20^, se examinó cómo la toma de inhibidores de aromatasa afectaba a esta variable. Después de 10 años desde el diagnóstico, se observó que las mujeres que tomaban estos inhibidores presentaban una mayor angustia sexual (70%) en comparación con aquellas que no los tomaban (52,7%). Los autores asociaron este hallazgo con la atrofia vulvovaginal secundaria a este tratamiento^20^.

## **Tratamiento para el SGU**

Existen distintas opciones de tratamiento para la sintomatología asociada al SGU, que van desde enfoques conservadores hasta opciones más invasivas como la cirugía, dependiendo de la gravedad de los síntomas a tratar.

Las mujeres que han superado el cáncer de mama experimentan síntomas más intensos del SGU en comparación con aquellas que están en la etapa posmenopáusica y no han enfrentado la enfermedad.

Aunque la terapia hormonal es ampliamente reconocida como el tratamiento más respaldado para el SGU, hay restricciones y desafíos asociados con su uso. El estrógeno está contraindicado en individuos con historial de cáncer de mama, cáncer de endometrio, trombosis venosa profunda, embolia pulmonar, enfermedad hepática y sangrado vaginal inexplicable. Por lo tanto, no se puede utilizar terapia hormonal, ya sea sistémica o local, debido a que la terapia con estrógenos combinada con progesterona aumenta el riesgo de accidente cerebrovascular, embolia pulmonar y trombosis venosa profunda, aunque no de enfermedad coronaria^24^.

### **Tratamiento no conservador**

Entre las opciones más invasivas para abordar el SGU se encuentra la cirugía destinada a corregir la IUE. Esta intervención tiene como objetivo fortalecer los ligamentos pubouretrales y el tejido conectivo parauretral en la uretra media^25^.

Por lo general, el tratamiento quirúrgico se clasifica en procedimientos abdominales (abiertos o laparoscópicos), procedimientos vaginales y agentes de carga uretral. Los procedimientos abdominales incluyen la técnica de Marshall Marchetti Krantz (MMK), la colposuspensión de Burch y el cabestrillo pubovaginal^25,26^. Por otro lado, los procedimientos vaginales comprenden el procedimiento de Pereyra modificado (PPM) y las Bandas suburetrales libres de tensión (BLT), que pueden ser retropúbicas (TVT) o transobturadoras (TOT)^26,27^. Las BLT, generalmente hechas de polipropileno, se colocan vaginalmente para pasar la malla por el agujero obturador y debajo de la uretra media con el menor daño posible a los tejidos circundantes. Además, se introdujo la técnica de bandas de incisión única, fijadas en el músculo obturador o en su fascia mediante una pequeña incisión vaginal, asociada a menos dolor, pero con efectos adversos similares en comparación con las BLT convencionales^25^.

La técnica de aumento de volumen uretral implica la inyección de materiales sintéticos, como el colágeno, en la capa de mucosa uretral para proporcionar soporte y estrechar la abertura del cuello de la vejiga. Este procedimiento se lleva a cabo en un consultorio bajo anestesia local. A menudo se requieren de dos a tres inyecciones para lograr una mejora significativa en los síntomas^28^.

Las complicaciones más comunes posteriores al tratamiento quirúrgico son dificultad en la micción, infección del tracto urinario, dispareunia postoperatoria, erosión de la malla, persistencia o recurrencia de la IU o prolapso de órganos pélvicos, lesión del tracto genitourinario (incluyendo perforación de la vejiga) y lesión del tracto gastrointestinal^29,30^. Se ha observado que las complicaciones a corto plazo asociadas con la técnica de TVT alcanzan el 19%, e incluyen retención urinaria, infección de la herida y laceración de la vejiga durante la cirugía. Otras posibles complicaciones de la cirugía de cabestrillo TVT abarcan la perforación iatrogénica del tracto urinario inferior o del intestino, hemorragia significativa, exposición de la malla, erosión (tanto de la uretra como de la vejiga), infecciones del implante, aparición de disfunción miccional (como urgencia urinaria u obstrucción de la salida de la vejiga), retención e infecciones urinarias. Aunque muchas de estas complicaciones son a corto plazo y pueden resolverse, otras, como la erosión y la perforación de órganos, pueden resultar más difíciles de tratar y a menudo requieren cirugías adicionales con mayor riesgo de complicaciones^31^.

Para los síntomas menopáusicos después del cáncer de mama, independientemente del tipo de cáncer de mama (hormonal, Her2 o triple negativo), el tratamiento hormonal sistémico sigue contraindicado. Esto es válido para el estrógeno, la progesterona, la dehidroepiandrosterona (DHEA) pero también para los tratamientos con testosterona que se prueban regularmente para mejorar la función sexual en mujeres en los Estados Unidos. Sin embargo, aún no está aprobado debido a la eficacia limitada y la falta de datos sobre la seguridad a largo plazo. La optimización del estilo de vida y el ejercicio físico regular son las terapias más eficaces para aliviar todos los síntomas menopáusicos^32^.

El ospemifeno oral está aprobado para su uso en el tratamiento de la dispareunia debida a la menopausia. La dosificación oral una vez al día mejora los síntomas vulvovaginales de manera similar a la terapia con estrógenos locales, pero no está aprobada para su uso en el cáncer de mama y puede aumentar los riesgos de tromboembolismo venos^33^.

Entre las opciones farmacológicas aprobadas se encuentra la oxibutinina que es un anticolinérgico que bloquea el receptor muscarínico en el músculo liso de la vejiga e inhibe las contracciones del detrusor. Los anticolinérgicos suelen ocasionar sequedad bucal y estreñimiento como efectos secundarios predominantes. Además, existe la posibilidad de que agraven las arritmias cardíacas preexistentes y empeoren el glaucoma de ángulo estrecho^34^. En el tratamiento no hormonal, los inhibidores selectivos de la recaptación de serotonina (ISRS), los inhibidores de la recaptación de serotonina y norepinefrina (IRSN) y la gabapentina pueden usarse para tratar síntomas vasomotores de la menopausia. Los ISRS y los IRSN, como la paroxetina, el escitalopram y la venlafaxina, son antidepresivos que tratan los síntomas vasomotores. La paroxetina, en particular, es el único fármaco aprobado por la FDA (Administración de Alimentos y Medicamentos de Estados Unidos) para esta indicación y los síntomas disminuyen una semana después de iniciar el tratamiento. Sin embargo, la paroxetina y la fluoxetina no deben usarse simultáneamente con tamoxifeno debido a los efectos inhibidores sobre la enzima CYP2D6^35,36^.

### **Tratamiento conservador**

El enfoque conservador abarca diversas intervenciones dirigidas al estilo de vida de la paciente, la normalización de la musculatura del suelo pélvico, así como otras estrategias como la reeducación vesical, la electroestimulación, la radiofrecuencia o el uso de geles hidratantes y lubricantes. Las modificaciones del estilo de vida y el ejercicio físico regular se han sugerido como las terapias más eficaces para mitigar todos los síntomas menopáusicos. Estos tratamientos, en su mayoría, no son invasivos, con escasos o nulos efectos secundarios y no obstaculizan posibles tratamientos futuros si fueran necesarios. Asimismo, su coste suele ser asequible, en cualquier caso, mucho menor al coste farmacológico o quirúrgico. Por todo ello, el tratamiento conservador debe ser considerado como la primera línea de manejo del SGU.

La fisioterapia es fundamental en el tratamiento de las disfunciones relacionadas con el suelo pélvico. Implica una amplia variedad de técnicas terapéuticas entre las que propone la implementación de ejercicio físico terapéutico y técnicas de movilización articular para mejorar la condición muscular y respiratoria.

Dentro del abordaje con ejercicio físico terapéutico se encuentra la normalización del CORE y el entrenamiento de la musculatura de suelo pélvico (EMSP). La estabilización del CORE es un enfoque importante en fisioterapia, que sugiere que la disfunción lumbopélvica puede originarse por un desequilibrio entre las estructuras de soporte más grandes y las más pequeñas y débiles, como el suelo pélvico, el transverso del abdomen y el diafragma respiratorio. El objetivo de este enfoque es restaurar el equilibrio y la fuerza en la región central del cuerpo. Las posturas anormales de la pelvis pueden provocar aumento de la tensión en las articulaciones y una activación muscular excesiva. Por lo tanto, una postura incorrecta puede desencadenar la aparición de puntos gatillo, hipertonicidad y dolor pélvico. Es esencial que la fisioterapia diseñe un plan de tratamiento integral que aborde estas irregularidades musculoesqueléticas. Este plan debe incluir ejercicios de fortalecimiento muscular, estiramientos, técnicas posturales, tratamiento de la diástasis abdominal y reeducación del suelo pélvico. El EMSP implica la renovación activa del control neuromuscular en los músculos de esta área. Este proceso puede abarcar el fortalecimiento de músculos debilitados, la disminución de la tensión muscular y la recoordinación del control muscular global^37^.

Los mecanismos por los cuales el ejercicio terapéutico y el EMSP ejercen sus efectos beneficiosos en pacientes con cáncer de mama aún no se comprenden completamente. Sin embargo, se postula que estas intervenciones pueden actuar a través de diversas vías, incluyendo el fortalecimiento de la musculatura del suelo pélvico, la mejora de la propiocepción, disminución de la sintomatología abdomino-pélvica y mejora de la calidad de vida^38-40^.

En su ámbito de competencia, el fisioterapeuta puede trabajar directamente con la musculatura del suelo pélvico, proporcionando educación y entrenamiento para mejorar el control motor en esta área. El fisioterapeuta empleará técnicas que promuevan la relajación y contracción activa del suelo pélvico. Esta respuesta aprendida de relajar y contraer el suelo pélvico puede ser cultivada durante la terapia física mediante indicaciones verbales, terapia manual y biorretroalimentación^37^.

En un estudio realizado en 2019 por R. Schvartzman *et al*.^41^, se exploró la hipótesis de que la fisioterapia del suelo pélvico podría mejorar el dolor, la función sexual y la calidad de vida en mujeres climatéricas que padecen dispareunia. Los hallazgos de este estudio ayudan a determinar el papel crucial de la fisioterapia en estas condiciones. Según el artículo citado, se concluyó que la intervención de fisioterapia en mujeres con dispareunia climatérica resultó en mejoras significativas en el dolor, la calidad de vida, la función sexual y la musculatura del suelo pélvico^41^.

La rehabilitación del suelo pélvico en mujeres postmenopáusicas, con IU, mejora los síntomas y signos de la disfunción pélvica asociada al SGU, además de mostrar un impacto positivo en las actividades de la vida diaria, la calidad de vida y la función sexual^42-44^.

La radiofrecuencia (RF) es otra técnica conservadora que ha ganado reconocimiento debido a su practicidad y viabilidad como terapia segura y efectiva para tratar los síntomas vaginales y la disfunción sexual. Esta técnica utiliza ondas electromagnéticas de alta frecuencia para generar micropuntos térmicos en la dermis superficial y profunda, con una acción térmica limitada en cuanto a lateralidad y profundidad. Este enfoque permite una regeneración adecuada sin dejar marcas cicatriciales ni secuelas, y estimula la reparación tisular al promover la producción y reorganización de colágeno, fibras elásticas y vascularización, mejorando así el trofismo. En ginecología, se utiliza terapéuticamente para mejorar la función en diversas áreas, incluyendo las paredes vaginales, el vestíbulo vulvar, el meato uretral, los labios menores y mayores, el perineo y la región perianal. El primer estudio piloto sobre RF vaginal (Linly™ Loktal Medical Electronics) investigó la respuesta clínica de 14 pacientes con síntomas de SGU después de someterse a RF en la vagina y el introito vaginal. "La radiofrecuencia demostró ser efectiva en el tratamiento de la sequedad vaginal y la dispareunia, eliminando la necesidad de lubricante vaginal durante el periodo de observación y mejorando el tropismo vaginal”^45^. Un estudio posterior examinó el efecto de la RF en la salud vaginal, la microbiota y la celularidad en mujeres posmenopáusicas, demostrando que la RF es efectiva y segura para restablecer el equilibrio vaginal, promoviendo la predominancia de especies de Lactobacillus y un pH ácido en el fluido vaginal, lo que podría proteger a estas mujeres de infecciones vaginales, inflamación e infecciones del tracto urogenital^46^.

Un ensayo clínico aleatorizado evaluó el efecto de la RF para la IUE, comparando con el EMSP y la combinación de ambas terapias (RF + EMSP) en 117 mujeres climatéricas con IUE. Tras el tratamiento, se observó una mejora significativa en las puntuaciones urinarias en los tres grupos, con un beneficio mayor en el grupo RF + EMSP. Los síntomas y la laxitud vaginal mejoraron con la RF y el índice de salud vaginal fue más alto en los grupos RF y RF + EMSP. Además, la función sexual mejoró en los grupos RF y EMSP. La combinación de RF y EMSP demostró una mejora significativa en los síntomas de IUE y la sequedad vaginal, mientras que la laxitud vaginal mejoró de manera similar. Sin embargo, la combinación de RF y EMSP en la función sexual no mostró beneficios superiores a los logrados con las terapias individuales^47^.

Otro estudio demostró que la RF, además de ser un tratamiento eficaz y seguro para el liquen escleroso vulvar, provoca un aumento significativo en la concentración vulvar de colágeno tipo III en relación con el colágeno tipo I después del tratamiento, lo que se asoció con una mejoría notable de los síntomas y de la elasticidad^48^. En este sentido, en 2010, Millheiser *et al*.^49^ demostraron su eficacia en el tratamiento del síndrome de laxitud vaginal postparto. La evidencia sugiere utilidad y eficacia en el tratamiento de la vaginitis atrófica, la disfunción orgásmica y la IUE^49,50^.

Actualmente ningún estudio ha evaluado la efectividad de la RF como tratamiento de los síntomas asociados a la disfunción pélvica asociada al SGU característico en pacientes con cáncer de mama. Debido a la seguridad y eficacia demostrada de la RF como terapia conservadora de la disfunción pélvica, resulta necesario evaluar su acción terapéutica en pacientes con cáncer de mama, y compararlo con el tratamiento conservador de referencia como es el EMSP, para conocer si pudiera ser un tratamiento coadyuvante.

Hipótesis

El tratamiento conservador basado en RF debería ser efectivo en la mejora de los síntomas asociados a la aparición de SGU y alteración de la función pélvica en mujeres diagnosticadas de cáncer de mama, pudiendo ser una terapia coadyuvante al EMSP.

# **Objetivos**

El objetivo del estudio es comparar la efectividad del tratamiento basado en RF con el tratamiento basado en el EMSP sobre la disfunción pélvica derivada del SGU presente en pacientes con cáncer de mama.

Objetivos específicos

Evaluar el efecto de la RF, el EMSP y de la combinación de ambas terapias en la IU, IF y prolapso en mujeres diagnosticadas de cáncer de mama.

Evaluar el efecto de la terapia de RF y EMSP en la calidad de vida de las mujeres diagnosticadas de cáncer de mama

Analizar el efecto de la RF y EMSP en la función sexual de las mujeres diagnosticadas de cáncer de mama.

Conocer el impacto de terapia de la RF y EMSP en la percepción de la autoimagen corporal en las mujeres diagnosticadas de cáncer de mama

1. **Metodología y plan de trabajo**

   2. **Diseño del estudio**

Se realizará un ensayo clínico aleatorizado, doble-ciego, entre mujeres diagnosticadas con cáncer de mama con disfunción pélvica que serán sometidas a terapia basada en EMSP y RF, sola o combinada. El estudio se realizará en las instalaciones de la Asociación de Mujeres Afectadas por el Cáncer de Mama de Elche y Comarca (AMACMEC), con domicilio social en Calle Olegario Domarco Seller, 93. Entresuelo de Elche (Alicante), y provista del CIF G53270146. Dicha asociación, que cuenta actualmente con un total de 812 asociadas, tiene actualmente un convenio de colaboración firmado con la Universidad CEU Cardenal Herrera y ha firmado la autorización para poder desarrollar este estudio en concreto. El estudio comenzará en septiembre de 2024, con una estimación de duración de dos años como se detalla en un cronograma más adelante. AMACMEC cuenta con una sala de fisioterapia que garantiza la privacidad e idoneidad para llevar a cabo las evaluaciones y tratamiento con RF, así como una sala de ejercicio físico en la que se realizarán los grupos basados en EMSP.

El estudio se realizará bajo las indicaciones de la Declaración de Helsinki y según la legislación española vigente (Real Decreto 223/2004 y Ley de Investigación Biomédica de 2007). Se garantizará el cegamiento de los resultados registrados ya que no se recogerán datos personales que permitan la identificación del sujeto ni datos referidos a cuentas de correo electrónico. Los datos se almacenarán en la nube de OneDrive asociada a la FUNDACIÓN UNIVERSITARIA SAN PABLO CEU (en adelante, FUSP-CEU), a la que únicamente se le concederá acceso a los investigadores responsables del análisis, extracción y manejo de los mismos, a través de una clave de acceso (Jesús Sánchez Más y Cristina Orts). Los datos estarán identificados mediante un código, de manera que no incluya información que pueda identificar al paciente. Los datos serán tratados de acuerdo con lo establecido por el Reglamento (UE) 2016/679 del Parlamento Europeo y del Consejo, de 27 de abril de 2016, relativo a la protección de las personas físicas en lo que respecta al tratamiento de datos personales y a la libre circulación de estos datos y por el que se deroga la Directiva 95/46/CE. Los datos serán tratados bajo la responsabilidad de la FUSP-CEU, con la finalidad de gestionar su participación en el presente proyecto de investigación. Puede contactar con el Delegado de Protección de Datos de FUSP-CEU, dirigiendo su petición por escrito a la dirección postal C/ Tutor nº 35 - 28008 Madrid o a la dirección de correo electrónico dpd@ceu.es. Se solicitará aprobación al Comité de Ética para la Investigación Biomédica de la Universidad CEU Cardenal Herrera. Una vez obtenida la aprobación por parte del Comité, el estudio se registrará en clinicaltrials.gov.

- 1. **Participantes**

Se invitará a participar voluntariamente en el estudio a mujeres diagnosticadas con cáncer de mama procedentes de AMACMEC. Para la captación de participantes se realizarán charlas informativas grupales. Posteriormente se ampliará la información referida al proyecto mediante entrevista personal a realizar el día de la valoración de la participante para su inclusión al estudio.

Los criterios de inclusión son: mujeres mayores de edad con historial clínico de cáncer de mama, que acepten participar en el estudio y que presenten una disfunción pélvica evaluada mediante el cuestionario *Pelvic Floor Distress Inventory*^51^ (PFDI20) ≥ 100, criterio establecido mediante resultados previos obtenidos por el grupo investigador que caracterizaron la sintomatología relacionada con el suelo pélvico en 250 mujeres diagnosticadas de cáncer de mama y asociadas de AMACMEC (proyecto “Disfunciones abdominopélvicas en mujeres afectadas por cáncer de mama, IDOC22-07, datos no publicados).

Los criterios de exclusión son: haber realizado EMSP o haber recibido RF en los últimos 12 meses, uso de estrógenos vaginales en los últimos 6 meses, terapia hormonal sistémica en los últimos 6 meses, terapia con láser en los últimos 6 meses, ausencia de contracción del suelo pélvico según la Escala Modificada de Oxford, uso de marcapasos, corazón descompensado o enfermedades metabólicas, déficit cognitivo, trastornos neurológicos periféricos o centrales, cirugías previas en la región pélvica, patologías cutáneas o heridas en la zona de tratamiento o presencia de una infección activa del tracto urinario y/o vagina.

- 1. **Cálculo de la muestra**

El tamaño de la muestra se calculó según estudios previos que demostraron diferencia estadísticamente significativa en la sintomatología del suelo pélvico y la calidad de vida relacionada con el suelo pélvico después del tratamiento con RF^52^. Considerando una pérdida del 30% durante el seguimiento en la muestra, con un riesgo alfa (α) del 5% y una potencia (1-β) del 80%, se calcula un tamaño muestral de 117 mujeres (39 en cada grupo).

- 1. **Diseño del estudio**

El primer contacto con la paciente susceptible de participar en el proyecto lo realizará siempre la misma investigadora principal (IP) que les informará debidamente del estudio y entregará la hoja de información a los participantes (ANEXO 1) y el consentimiento informado (ANEXO 2). Una vez aceptada la participación en el estudio, se le entregará el cuestionario PFDI20 que permitirá conocer la disfunción pélvica de la paciente y su idoneidad a la hora de ser incluida en el estudio. Seguidamente se les proporcionará un cuestionario creado *ad hoc* (ANEXO 3) que permita conocer las características sociodemográficas, así como la información relacionada con el diagnóstico, el tratamiento del cáncer de mama, así como otros cuestionarios específicos validados para valorar la función pélvica, los síntomas vaginales, la función sexual y la percepción de la imagen corporal, que se detallarán más adelante.

A continuación, siempre realizado por una fisioterapeuta especializada en el área de suelo pélvico, se medirá la fuerza de la MSP en las mujeres.

Otro investigador cegado para las determinaciones anteriores realizará la asignación de forma aleatoria a los grupos de intervención. Las participantes serán asignadas a uno de los tres grupos de intervención: terapia de RF no ablativa fraccionada, terapia de EMSP y terapias combinadas de RF y EMSP (RF + EMSP). La asignación aleatorizada se realizará mediante el programa SAS 9.4 (SAS Institute, Cary, NC, EE. UU.) con una asignación 1:1:1. La información sobre el tratamiento a utilizar se guardará dentro de un sobre opaco cerrado identificado por un número.

Una fisioterapeuta realizará la intervención basada en RF, otra fisioterapeuta realizará la intervención basada en EMSP. Otro fisioterapeuta, que desconocerá el grupo de intervención, realizará las determinaciones posteriores al tratamiento. No será posible cegar a los participantes respecto al tratamiento.

- 1. **Tratamiento basado en radiofrecuencia**

El proceso terapéutico propuesto consistirá en aplicar RF en la modalidad de transferencia eléctrica capacitiva (dispositivo Capernergy® modelo C500 Urogyne) Se trata de un dispositivo diseñado principalmente para el abordaje de disfunciones del área uroginecológica, donde el aumento de la temperatura de los tejidos es regulado por un sensor de temperatura, con 3 frecuencias (0,8MHz, 1MHz y 1,2 MHz) que permitirán abordar distintas profundidades de tejido, y una potencia de 310w. Este dispositivo consta de dos electrodos: uno activo capacitivo que se colocará en la zona vaginal con un cubresondas y gel hidrosoluble y otro electrodo dispersivo o placa de retorno que se posicionará en la región lumbosacra.

El protocolo a realizar está basado en el tratamiento descrito por Pinheiro *et al*.^53^ para el tratamiento del SGU postmenopausia, en el que las participantes serán colocadas en posición litotómica. La temperatura de tratamiento se establecerá en 41ºC, con una frecuencia de 1Mhz y potencia de 75KJ. Una vez alcanzada la temperatura indicada, el fisioterapeuta realizará movimientos semicirculares sobre la pared vaginal durante 2 minutos en la cara anterior vaginal y durante 4 minutos en la pared vaginal posterior. Se realizarán un total de 5 sesiones con un intervalo de 7 días entre cada una de ellas.

- 1. **Tratamiento basado en EMS**

El EMSP consistirá en un programa dirigido, con un protocolo de ejercicios de CORE y suelo pélvico establecido a partir de la valoración de la fuerza, resistencia y fatigabilidad de las pacientes, con una frecuencia de dos veces por semana dirigida por fisioterapeuta y una duración de 45 minutos cada día y un periodo de 16 semanas. Se ejecutará en grupos de 8 personas y acompañado por un fisioterapeuta experimentado en ejercicio terapéutico

Cada sesión consistirá en la realización de ejercicios para activar los músculos del suelo pélvico de forma aislada y en asociación con los músculos del CORE, tanto en estático como en dinámico.

El diseño del protocolo de los ejercicios y las secuencias se basan en una adaptación del programa descrito en un estudio previo realizado en 117 mujeres climatéricas con disfunción pélvica donde se mostró mejora significativa en síntomas de IU, síntomas vaginales y en la función sexual similar a lo que se pretende analizar en este proyecto^47^, y en los estudios previos sobre ejercicio terapéutico en la prevención y tratamiento de patologías del suelo pélvico basados en el EMSP, técnica hipopresiva, trabajo del CORE y utilización de bases inestables^54-63^.

El protocolo de EMSP irá ampliando objetivos por meses y tendrá dos sesiones diferentes por semana, para fomentar la adherencia y motivación a partir de la variabilidad de los ejercicios. Se adjunta programa de ejercicios detallado como ANEXO 4.

- 1. **Determinaciones**

Las determinaciones indicadas en este apartado se realizarán antes de iniciar la intervención, 1 semana después de finalizar la intervención, a los 6 meses de finalizar la intervención y al año de finalizar la intervención.

- 1. **Valoración de la fuerza de la MSP**

Previo a la medición se solicitará a las participantes que acudan al baño para miccionar, permitiendo de este modo estandarizar, en la medida de lo posible, el volumen de la vejiga^64^. Una vez que las mujeres se encuentren en el lugar en el que van a ser medidas, se les dejará un periodo de reposo de 3 minutos en sedestación, ya que este tiempo se corresponde con el doble de tiempo de la duración de desactivación del sistema simpático^65^.

Para el registro de la fuerza, las mujeres serán colocadas en posición ginecológica o litotómica, con la región genital y las piernas sin ropa, cubiertas por una sábana. A continuación, serán instruidas a permanecer relajadas^66^.

La evaluación de la fuerza y función de la MSP parece ser mejor cuando es determinada a través de la combinación de la observación, palpación y presión intravaginal. Por ello, en este estudio se evaluará la fuerza de la MSP tanto bidigital con la escala Oxford como a través de sonda de presión.

El primer examen consistirá en una palpación bidigital para estimar la fuerza de la MSP durante la contracción máxima mediante la escala Oxford en base a los estudios previos que nos indican la influencia de la fuerza, resistencia y fatigabilidad en la competencia del suelo pélvico y su relación con la musculatura sinérgica. Se solicitará:

- FUNCIÓN DE LA MSP: el mejor de los 3 intentos graduados como 0= no hay contracción, 1= contracción parcial, 2= contracción del SP+ contracción con músculos relacionados, 3= contracción aislada del SP^67^
- FUERZA MUSCULAR: se define como la fuerza máxima que el músculo puede generar y es descrita como la fuerza máxima que puede ejercer un músculo^68^, siendo la mejor de los 3 intentos, valorados con la escala OXFORD modificada^69^. ORDEN: “contrae la musculatura tan fuerte como sea posible”
- RESISTENCIA MUSCULAR ESTÁTICA: entendida como la capacidad para mantener una contracción óptima (isométrica)60 tanto tiempo como fuera posible, midiendo los segundos hasta llegar a la fatiga^70^. ORDEN: “mantén la contracción”, siendo la media de las tres valoraciones.
- FATIGABILIDAD O RESISTENCIA DINÁMICA: máximo número de contracciones en una unidad de tiempo, tomándose como media los 10 segundos. ORDEN: “contrae tantas veces como sea posible y a la mayor velocidad”, siendo la medida la media de las tres valoraciones^70^.

La escala de Oxford permite valorar la capacidad contráctil de los músculos del suelo pélvico. Puntúa de 0 a 5, de la siguiente forma: si hay ausencia de contracción se valora en 0, si la contracción es muy débil se valora en 1, si la contracción es débil 2, si la contracción es moderada/con tensión/y mantenida es 3, si la contracción es buena y mantiene la tensión con resistencia es 4, y si la contracción es fuerte y mantiene la tensión contra una fuerza resistente es 5^71^.

Todos los datos serán registrados en una hoja registro de fuerza de la MSP (ANEXO 5)

Con el objetivo de complementar la medición táctil y mediante pelvímetro en base a revisiones previas,^72^ se utilizará la sonda hinchable vaginal conectada a un manómetro de Phenix, que conectada a una pantalla nos permitirá valorar los conceptos de fuerza y resistencia evaluadas.

Para ello se utilizará el dispositivo terapéutico de estimulación neuromuscular y manometría de la serie PHENIX (modelo: PHENIX LIBERTY; fabricante: ELECTRONIC CONCEPT LIGNON INNOVATION, Montpeilier, Francia). Para esta manometría se utilizará la sonda de aire, conectada al sistema de biorretroalimentación Phenix, cubierta por un cubresondas de látex lubricado con gel. En el procedimiento se abren los labios mayores con una mano y se rota lentamente hacia la vagina sujetando la parte posterior de la sonda manométrica con la otra mano. Se medirá el tono basal en g antes de realizar las mediciones de la fuerza de la MSP. La sonda de manometría extraerá las señales de presión del suelo pélvico midiendo tanto su tono basal como la presión máxima mantenida durante 10 segundos en tres mediciones, calculándose la media de las tres ante la orden, “contrae lo máximo que puedas el mayor tiempo posible”.

- 1. **Función pélvica y calidad de vida**

Para evaluar la función pélvica y el impacto de las disfunciones pélvicas en la calidad de vida se facilitarán los siguientes cuestionarios (ANEXO 6):

- El cuestionario PFIQ-20 (Pelvic Floor Impact Questionnaire Short Form) permite evaluar el impacto de los síntomas urinarios, síntomas colo-rectales-anales y síntomas del prolapso genital^51^. Este cuestionario se empleará además como criterio de inclusión, para homogeneizar las características de la población en términos de disfunción pélvica previo a la intervención, como se ha indicado anteriormente.

- El ICIQ-SF (International Consultation on Incontinence Questionnaire – Short Form) es un cuestionario autoadministrado de cuatro preguntas que identifica a las personas con incontinencia de orina evaluando la frecuencia, la gravedad y el impacto en la calidad de vida. Está compuesto por cinco preguntas que evalúan la frecuencia, gravedad e impacto de la IU, además de un conjunto de ocho ítems de autodiagnóstico relacionados con situaciones de IU vividas por los pacientes. La suma máxima de los valores de respuesta indica una puntuación de 21 puntos, en referencia al alto impacto de la IU en la vida de un individuo^73^.

- Test de severidad de Sandvik. Permite conocer la severidad de la IU mediante dos cuestiones. La interpretación en base a la puntuación se clasifica en: 1-2 IU leve, 3-6 IU moderada, 8-9 IU grave, 12 IU muy grave^74^.

- 1. **Síntomas vaginales**

Para evaluar los síntomas vaginales se facilitará el siguiente cuestionario (ANEXO 7):

- Mediante examen físico se evaluará el índice de salud vaginal (VHI), que consta de una escala graduada de 1 a 5 para cada ítem (elasticidad vaginal, volumen de líquido, pH, integridad del epitelio y humedad)^53^. La elasticidad vaginal varía entre 1 (sin elasticidad) y 5 (excelente elasticidad), evaluada mediante la distensión de la mucosa a la palpación y en la colocación del espéculo. El volumen de líquido, evaluado durante la inspección, varía entre 1 (sin secreción) y 5 (secreción normal) (floculento blanco). La integridad del epitelio varía entre 1 (petequias ya detectadas en la inspección) y 5 (tejido no friable y mucosa normal). La humedad varía entre 1 (no se detecta humedad en la inspección y presencia de mucosa inflamada) y hasta 5 (humedad normal). El pH se cuantificará utilizando una tira indicadora de pH entre 0 y 14 (MColorpHast™—tiras indicadoras de pH) que se colocará directamente en la pared vaginal lateral derecha durante un minuto, dando 1 punto para el pH de 6,1, 2 para pH 5,6-6,0, 3 para pH 5,1-5,5, 4 para pH 4,7-5,0 y 5 para pH ≤ 4,6. La suma de todos los ítems representa la puntuación de salud vaginal, donde 25 representa la mejor salud vaginal^76^.

- 1. **Función sexual y autoestima**

Para evaluar la función sexual y autoestima se facilitarán los siguientes cuestionarios (ANEXO 8):

- El cuestionario FSFI (Índice Función Sexual Femenina) que evalúa la función sexual de las últimas 4 semanas. Se considera disfunción sexual el punto de corte ≤ 26,5 y el aumento del puntaje se considera una mejoría^53^.

- Dispareunia evaluada mediante la Escala Visual Analógica (EVA), que permite medir la intensidad del dolor que describe el paciente con la máxima reproducibilidad entre los observadores. Consiste en una línea horizontal de 10 centímetros, en cuyos extremos se encuentran las expresiones extremas de un síntoma. En el izquierdo se ubica la ausencia o menor intensidad y en el derecho la mayor intensidad. Se pide al paciente que marque en la línea el punto que indique la intensidad del dolor durante las relaciones sexuales y se mide con una regla milimetrada. La intensidad se expresa en centímetros o milímetros. La valoración será: 1 Dolor leve si el paciente puntúa el dolor como menor de 3; 2 Dolor moderado si la valoración se sitúa entre 4 y 7; 3 Dolor severo si la valoración es igual o superior a 8.

- Escala de Imagen corporal (S-BIS). Consta de 10 ítems que evalúan varias dimensiones de imagen corporal en pacientes con cáncer, evaluando: afectividad, conducta y cognitiva. Los productos que se califican en una escala de cuatro puntos (0: nada; 1: un poco; 2: bastante; 3: mucho) la puntuación máxima posible es de 3 puntos. A mayor puntuación obtenida mayor problemática relacionada con la imagen corporal. Su brevedad facilita una evaluación rápida, tanto en el ámbito clínico y como en el de investigación. Muestra una estructura factorial estable entre las muestras, y buenas propiedades psicométricas con una alta fiabilidad (alfa de Cronbach=0,93), lo que sugiere que se trata de una medida adecuada y útil para evaluar imagen corporal en pacientes con cáncer de mama.

- Al finalizar la intervención, se administrará un cuestionario tipo escala Likert de cinco puntos para conocer la satisfacción con el tratamiento que se clasificará en: 1 (muy insatisfecho), 2 (insatisfecho), 3 (sin cambios), 4 (satisfecho), 5 (muy satisfecho). Se considerará que las mujeres que falten a alguna de las sesiones de RF y/o cuya asistencia a las sesiones de fisioterapia presencial (RF y/o EMSP) no alcance el 80% no cumplen con el protocolo del estudio y se dará por terminada su participación, aunque serán incluidas en el análisis (por intención de tratar).

- 1. **Análisis estadístico**

Se empleará la prueba de Kolmogorov-Smirnov para evaluar la normalidad de la muestra. Los análisis comparativos entre los grupos se realizarán mediante análisis de varianza (ANOVA) o prueba de Kruskal-Wallis. Las asociaciones de variables categóricas se analizarán mediante la prueba de Chi cuadrado y la prueba exacta de Fisher. La evaluación intragrupo se realizará mediante la prueba de Wilcoxon o prueba t de Student para muestras pareadas para variables continuas, y mediante la prueba de McNemar o prueba de simetría de Bowker para variables categóricas. Los datos también se evaluarán utilizando ANOVA para medidas repetidas con el objetivo de verificar simultáneamente la influencia de los tres grupos de estudio (efecto entre grupos), las dos evaluaciones (efecto dentro de los grupos) y estimar el efecto de interacción grupo × tiempo para cada una de las variables. Los resultados se analizarán por intención de tratar. El nivel de significancia será del 5%. Se empleará el programa SPSS v.29. El análisis estadístico de los datos recogidos lo realizará un investigador (Jesús Sánchez) cegado para la intervención y para la recogida de datos.

- 1. **Material empleado**

El grupo de investigación registrado (GIR) en el Universidad UCH CEU con título Fisioterapia Activa, Terapia Manual y Análisis de Imagen (FAMI) dispone del equipamiento y el personal adecuado que garantice la viabilidad del proyecto. Se emplearán las instalaciones de la asociación AMACMEC para la inclusión de las participantes, realización de la intervención y recogida de datos.

Respecto al equipamiento, se dispone de lo siguiente relacionado con el proyecto:

- Equipo de radiofrecuencia: dispositivo Capernergy® modelo C500 Urogyne

- Equipo de biorretroalimentación: PHENIX (modelo: PHENIX LIBERTY; fabricante: ELECTRONIC CONCEPT LIGNON INNOVATION, Montpeilier, Francia).

- 1. **Cronograma**

**Tabla 1.** Cronograma estimado para una duración de dos años, dependiendo del mes de inicio desde su aprobación por el Comité.


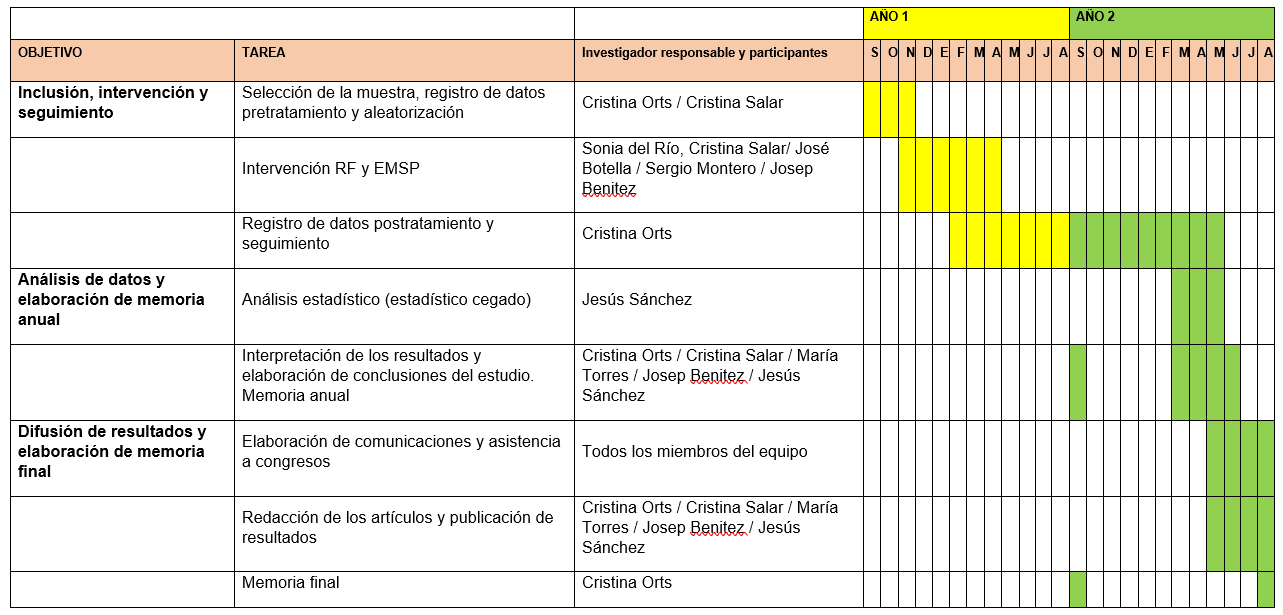


1. **Impacto esperado de los resultados**
   1. **Impacto científico-técnico**

El impacto científico-técnico de este proyecto reside en que permitirá proporcionar evidencia científica del efecto de tratamientos conservadores y no invasivos como son la radiofrecuencia y el ejercicio físico terapéutico en pacientes oncológicas, permitiendo reducir el impacto que tiene tanto el proceso oncológico como los efectos derivados de los tratamientos farmacológicos y quirúrgicos propuestos sobre la calidad de vida en esta población.

Todo ello contribuirá a que sea posible disponer de una mayor evidencia científica de la fisioterapia en el campo de la oncología, permitiendo desarrollar nuevas líneas de investigación tanto de radiofrecuencia como de ejercicio físico terapéutico en pacientes con otro tipo de procesos oncológicos, así como en pacientes con síndrome genitourinario derivado tanto de la menopausia como del uso de otros tipos de tratamientos y/o intervenciones que deriven en él.

Con las conclusiones obtenidas de este estudio se podrán desarrollar protocolos de tratamiento para la prevención y manejo de las disfunciones del suelo pélvico en pacientes oncológicos, así como poder mejorar la formación de los profesionales sanitarios en el ámbito de la fisioterapia en el tratamiento del paciente oncológico.

En el año 2020 la Europe region World Physiotherapy (ER-WCPT) señaló la importancia de la especialización en fisioterapia obedeciendo a la necesidad tanto de los propios profesionales como de la sociedad. Por este motivo, en los últimos años la ER-WCPT ha desarrollado un proceso de especialización para los fisioterapeutas y este estudio pretende contribuir a la especialización de la fisioterapia aplicando técnicas propias de esta disciplina en el campo de la oncología

- 1. **Impacto social y económico**

Este estudio permitirá desarrollar campañas de promoción de la salud, sensibilizando tanto a la sociedad como a los propios profesionales sanitarios de la necesidad de que las mujeres diagnosticadas de cáncer de mama puedan prevenir la aparición de disfunciones del suelo pélvico que se desarrollan en ellas, principalmente derivadas de los tratamientos quirúrgicos y farmacológicos a los que son sometidas.

En general, existe un desconocimiento entre los distintos profesionales sanitarios sobre la relación de las disfunciones del suelo pélvico y los tratamientos oncológicos. Esta situación lleva a una reducción de la calidad de vida de estas mujeres, por lo que los resultados de este estudio pretenden aportar evidencia científica para que el resto de profesionales sanitarios conozcan los efectos que tanto la radiofrecuencia como el ejercicio físico terapéutico producen en las mujeres con cáncer de mama, pudiendo contribuir no sólo a una mejor funcionalidad del suelo pélvico si no a una posible mejora de la autoimagen de estas pacientes, que en muchas ocasiones sufren un notable detrimento como consecuencia de los tratamientos recibidos.

Así mismo, los resultados esperados de este estudio permitirían reducir los costes de atención médica y psicológica en estas pacientes, lo que se traduciría en un ahorro significativo para el sistema sanitario, así como para la propia economía de las pacientes. Asociado a ello, se reducirían en número de bajas laborales, contribuyendo a una reducción de los costes económicos derivados de los procesos oncológicos, teniendo un impacto económico y social positivo.

- 1. **Viabilidad del proyecto y aplicabilidad**

Respecto a la viabilidad de este estudio, tanto la radiofrecuencia como el entrenamiento de la musculatura del suelo pélvico son técnicas que han mostrado evidencia científica en pacientes con síntomas genitourinarios no diagnosticados de cáncer de mama, por este motivo, se sugiere implementar estas técnicas de fisioterapia a esta población con el objetivo de conocer los efectos que producen en las mujeres diagnosticadas de cáncer de mama.

El hecho de que este estudio sea una continuación derivada de un estudio previo realizado por el grupo (IDOC22-07) en el que se caracterizó la disfunción pélvica presente en las pacientes con cáncer de mama asociadas de AMACMEC, donde además se desarrollaron talleres de información y sensibilización sobre las disfunciones de suelo pélvico, garantiza alcanzar la muestra propuesta para este estudio en los distintos grupos de intervención.

El estudio tendrá una aplicación clínica directa en el caso de que se puedan objetivar resultados positivos en las disfunciones del suelo pélvico, así como en la mejora de la calidad de vida y autopercepción de estas mujeres. La consecución del presente estudio permitirá diseñar nuevos estudios para colaborar con esta asociación, así como incorporar a otras asociaciones nacionales de pacientes con cáncer de mama y asociaciones relacionadas con otros tipos de cáncer donde la evolución de la enfermedad y los tratamientos empleados derivan en impacto similar en el suelo pélvico.

1. **ANEXOS**

**Anexo 1. Hoja de información al paciente**

**Anexo 2. Consentimiento informado**

**Anexo 3. Cuestionario**

**Anexo 4. Hoja de registro de fuerza de la musculatura del suelo pélvico**

1. **REFERENCIAS BIBLIOGRÁFICAS**

1.Cancer Today [Internet]. [citado 22 de abril de 2024]. Disponible en: <https://gco.iarc.who.int/today/>

2.Dimensiones del cáncer | AECC Observatorio [Internet]. [citado 22 de abril de 2024]. Disponible en: <https://observatorio.contraelcancer.es/explora/dimensiones-del-cancer>.

3.Cáncer de mama [Internet]. [citado 22 de abril de 2024]. Disponible en: <https://www.who.int/es/news-room/fact-sheets/detail/breast-cancer>

4.Toxicidad financiera del cáncer de mama | AECC Observatorio [Internet]. [citado 22 de abril de 2024]. Disponible en: <https://observatorio.contraelcancer.es/informes/toxicidad-financiera-del-cancer-de-mama>

5.Knaul FM, López Carrillo L, Lazcano Ponce E, Gómez Dantés H, Romieu I, Torres G. Cáncer de mama: un reto para la sociedad y los sistemas de salud. Salud Pública México. enero de 2009;51:s138-40.

6.Marzo-Castillejo M, Vela-Vallespín C, Bellas-Beceiro B, Bartolomé-Moreno C, Melús-Palazón E, Vilarrubí-Estrella M, et al. Marzo-Castillejo M, Vela-Vallespín C, Bellas-Beceiro B, Bartolomé-Moreno C, Melús-Palazón E, Vilarrubí-Estrella M, Nuin-Villanueva M. Recomendaciones de prevención del cáncer. Actualización PAPPS 2018;50 Suppl 1(Suppl 1):41-65. Spanish. doi: 10.1016/S0212-6567(18)30362-7.

7.Rui L, Guijuan Z, Fengjie B. Eugenol supresses the development of estrogen receptor-positive precan- cerous breast lesions and regulates estrogen receptor-related proteins. Acta Medica Mediterr.2018;(6):1821-7.

8.Boswell EN, Dizon DS. Breast cancer and sexual function. Transl Androl Urol. 2015;4(2):160-8.

9.WHO-MHP-HPS-EML-2021.02-eng.pdf [Internet]. [citado 23 de mayo de 2024]. Disponible en: <https://iris.who.int/bitstream/handle/10665/345533/WHO-MHP-HPS-EML-2021.02-eng.pdf>

10.Vivar CG. Impacto psicosocial del cáncer de mama en la etapa de larga supervivencia: propuesta de un plan de cuidados integral para supervivientes. Aten Primaria. 2012;44(5):288-92.

11.Jun EY, Kim S, Chang SB, Oh K, Kang HS, Kang SS. The effect of a sexual life reframing program on marital intimacy, body image, and sexual function among breast cancer survivors. Cancer Nurs. 2011;34(2):142-9.

12.Quality of Life after Breast Cancer: Survivorship and Sexuality - Dizon - 2009 - The Breast Journal - Wiley Online Library [Internet]. [citado 22 de abril de 2024]. Disponible en: <https://onlinelibrary.wiley.com/doi/10.1111/j.1524-4741.2009.00766.x>

13.Abdi F, Rahnemaei FA, Roozbeh N, Pakzad R. Impact of phytoestrogens on treatment of urogenital menopause symptoms: A systematic review of randomized clinical trials. Eur J Obstet Gynecol Reprod Biol. 2021;261:222-35.

14.Crean-Tate KK, Faubion SS, Pederson HJ, Vencill JA, Batur P. Management of genitourinary syndrome of menopause in female cancer patients: a focus on vaginal hormonal therapy. Am J Obstet Gynecol. 2020;222(2):103-13.

15.Portman DJ, Gass MLS, Vulvovaginal Atrophy Terminology Consensus Conference Panel. Genitourinary syndrome of menopause: new terminology for vulvovaginal atrophy from the International Society for the Study of Women’s Sexual Health and the North American Menopause Society. Menopause N Y N. 2014;21(10):1063-8.

16.Colombage UN, Lin KY, Soh SE, Frawley HC. Prevalence and impact of bladder and bowel disorders in women with breast cancer: A systematic review with meta-analysis. Neurourol Urodyn. 2021;40(1):15-27.

17.Sousa M, Peate M, Lewis C, Jarvis S, Willis A, Hickey M, et al. Exploring knowledge, attitudes and experience of genitourinary symptoms in women with early breast cancer on adjuvant endocrine therapy. Eur J Cancer Care (Engl). 2018;27(2):e12820.

18.Córdoba-de Juan C, Arranz-Martín B, Torres-Lacomba M. Disfunción sexual en mujeres diagnosticadas y tratadas de cáncer de mama. Estudio descriptivo longitudinal. Fisioterapia. 2019;41(2):73-82.

19.Pennycuff JF, Desale S, Wang H, Zhang G, Richter LA. Prevalence of pelvic floor disorders, associations of endocrine therapy, and surgical intervention among breast cancer survivors. Int Urogynecology J. 2022;33(9):2421-6.

20.Robinson PJ, Bell RJ, Christakis MK, Ivezic SR, Davis SR. Aromatase Inhibitors Are Associated With Low Sexual Desire Causing Distress and Fecal Incontinence in Women: An Observational Study. J Sex Med. 2017;14(12):1566-74.

21.Stahlschmidt R, Ferracini AC, Medeiros LM de, Souza CM de, Juliato CRT, Mazzola PG. Urinary Incontinence and Overactive Bladder Symptoms in Women with Breast Cancer Being Treated with Oral Hormone Therapy. Rev Bras Ginecol E Obstet Rev Fed Bras Soc Ginecol E Obstet. 2020;42(11):726-30.

22.Cárcamo M, Baquedano H, Díaz D, Díaz G P. Caracterización de incontinencia urinaria en mujeres premenopáusicas con cáncer de mama en tratamiento con tamoxifeno por cinco años en el Instituto Nacional del Cáncer. Rev Médica Clínica Las Condes. 2020;31(3):352-7.

23.Oberguggenberger A, Martini C, Huber N, Fallowfield L, Hubalek M, Daniaux M, et al. Self-reported sexual health: Breast cancer survivors compared to women from the general population - an observational study. BMC Cancer. 2017;17(1):599.

24 .Crandall CJ, Mehta JM, Manson JE. Management of Menopausal Symptoms: A Review. JAMA. 2023;329(5):405-20.

25.Riggs JA. Retropubic cystourethropexy: a review of two operative procedures with long-term follow-up. Obstet Gynecol. 1986;68(1):98-105.

26.Lapitan MCM, Cody JD, Grant A. Open retropubic colposuspension for urinary incontinence in women. Cochrane Database Syst Rev. 2009;(2):CD002912.

27.Intervenciones con cabestrillos mediouretrales para la incontinencia urinaria de esfuerzo en mujeres [Internet]. [citado 24 de abril de 2024]. Disponible en: <https://www.cochrane.org/es/CD006375/INCONT_intervenciones-con-cabestrillos-mediouretrales-para-la-incontinencia-urinaria-de-esfuerzo-en-mujeres>

28.McGuire EJ. Urethral bulking agents. Nat Clin Pract Urol. 2006;3(5):234-5.

29.Kuuva N, Nilsson CG. A nationwide analysis of complications associated with the tension-free vaginal tape (TVT) procedure. Acta Obstet Gynecol Scand. 2002;81(1):72-7.

30.Nilsson CG, Falconer C, Rezapour M. Seven-year follow-up of the tension-free vaginal tape procedure for treatment of urinary incontinence. Obstet Gynecol. 2004;104(6):1259-62.

31.Eisner H, McIntosh GV. Pubovaginal Sling. En: StatPearls [Internet]. Treasure Island (FL): StatPearls Publishing; 2024 [citado 25 de abril de 2024]. Disponible en: http://www.ncbi.nlm.nih.gov/books/NBK572150/

32.Santen RJ, Stuenkel CA, Davis SR, Pinkerton JV, Gompel A, Lumsden MA. Managing Menopausal Symptoms and Associated Clinical Issues in Breast Cancer Survivors. J Clin Endocrinol Metab. 2017;102(10):3647-61.

33.Shifren JL. Genitourinary Syndrome of Menopause. Clin Obstet Gynecol. 2018;61(3):508-16.

34.Lukacz ES, Santiago-Lastra Y, Albo ME, Brubaker L. Urinary Incontinence in Women: A Review. JAMA. 2017;318(16):1592-604.

35.The 2023 Nonhormone Therapy Position Statement of The North American Menopause Society. Advisory Panel. The 2023 nonhormone therapy position statement of The North American Menopause Society. Menopause N Y N. 2023;30(6):573-90.

36.Menown SJ, Tello JA. Neurokinin 3 Receptor Antagonists Compared With Serotonin Norepinephrine Reuptake Inhibitors for Non-Hormonal Treatment of Menopausal Hot Flushes: A Systematic Qualitative Review. Adv Ther. 2021;38(10):5025-45.

37.Wiley.com [Internet]. [citado 25 de abril de 2024]. Female Sexual Pain Disorders: Evaluation and Management, 2nd Edition | Wiley. Disponible en: <https://www.wiley.com/en-us/Female+Sexual+Pain+Disorders%3A+Evaluation+and+Management%2C+2nd+Edition-p-9781119482598>

38.Colombage UN, Soh SE, Lin KY, Kruger J, Frawley HC. The feasibility of pelvic floor training to treat urinary incontinence in women with breast cancer: a telehealth intervention trial. Breast Cancer Tokyo Jpn. 2023;30(1):121-30.

39.Seav SM, Dominick SA, Stepanyuk B, Gorman JR, Chingos DT, Ehren JL, et al. Management of sexual dysfunction in breast cancer survivors: a systematic review. Womens Midlife Health. 2015;1:9.

40.Pérez-López FR, Phillips N, Vieira-Baptista P, Cohen-Sacher B, Fialho SCAV, Stockdale CK. Management of postmenopausal vulvovaginal atrophy: recommendations of the International Society for the Study of Vulvovaginal Disease. Gynecol Endocrinol Off J Int Soc Gynecol Endocrinol. 2021;37(8):746-52.

41.Schvartzman R, Schvartzman L, Ferreira CF, Vettorazzi J, Bertotto A, Wender MCO. Physical Therapy Intervention for Women With Dyspareunia: A Randomized Clinical Trial. J Sex Marital Ther. 2019;45(5):378-94.

42.The NAMS 2020 GSM Position Statement Editorial Panel. The 2020 genitourinary syndrome of menopause position statement of The North American Menopause Society. Menopause N Y N. 2020;27(9):976-92.

43.Espitia de la Hoz FJ, Orozco Gallego H. Abordaje diagnóstico y terapéutico del síndrome genitourinario en la menopausia; actualización. Rev Médica Univ Costa Rica. 2017;11(2):67-84.

44.Mercier J, Morin M, Zaki D, Reichetzer B, Lemieux MC, Khalifé S, et al. Pelvic floor muscle training as a treatment for genitourinary syndrome of menopause: A single-arm feasibility study. Maturitas. 2019;125:57-62.

45.Kamilos MF, Borrelli CL. New therapeutic option in genitourinary syndrome of menopause: pilot study using microablative fractional radiofrequency. Einstein Sao Paulo Braz. 2017;15(4):445-51.

46.Sarmento AC, Fernandes FS, Marconi C, Giraldo PC, Eleutério-Júnior J, Crispim JC, et al. Impact of microablative fractional radiofrequency on the vaginal health, microbiota, and cellularity of postmenopausal women. Clin Sao Paulo Braz. 2020;75:e1750.

47.Slongo H, Lunardi ALB, Riccetto CLZ, Machado HC, Juliato CRT. Microablative radiofrequency versus pelvic floor muscle training for stress urinary incontinence: a randomized controlled trial. Int Urogynecology J. 2022;33(1):53-64.

48.Derbyshire M. Radiofrecuencia fraccionada microablativa como opción terapéutica para el liquen escleroso vulvar: estudio piloto [Internet]. International Menopause Society. 2022 [citado 23 de mayo de 2024]. Disponible en: <https://www.imsociety.org/2022/08/09/radiofrecuencia-fraccionada-microablativa-como-opcion-terapeutica-para-el-liquen-escleroso-vulvar-estudio-piloto/>

49.Millheiser LS, Pauls RN, Herbst SJ, Chen BH. Radiofrequency treatment of vaginal laxity after vaginal delivery: nonsurgical vaginal tightening. J Sex Med. 2010;7(9):3088-95.

50.Wańczyk-Baszak J, Woźniak S, Milejski B, Paszkowski T. Genitourinary syndrome of menopause treatment using lasers and temperature-controlled radiofrequency. Przegla̜d Menopauzalny Menopause Rev. 2018;17(4):180-4.

51.Sánchez-Sánchez B, Torres-Lacomba M, Yuste-Sánchez MJ, Navarro-Brazález B, Pacheco-da-Costa S, Gutiérrez-Ortega C, et al. Cultural adaptation and validation of the Pelvic Floor Distress Inventory short form (PFDI-20) and Pelvic Floor Impact Questionnaire short form (PFIQ-7) Spanish versions. Eur J Obstet Gynecol Reprod Biol. 2013;170(1):281-5.

52.Lalji S, Lozanova P. Evaluation of the safety and efficacy of a monopolar nonablative radiofrequency device for the improvement of vulvo‐vaginal laxity and urinary incontinence. J Cosmet Dermatol. 2017;16(2):230-4.

53.Pinheiro C, Costa T, Amorim de Jesus R, Campos R, Brim R, Teles A, et al. Intravaginal nonablative radiofrequency in the treatment of genitourinary syndrome of menopause symptoms: a single-arm pilot study. BMC Womens Health. 2021;21(1):379.

54.Zachovajeviene B, Siupsinskas L, Zachovajevas P, Venclovas Z, Milonas D. Effect of diaphragm and abdominal muscle training on pelvic floor strength and endurance: results of a prospective randomized trial. Sci Rep. 2019; 9(1):19192.

55.Molina-Torres G, Moreno-Muñoz M, Rebullido TR, Castellote-Caballero Y, Bergamin M, Gobbo S, Hita-Contreras F, Cruz-Diaz D. The effects of an 8-week hypopressive exercise training program on urinary incontinence and pelvic floor muscle activation: A randomized controlled trial. Neurourol Urodyn. 2023;42(2):500-509.

56.Skaug KL, Engh ME, Bø K. Pelvic floor muscle training in female functional fitness exercisers: an assessor-blindedrandomised controlled trial. Br J Sports Med. 2024.

57. Jorge CH, Bø K, Chiazuto Catai C, Oliveira Brito LG, Driusso P, Kolberg Tennfjord M. Pelvic floor muscle training as treatment for female sexual dysfunction: a systematic review and meta-analysis. Am J Obstet Gynecol. 2024.

58. Tibaek S, Dehlendorff C. Pelvic floor muscle function in women with pelvic floor dysfunction: a retrospective chart review, 1992-2008. Int Urogynecol J. 2014;25(5):663-9.

59. Gimenez MM, Fitz FF, de Azevedo Ferreira L, Bortolini MAT, Lordêlo PVS, Castro RA. Pelvic floor muscle functiondiffers between supine and standing positions in women with stress urinary incontinence: an experimental crossover study. J Physiother. 2022;68(1):51-60.

60. McClurg D, Frawley H, Hay-Smith J, Dean S, Chen SY, Chiarelli P, Mair F, Dumoulin C. Scoping review of adherencepromotion theories in pelvic floor muscle training - 2011 ICS state-of-the-science seminar research paper i of iv. Neurourol Urodyn. 2015;34(7):606-14.

61. Dakic JG, Cook J, Hay-Smith J, Lin KY, Frawley H. Pelvic floor disorders stop women exercising: A survey of 4556 symptomatic women. J Sci Med Sport. 2021;24(12):1211-1217.

62. Sapsford RR, Hodges PW, Richardson CA, Cooper DH, Markwell SJ, Jull GA. Co-activation of the abdominal and pelvic floor muscles during voluntary exercises. Neurourol Urodyn. 2001; 20(1):31-42.

63. Radzimińska A, Strączyńska A, Weber-Rajek M, Styczyńska H, Strojek K, Piekorz Z. The impact of pelvic floor muscle training on the quality of life of women with urinary incontinence: a systematic literature review. Clin Interv Aging. 2018; 13:957-965.

64.Sapsford RR, Richardson CA, Stanton WR. Sitting posture affects pelvic floor muscle activity in parous women: an observational study. Aust J Physiother. 2006;52(3):219-22.

65.Fisiología Clínica del Ejercicio de José López Chicharro | España | Editorial Médica Panamericana [Internet]. [citado 23 de mayo de 2024]. Disponible en: https://www.medicapanamericana.com/es/libro/fisiologia-clinica-del-ejercicio

66.Romero-Cullerés G, Peña-Pitarch E, Jané-Feixas C, Arnau A, Montesinos J, Abenoza-Guardiola M. Intra-rater reliability and diagnostic accuracy of a new vaginal dynamometer to measure pelvic floor muscle strength in women with urinary incontinence. Neurourol Urodyn. 2017;36(2):333-7.

67. Tibaek, S., Gard, G., Dehlendorff, C., Iversen, H. K., Erdal, J., Biering-Sorensen, F. Jensen, R. 2015. The effect of pelvic floor muscle training on sexual function in men

with lower urinary tract symptoms after stroke. Topics in Stroke Rehabilitation, 22, 185-193.

68.Kenney WL, Wilmore JH, Costill DL, Wilmore JH. Physiology of sport and exercise. 5th ed. Champaign, IL: Human Kinetics; 2012. 621.

69. Laycock, J. Clinical evaluation os the pelvic floor. In B. Schüssler, J. Laycock, P. Norton, & S. L. Stanton (Eds.), Pelvic floor re-education. London, England: Springer-Verlag.1994: 42-28.

70.Tibaek S, Gard G, Dehlendorff C, Iversen HK, Biering-Soerensen F, Jensen R. Is Pelvic Floor Muscle Training Effective for Men With Poststroke Lower Urinary Tract Symptoms? A Single-Blinded Randomized, Controlled Trial. Am J Mens Health. septiembre de 2017;11(5):1460-71.

71. Laycok, J. Haslam, J. Patient assesment in therapeutic management of incontinence and pelvic pain. Springer.2002.

72. Worman R, Stafford R.e., Cowley D, Hodges P.W Methods used to investigate tone of pelvic floor muscles in pelvic health conditions: A systematic review. Elsevier. 2023.

73.Espuña Pons M, Rebollo Alvarez P, Puig Clota M. [Validation of the Spanish version of the International Consultation on Incontinence Questionnaire-Short Form. A questionnaire for assessing the urinary incontinence]. Med Clin (Barc). 2004;122(8):288-92.

74.Sandvik H, Seim A, Vanvik A, Hunskaar S. A severity index for epidemiological surveys of female urinary incontinence: comparison with 48-hour pad-weighing tests. Neurourol Urodyn. 2000;19(2):137-45.

75.Tamanini JTN, Almeida FG, Girotti ME, Riccetto CLZ, Palma PCR, Rios LAS. The Portuguese validation of the International Consultation on Incontinence Questionnaire-Vaginal Symptoms (ICIQ-VS) for Brazilian women with pelvic organ prolapse. Int Urogynecol J Pelvic Floor Dysfunct. 2008;19(10):1385-91.

76. Bachmann G. Urogenital ageing: an old problem newly recognized. Maturitas. diciembre de 1995;22 Suppl:S1-5.

**ANEXOS**

**ANEXO 1**

**HOJA DE INFORMACIÓN A LOS PARTICIPANTES EN EL**

**PROYECTO DE INVESTIGACIÓN**

**TÍTULO DEL ESTUDIO:** Efectividad de la radiofrecuencia y la rehabilitación basada en el ejercicio físico sobre la sintomatología asociada a las disfunciones del suelo pélvico en pacientes con cáncer de mama. Ensayo clínico aleatorizado.

**INVESTIGADOR PRINICPAL:** *Da. Beatriz Pina Bernabeu*

*Universidad CEU Cardenal Herrera*

**INTRODUCCIÓN**

Nos dirigimos a usted para informarle sobre un estudio de investigación en el que se le invita a participar.

Nuestra intención es que usted reciba la información correcta y suficiente para que pueda decidir si acepta o no participar en este estudio. Para ello, tómese el tiempo necesario para leer detenidamente y con atención esta hoja informativa y coméntela con quien considere oportuno. Pida al personal del estudio que le explique cualquier palabra o información que no comprenda con claridad, así como cualquier duda que le surja.

Si decide que desea participar, le solicitaremos que firme el documento adjunto de consentimiento informado. Le proporcionaremos una copia original de este documento firmado y fechado para que la conserve y el documento original quedará archivado con el resto de la documentación del estudio.

El estudio ha sido aprobado por el Comité de Ética de la Investigación de la Universidad CEU Cardenal Herrera de Valencia.

Asimismo, ha sido diseñado y se realizará de acuerdo con las recomendaciones establecidas en la Declaración de Helsinki y en las Normas de Buenas Prácticas Clínicas.

Debe saber que su participación en este estudio es voluntaria y que puede decidir NO participar. Si decide participar, puede cambiar su decisión y retirar el consentimiento en cualquier momento, sin que por ello se altere la relación con su fisioterapeuta.

También debe saber que puede ser retirado del estudio si el promotor o los investigadores lo consideran oportuno, ya sea por motivos de seguridad o por otros motivos. En cualquiera de los casos, usted recibirá una explicación adecuada del motivo que ha ocasionado su retirada del estudio.

**¿POR QUÉ SE REALIZA EL ESTUDIO?**

Es sabido que la función pélvica, la salud vaginal y la función sexual se encuentran alteradas en pacientes con cáncer de mama debido al desarrollo de la enfermedad y a los tratamientos recibidos. El objetivo del estudio es conocer la efectividad del tratamiento fisioterápico basado en activación de la musculatura del suelo pélvico mediante calor inducido por radiofrecuencia y mediante el ejercicio físico para la mejora de esa sintomatología asociada al cáncer. Se trata de tratamientos no invasivos e indoloros que han sido muy eficientes en el tratamiento de estos síntomas en otros grupos de mujeres pero que aún no se ha probado en pacientes con cáncer de mama.

**¿CÓMO SE VA A REALIZAR EL ESTUDIO?**

Se realizará un tratamiento personalizado dirigido por fisioterapeutas con gran experiencia en la clínica de suelo pélvico. Las participantes serán asignadas de forma aleatoria a uno de los tres grupos de tratamiento de los que consta el estudio: estimulación mediante calor (radiofrecuencia) (grupo 1), ejercicio físico (grupo 2) o radiofrecuencia + ejercicio físico (grupo 3).

La intervención se realizará en las instalaciones de la Asociación de Mujeres Afectadas por el Cáncer de Mama de Elche y Comarca (AMACMEC), con domicilio social en Calle Olegario Domarco Seller, 93. Entresuelo de Elche (Alicante), y provista del CIF G53270146.

El estudio comenzará en septiembre de 2024. La radiofrecuencia es una terapia basada en la electroterapia que consiste en, mediante la aplicación de un electrodo porcelánico, diseñado de manera específica para el tratamiento de disfunciones del área uroginecológica, cubierto por un cubresondas de látex y que se introducirá en la vagina, generar un aumento de temperatura en los tejidos, provocando en la paciente una sensación de calor localizada en la zona de aplicación, manteniendo siempre una percepción de calor intenso no quemante. El tratamiento con radiofrecuencia consistirá en 5 sesiones de 30 minutos con un intervalo de 7 días entre cada una de ellas. El ejercicio físico consistirá en realizar un programa de ejercicios que incluyan la activación del suelo pélvico dirigido por un fisioterapeuta con una frecuencia de dos veces por semana, una duración de 45 minutos cada día y un periodo de 16 semanas. En este caso se le recomendará que continue con los ejercicios en casa y se hará un seguimiento sobre si cumple ese programa en casa o no, no siendo requisito este cumplimiento, mediante la cumplimentación de un diario que se le facilitará en formato de papel para que lo cumplimente en casa.

**¿QUÉ CRITERIOS SE DEBEN CUMPLIR PARA PODER PARTICIPAR?**

Los criterios para poder participar en el estudio serán: mujeres mayores de edad con historial clínico de cáncer de mama y que presente alguna disfunción del suelo pélvico tales como incontinencia urinaria, incontinencia fecal, prolapso o disfunción sexual. Mujeres que acepten participar en el estudio.

Los criterios por los que no podrá participar en el estudio son: haber realizado entrenamiento de la musculatura del suelo pélvico o haber recibido sesiones de radiofrecuencia en los últimos 12 meses, uso de estrógenos vaginales en los últimos 6 meses, terapia hormonal sistémica en los últimos 6 meses, terapia con láser vaginal en los últimos 6 meses, ausencia de contracción del suelo pélvico que se le evaluará en el momento en que autorice su participación en este estudio, uso de marcapasos, corazón descompensado o enfermedades metabólicas, déficit cognitivo, trastornos neurológicos periféricos o centrales, cirugías previas en la región pélvica, patologías cutáneas o heridas en la zona de tratamiento o presencia de una infección activa del tracto urinario y/o vagina.

**¿EN QUÉ CONSISTE MI PARTICIPACIÓN?**

Si accede a participar en el estudio, le pediremos:

- Firme el consentimiento informado. Toda la información recopilada será tratada de forma confidencial y su uso será meramente científico, salvaguardado en todo momento su identidad. Para ello será necesario que firme su expreso consentimiento mediante la autorización por escrito.

- Una vez aceptada la participación se le realizará una primera revisión clínica para evaluar la fuerza de la musculatura del suelo pélvico, así como adquirir datos sociodemográficos (edad, peso, trabajo, estudios), datos relacionados con el diagnóstico y tratamiento del cáncer de mama, sobre función pélvica y su impacto en su calidad de vida, estado vaginal, función sexual y autoestima mediante cuestionarios habituales y validados para este tipo de estudios.

- Se le asignará de forma aleatoria a uno de los tres grupos de intervención.

- Se le volverá a hacer una revisión clínica igual que la descrita anteriormente 1 semana después de acabar el tratamiento, a los 6 meses y al año tras acabar el tratamiento.

**¿QUÉ RIESGOS O MOLESTIAS PUEDO SUFRIR POR PARTICIPAR EN EL ESTUDIO?**

Se trata de tratamientos conservadores no invasivos e indoloros.

Respecto a la radiofrecuencia no ablativa su forma de actuar es aumentando de forma selectiva y controlada la temperatura en los tejidos. En este estudio no se sobrepasarán los 41ºC por lo que se reducirá casi por completo sus posibles efectos adversos que podrían ser pequeños eritemas o edemas propios del calentamiento de la piel. Aunque no se relaciona estos efectos adversos con esta baja temperatura, de producirse desaparecerían en 1 o 2 días.

Respecto al ejercicio físico, éste será dirigido por un profesional, garantizando la correcta realización de cada ejercicio reduciendo así la aparición de potenciales lesiones o dolores debidos a la práctica incorrecta del ejercicio físico. Al contrario, está establecido que la práctica de ejercicio físico es una recomendación para la salud física como mental.

**¿QUÉ BENEFICIOS SE OBTENDRÁN DEL ESTUDIO?**

La participante recibirá un tratamiento completo no invasivo e indoloro que, según estudios previos en otros colectivos con sintomatología similar, repercutirá positivamente en su función pélvica, en su estado vaginal, y por lo tanto tendrá impacto positivo en su calidad de vida, función sexual y en su percepción y autoestima.

Los resultados permitirán comparar la eficacia de tratamientos seguros, no invasivos y de bajo coste, obteniendo datos de gran valor clínico al permitir el desarrollo de programas de terapia conservadora para el tratamiento de sintomatología característica de pacientes que han sufrido las consecuencias del cáncer de mama y de su tratamiento.

**¿QUÉ OPCIONES DE TRATAMIENTO TENGO SI NO PARTICIPO EN EL ESTUDIO?**

No procede este apartado, ya que las pacientes de cáncer de mama ya recibieron su tratamiento por parte del equipo médico que las trató.

**¿CÓMO SE PROTEGEN MIS DERECHOS?**

1. El estudio se realizará cumpliendo todas las normas éticas y legales vigentes.

**Confidencialidad**

1. Los investigadores se comprometen a que sus datos personales serán tratados de manera confidencial y se procesarán conforme a la normativa vigente sobre protección de datos personales ([Ley Orgánica 3/2018, de Protección de Datos Personales y Garantía de los Derechos Digitales](https://correu.cs.san.gva.es/owa/redir.aspx?C=DCodJxWDnXJx2RnE3_evq_PdFrZ2DdWoVRXxIWvXdlHsXzCeKn3WCA..&URL=https%3a%2f%2fwww.boe.es%2fboe%2fdias%2f2018%2f12%2f06%2fpdfs%2fBOE-A-2018-16673.pdf), y Reglamento [UE] 2016/679 del Parlamento Europeo y del Consejo, relativo a la protección de las personas físicas en lo que respecta al tratamiento de datos personales y a la libre circulación de estos datos). Para cumplir con esta normativa los datos recogidos para el estudio estarán identificados mediante un código, de manera que no incluya información que pueda identificarle, y sólo los investigadores podrá relacionar dichos datos con usted y con su historia clínica. Por lo tanto, su identidad no será revelada a persona alguna salvo excepciones en caso de urgencia médica o requerimiento legal. El tratamiento, la comunicación y la cesión de los datos de carácter personal de todos los participantes se ajustarán a lo dispuesto en la ley.
2. El acceso a su información personal identificada quedará restringido a los investigadores, autoridades sanitarias, al Comité de Ética de la Investigación y personal autorizado por el promotor (monitores del estudio, auditores), cuando lo precisen para comprobar los datos y procedimientos del estudio, pero siempre manteniendo la confidencialidad de los mismos de acuerdo a la legislación vigente.
3. Los cuestionarios en los que aparecen datos personales serán custodiados bajo llave en todo momento y solo tendrán acceso los investigadores.
4. Los datos se recogerán de manera anónima en un fichero de investigación responsabilidad de los investigadores, se tratarán en ordenadores de la red a los que solo se puede acceder con una clave persona.
5. De acuerdo a lo que establece la legislación de protección de datos, usted puede ejercer los derechos de acceso, modificación, oposición y cancelación de datos, para lo cual deberá dirigirse a su médico del estudio.
6. Si usted decide retirar el consentimiento para participar en este estudio, ningún dato nuevo será añadido a la base de datos, pero sí se utilizarán los que ya se hayan recogido.
7. Los datos codificados pueden ser transmitidos a terceros, pero en ningún caso contendrán información que le pueda identificar directamente, como nombre y apellidos, iniciales, número de historia clínica, etc. En el caso de que se produzca esta cesión, será para los mismos fines del estudio descrito o para su uso en publicaciones científicas, pero siempre manteniendo la confidencialidad de los mismos de acuerdo a la legislación vigente.
8. Los datos recogidos nunca serán utilizados para otra finalidad, en consecuencia, todos los datos serán destruidos y/o eliminados una vez se hayan publicado los resultados de la investigación en revistas científicas.

**¿CON QUIÉN PUEDO CONTACTAR EN CASO DE DUDA?**

Si tiene alguna duda consulte con Cristina Orts Ruiz (Universidad CEU Cardenal Herrera), en el número de teléfono 965 42 64 86 Extensión: 403, que es la responsable de esta investigación y que contestará cualquier pregunta que tenga relacionada con este estudio.

1. Sea cual sea su decisión, el equipo de investigación quieren agradecer su tiempo y atención.

**ANEXO 2**

**DOCUMENTO DE CONSENTIMIENTO PARA LA PARTICIPACIÓN EN UN PROYECTO DE INVESTIGACIÓN**

***TITULO DEL PROYECTO: Efectividad de la radiofrecuencia y la rehabilitación basada en el ejercicio físico sobre la sintomatología asociada a las disfunciones del suelo pélvico en pacientes con cáncer de mama. Ensayo clínico aleatorizado.***

***INVESTIGADORA PRINCIPAL. Dra. Cristina Orts Ruiz***

**D./ Dña._______________________________________________________**

**Con DNI Nº____________________**

**Libre y voluntariamente**

**MANIFIESTO:**

1. He leído y comprendido la hoja informativa objeto del estudio.
2. He tenido la oportunidad de hacer preguntas.
3. Mis preguntas han sido respondidas de forma satisfactoria.
4. He recibido información suficiente del estudio y de las pruebas a realizar.
5. Entiendo que la participación es voluntaria y puedo abandonar el estudio cuando lo desee sin que tenga que dar explicaciones y sin que ello afecte a mis cuidados médicos.
6. De acuerdo con lo establecido por el Reglamento (UE) 2016/679 del Parlamento Europeo y del Consejo, de 27 de abril de 2016, relativo a la protección de las personas físicas en lo que respecta al tratamiento de datos personales y a la libre circulación de estos datos y por el que se deroga la Directiva 95/46/CE, he sido informado de que mis datos personales, obtenidos mediante la cumplimentación de este formulario así como los resultantes de mi participación en el proyecto van a ser tratados bajo la responsabilidad de la FUNDACIÓN UNIVERSITARIA SAN PABLO CEU (en adelante, FUSP-CEU), con la finalidad de gestionar mi participación en el presente proyecto de investigación. Además, he sido informado de los siguientes aspectos:
   1. Que está prevista la elaboración de perfiles al objeto de analizar o predecir aspectos relativos a mi salud.
   2. Que los tratamientos indicados se encuentran legitimados en el consentimiento otorgado por mi parte.
   3. Que mis datos personales, obtenidos mediante la cumplimentación de este formulario, así como los resultantes de mi participación en el proyecto serán conservados durante el tiempo necesario para el desarrollo de esta investigación, que se estima de 10 meses, siendo posteriormente destruidos, sin que puedan ser conservados sin haber sido previamente anonimizados. En cualquier caso, no podrán ser cedidos sin mi consentimiento expreso y no lo otorgo en este acto.
   4. Que puedo contactar con el Delegado de Protección de Datos de FUSP-CEU, dirigiendo mi petición por escrito a la dirección postal C/ Tutor nº 35 - 28008 Madrid o a la dirección de correo electrónico [dpd@ceu.es](mailto:dpd@ceu.es).
   5. Que de acuerdo con los derechos que me confiere la normativa vigente en protección de datos podré dirigirme a la Autoridad de Control competente para presentar la reclamación que considere oportuna, así como también podré ejercer los derechos de acceso, rectificación, limitación de tratamiento, supresión, portabilidad y oposición al tratamiento de mis datos de carácter personal y retirar el consentimiento prestado para el tratamiento de los mismos, dirigiendo mi petición al investigador responsable en la dirección de contacto que figura en este documento.
7. Estoy de acuerdo en que mi consentimiento por escrito y otros datos estén a disposición del proyecto de investigación clínico en el que estoy participando, y del investigador responsable del mismo, Dra. Cristina Orts Ruiz, pero siempre respetando la confidencialidad y la garantía de que mis datos no estarán disponibles públicamente de forma que pueda ser identificado.
8. Los datos recogidos para este estudio serán incluidos, con los de otras personas que participen en este estudio, en una base de datos de carácter personal de la Universidad CEU Cardenal Herrera, a la que sólo los investigadores aprobados para este proyecto tendrán acceso, estando todos ellos sometidos al secreto inherente a su profesión o derivado de un acuerdo de confidencialidad.
9. Firmo este documento de información y consentimiento de forma voluntaria para manifestar mi deseo de participar en este estudio de investigación sobre RADIOFRECUENCIA Y EJERCICIO FÍSICO TERAÉUTICO PARA SINDROME GENITOURINARIO O DISFUNCIONES DEL SUELO PÉLVICO TRAS CÁNCER DE MAMA, hasta que decida lo contrario. Al firmar este consentimiento no renuncio a ninguno de mis derechos. Recibiré una copia de este documento para guardarlo y poder consultarlo en el futuro.

Por tanto, doy mi conformidad y consentimiento para a que realice el estudio detallado con la ayuda del personal que sea necesario con la debida cualificación y especialización.

El participante

(Firma) Nombre, Apellidos

Valencia, a ……… de ………………… de 2024

**AUTORIZACIÓN DEL FAMILIAR O TUTOR**

Ante la imposibilidad de D./Dña.

con DNI de prestar autorización para los tratamientos explicitados en el presente documento de forma libre, voluntaria, y consciente.

D./Dña.

con DNI

En calidad de (marido, esposa, hijo, hermano, tutor legal, familiar, allegado, cuidador), decido, dentro de las opciones clínicas disponibles, dar mi conformidad libre, voluntaria y consciente a la técnica descrita para los tratamientos explicitados en el presente documento.

_________, _____de _______________________de____________

**INVESTIGADORA**

D./Dña. Dra. Cristina Orts Ruiz

con DNI: 74241971H

Correo electrónico: cristina.orts@uchceu.es

Teléfono:  [96 542 64 86](tel://96%20542%2064%2086) | Ext. 67403

Investigador de la Universidad CEU-Cardenal Herrera de Valencia, declaro haber facilitado al participante del estudio y/o persona autorizada, toda la información necesaria para la realización de la intervención explicitada en el presente documento y declaro haber confirmado, inmediatamente antes de la aplicación de la técnica, que el participante no incurre en ninguno de los casos de contraindicación relacionados anteriormente, así como haber tomado todas las precauciones necesarias para que la intervención correcta.

___________, _____de _______________________de____________

**REVOCACIÓN DEL CONSENTIMIENTO INFORMADO**

D/Dña.

con DNI

Revoco el consentimiento prestado en fecha de de

Y no deseo proseguir el tratamiento que doy en esta fecha por finalizado.

______________, _____de _______________________de____________

**ANEXO 5 CUESTIONARIO *AD HOC***

**CUESTIONARIO PARA ESTUDIO DE LA EFECTIVIDAD DE LA RADIOFRECUENCIA Y LA REHABILITACIÓN BASADA EN EL EJERCICIO FÍSICO SOBRE LA SINTOMATOLOGÍA ASOCIADA A LAS DISFUNCIONES DEL SUELO PÉLVICO EN PACIENTES CON CÁNCER DE MAMA. ENSAYO CLÍNICO ALEATORIZADO**

Código de identificación del paciente:

1. Edad (años):
2. Peso (Kg):
3. Estatura (metros):
4. Nacionalidad:
5. País en el que reside:
6. Nivel superior de estudios alcanzado:
7. *Educación secundaria obligatoria (ESO)*
8. *Formación profesional básica (FPB)*
9. *Bachillerato*
10. *Ciclos formativos de grado medio*
11. *Ciclos formativos de grado superior*
12. *Estudios universitarios*
13. *Sin estudios*
14. ¿Trabaja actualmente?
15. *Sí*
16. *Jubilada*
17. *Desempleada*
18. *Estudiante*
19. Tipo de trabajo:
20. *No trabajo*
21. *Sanitario*
22. *Humanidades sociales*
23. *Servicios*
24. Número de embarazos llevados a término
25. Número de embarazos no llevados a término
26. En caso de haber estado embarazada, indique la edad a la que tuvo su primer embarazo
27. Indique el número de partos vaginales
28. Indique el número de cesáreas
29. ¿Ha dado lactancia materna?
30. *Sí*
31. *No*
32. *Sólo a algunos de mis hijos*
33. ¿Tiene usted la menopausia?
34. *Sí, desde antes del tratamiento oncológico para el cáncer de mama*
35. *Sí, desde después del tratamiento oncológico para el cáncer de mama*
36. *No*
37. En caso de haber contestado Sí, indique los años que hace que tiene la menopausia
38. ¿Fuma?
39. *Si*
40. *No*
41. *Ocasionalmente*
42. ¿Bebe alcohol?
43. *Sí*
44. *No*
45. *Ocasionalmente*
46. Indique la frecuencia con la que ha practicado deporte de intensidad moderada-alta durante los últimos 3 meses (equivalente o superior a una caminata enérgica de al menos 30min)
47. *Todos los días*
48. *2-4 veces por semana*
49. *Menos de 2 veces por semana*
50. *No practico deporte*
51. ¿Cuántos años hace que fue diagnosticada por primera vez de cáncer de mama?
52. *¿Qué tipo de tratamiento ha recibido para el cáncer de mama?*

*a. Quimioterapia*

*b. Radioterapia*

*c. Terapia Hormonal*

*d. Cirugía mamaría, sin reconstrucción*

*e. Cirugía mamaría, con reconstrucción*

*f. Cirugía axilar*

*g. Inmunoterapia*

*h. Ninguno*

1. ¿Actualmente está en tratamiento para el cáncer de mama?
2. *Sí*
3. *No*
4. En caso de estar actualmente en tratamiento, indique cuál está recibiendo
5. *Quimioterapia*
6. *Radioterapia*
7. *Terapia hormonal*
8. *Inmunoterapia*
9. Señale cuál de los siguientes síntomas ha experimentado usted tras el tratamiento recibido (marque todas las opciones que considere)
10. *Dolor en el miembro superior*
11. *Linfedema*
12. *Trombosis linfáticas superficiales*
13. *Fatiga*
14. *Incontinencia urinaria*
15. *Incontinencia fecal*
16. *Dolor pélvico*
17. *Disfunción sexual*
18. *Ninguno*
19. Señale si tiene aluno de estos síntomas (marque tantos comoconsidere)
20. *Sequedad vaginal*
21. *Dolor durante las relaciones sexuales*
22. *Reducción de la laxitud (elasticidad) vaginal*
23. *Picor vaginal*
24. *Sensación de ardor*
25. *Dolor en la entrada vaginal*
26. ¿Ha sufrido alguna recidiva del cáncer de mama?
27. *Si*
28. *No*
29. ¿Ha padecido algún otro tipo de cáncer?
30. *Si*
31. *No*
32. En caso de haber padecido otro tipo de cáncer, indique cuál o cuáles
33. ¿Cuántos años hace que fue diagnosticada de dicho cáncer?
34. ¿qué tipo de tratamiento ha recibido para este otro tipo de cáncer:
35. *Quimioterapia*
36. *Radioterapia*
37. *Terapia hormonal*
38. *Cirugía*
39. *Inmunoterapia*
40. *Ninguno*
41. ¿Está recibiendo tratamiento psicológico?
42. *Sí, a consecuencia del diagnóstico de cáncer de mama*
43. *Sí, por causas distintas al cáncer de mama*
44. *No*
45. ¿Está recibiendo o ha recibido tratamiento de fisioterapia como consecuencia del cáncer de mama?
46. *Sí, actualmente*
47. *Sí, pero actualmente no*
48. *No*
49. ¿Ha realizado rehabilitación del Suelo Pélvico tras el cáncer?
50. *Sí, actualmente*
51. *Sí, pero actualmente no*
52. *No*
53. ¿Trabaja su suelo pélvico mediante ejercicios?
54. *Sí lo trabajo actualmente*
55. *No, pero lo he trabajado*
56. *No*
57. ¿En alguna ocasión ha recibido tratamiento con estrógenos?
    1. *Sí, actualmente*
    2. *Sí, pero actualmente no*
    3. *No*

**ANEXO 4. PROGRAMA DE EJERCICIO FÍSICO**

**Programa de Ejercicio Físico enfocado al suelo pélvico.**

Las primeras 4 semanas incluiráń un día de entrenamiento del CORE y suelo pélvico en clínica y sesión individual para asegurarnos de la comprensión de conceptos y realización correcta de la técnica. Tras el mismo el protocolo de ejercicio terapéutico se basará en la propiocepción, movilización, y activación de estructuras responsables del CORE, trabajo isométrico, activación voluntaria de la musculatura del CORE, suelo pélvico y musculatura sinérgica como los glúteos. El trabajo será tanto estático como dinámico. Los ejercicios se realizarán en espiración resistida y apnea para facilitar la activación del complejo lumbo pélvico, como ya han demostrado su eficacia programas previos destinados a pacientes con disfunciones abdomino-pélvicas.

La persona que aparece en la descripción del programa de ejercicios físicos ha proporcionado su consentimiento informado por escrito (como se describe en el formulario de consentimiento de PLOS) para publicar su imagen junto con el manuscrito.

| ***Ejercicios*** | ***Descripción*** | ***Intensidad y frecuencia*** | ***Ilustraciones*** |
| --- | --- | --- | --- |
| **MES 1, Día 1** | | | |
| Sesión individual en cabina, donde se explicará conceptos anatómicos, de respiración y se explicará la realización de los ejercicios, comprobando mediante test digital la correcta realización de los mismos. Se pautarán número de contracciónes mantenidas, rápidas y tiempo de apnea según valoración inicial. | | | |
| **MES 1, Días 2, 4, 6, 8** | | | |
| **Activación de suelo pélvico y del transverso.** | Boca arriba, piernas flexionadas con apoyo de talones y flexión dorsal de tobillo. En espiración resistida autoelongación y activación del transverso y suelo pélvico. | 10 respiraciones. | 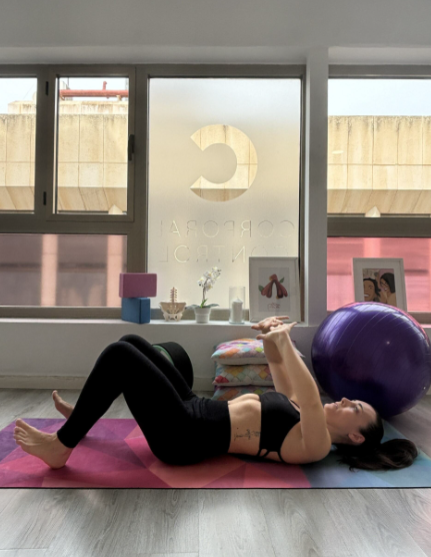 |
| **Puente glúteo.** | Boca arriba, piernas flexionadas con apoyo de talones y flexión dorsal de tobillo. En espiración resistida autoelongación y activación del transverso y suelo pélvico junto con la elevación de glúteos (puente glúteo). | 10 repeticiones. | 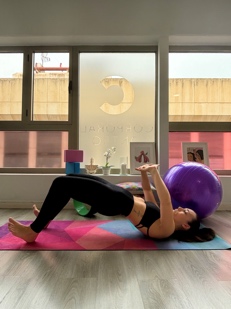 |
| **Puente glúteo con elongación pierna alternada.** | Boca arriba, piernas flexionadas con apoyo de talones. En espiración resistida autoelongación y activación del transverso y suelo pélvico y elevación de glúteos (puente glúteo) con estiramiento de pierna unilateral. Mismo ejercicio con la otra pierna. | 10 repeticiones. | 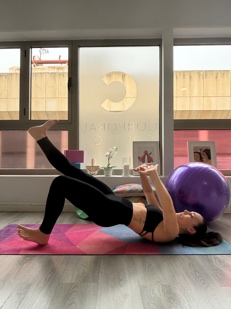 |
| **Abdominales en V piernas flexionadas con pelota.** | Boca arriba, piernas a 90 grados con pelota entre las mismas, se realiza movimiento de flexo-extensión de cadera y rodilla con activación de adductores. Se realiza con espiración en el descenso y estiramiento de las piernas. | 10 repeticiones. | 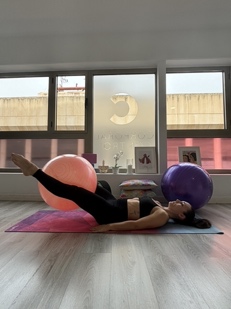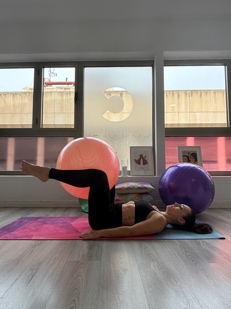 |
| **Elevación de pierna tumbada de lado.** | De lado con piernas estiradas, activación del transverso y del suelo pélvico, realizar movimiento de elevación y descenso de la pierna de arriba con amplitud máxima y velocidad lenta y luego con mínima amplitud del movimiento y máxima velocidad. | 10 repeticiones. | 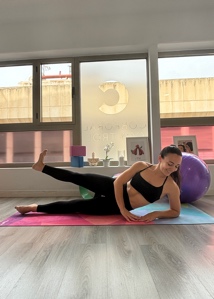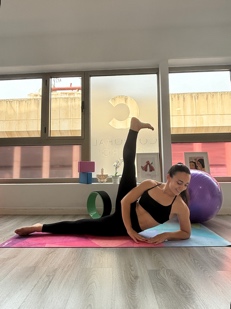 |
| **Circunducción de pierna de lado.** | De lado con piernas estiradas, activación del transverso y conciencia del suelo pélvico, realizar movimiento de círculos con la pierna de arriba en los dos sentidos. | 10 repeticiones. | 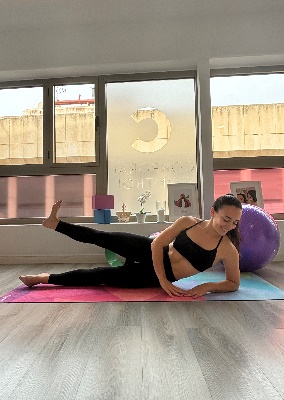 |
| **Flexión y extensión de pierna de lado.** | De lado, piernas alineadas con el tronco, activar el transverso y suelo pélvico, realizar movimiento de flexo extensión de cadera y rodilla. | 10 repeticiones. | 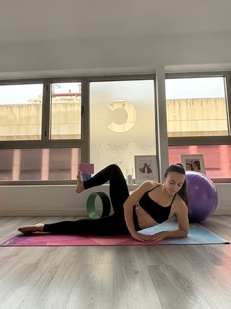 |
| **Contracciones sentada sobre Fitball.** | Sentada sobre el Fitball realizar contracciones del suelo pélvico con activación del transverso, rápidas y mantenidas en tiempo espiratorio. No quedarse en apnea. | Según parámetros de valoración que hicimos en consulta. | 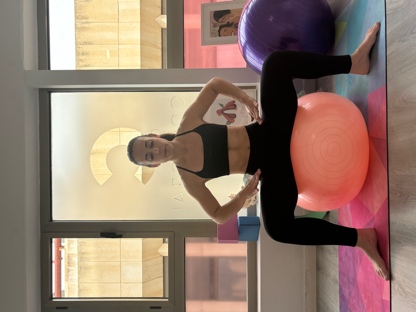 |
| **Movilidad pélvica sobre Fitball.** | Sobre Fitball movimientos de conciencia y propiocepción pélvica (realizar círculos en ambos sentidos e infinitos) para dar movilidad a la pelvis y relajar el CORE y suelo pélvico. | 60 segundos. | 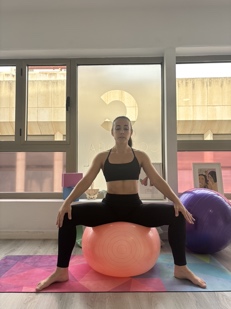 |
| **MES 1, Días 3, 5, 7** | | | |
| **Movilidad pélvica sobre Fitball.** | Sobre Fitball movimientos de conciencia y propiocepción pélvica (realizar círculos en ambos sentidos e infinitos) para dar movilidad a la pelvis y relajar el CORE y suelo pélvico. | 60 segundos. | 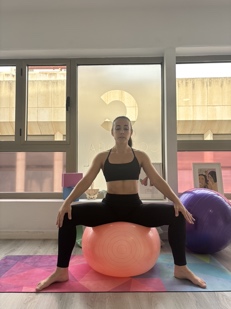 |
| **Contracciones pélvicas sobre Fitball.** | Sentada sobre el Fitball realizar contracciones del suelo pélvico con activación del transverso, rápidas y mantenidas en tiempo espiratorio. No quedarse en apnea. | Según parámetros de valoración que hicimos en consulta. | 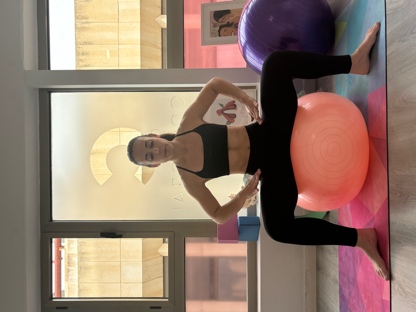 |
| **Auto elongación sobre Fitball.** | Sentado sobre el Fitball con talones apoyados y flexión dorsal de tobillo. Tras 2 respiraciones completas, quedarse en apnea y auto elongación, creciendo con activación del transverso y suelo pélvico. | Mantenimiento de 10seg. | 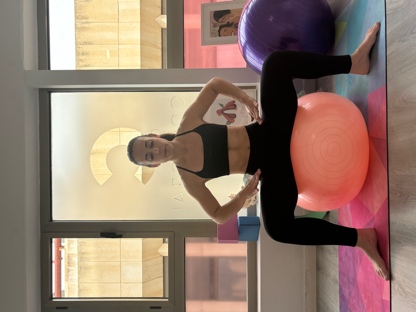 |
| **Auto elongación sobre Fitball con elevación de pierna alternada.** | Sentada sobre el Fitball tras 2 respiraciones completas, quedarse en apnea y auto elongación, creciendo, con activación del transverso y suelo pélvico con elevación de una pierna alternada. | Mantenimiento de 10 seg. | 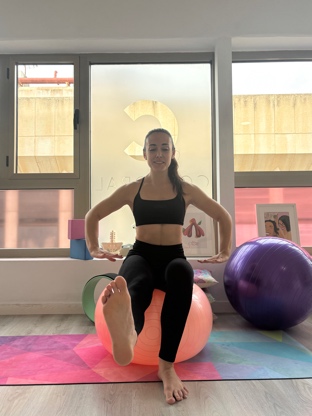 |
| **Báscula pélvica en cuadrupedia.** | En cuadrupedia movimiento de báscula pélvica con activación de transverso y conciencia del suelo pélvico. | 10 repeticiones. | 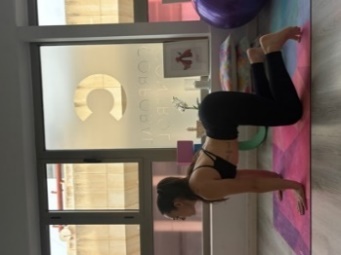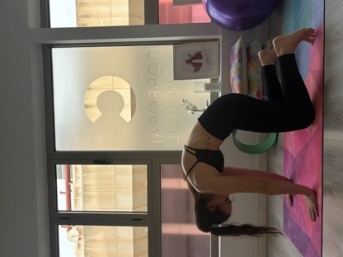 |
| **Elevación miembros contrarios en cuadrupedia.** | En cuadrupedia elevación de un brazo y una pierna contralateral en tiempo espiratorio resistido. Alternar los miembros. | 10 repeticiones. | 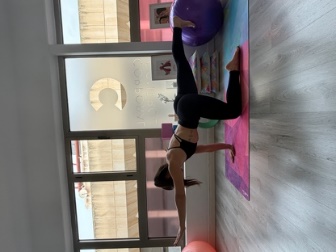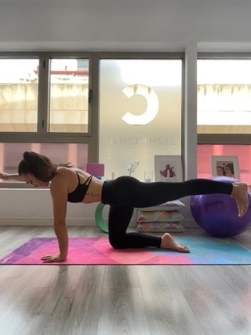 |
| **Hipopresivos y auto elongación en cuadrupedia con flexión de brazos y estiramiento de pierna alternada.** | En cuadrupedia realizar ejercicio hipopresivo (tras 2 respiraciones completas le quedarse en apnea, autoelongación y activación del transverso y suelo pélvico y mantener 10 segundos) con flexión de los brazos y estiramiento de una pierna. Repetir con la otra pierna. | Mantenimiento de 10seg. | 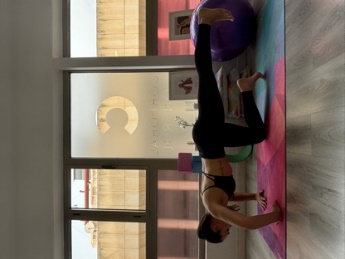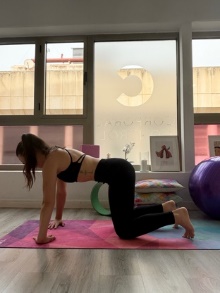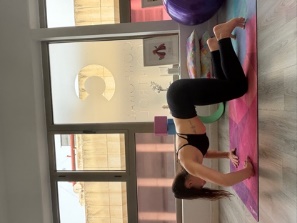 |
| **Postura del Mahometano.** | En cuadrupedia movilización de pelvis y estiramiento dinámico, finalizando con postura del mahometano y estiramiento de la cadena posterior. Sentarse sobre talones, apoyar la cabeza en el suelo y estirar los brazos delante. |  | 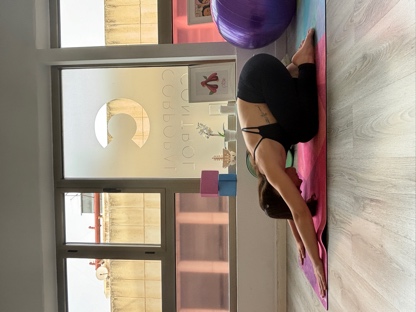 |
| **MES 2, Días 9, 11, 13, 15** | | | |
| **Movilidad de pelvis.** | De pie, ejercicio de conciencia pélvica. Hacer círculos en ambos sentidos e infinitos con la pelvis, manos sobre caderas. | 60 segundos | 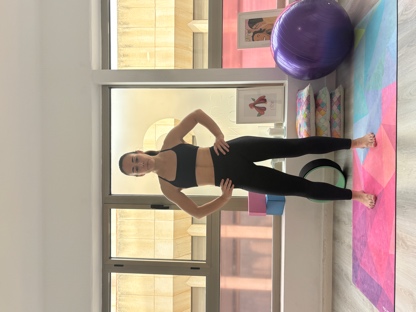 |
| **Contracciones del suelo pélvico.** | De pie, posición de pelvis neutra, contracciones rápidas del suelo pélvico, y luego mantenidas espirando el aire. No quedarse en apnea. | Adaptar número de repeticiones según la valoración que hicimos en consulta. | 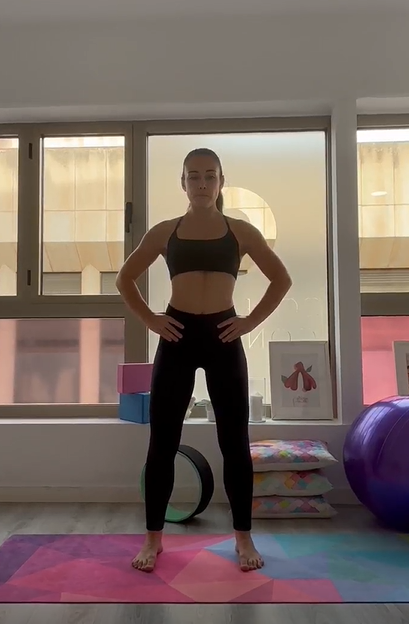 |
| **Equilibrio y control motor sobre bases inestables.** | Ejercicios de equilibrio y control motor, con respiración consciente sobre tronco con base estable, sobre tronco con base inestable y sobre rulo. | Mantener 60 segundos cada postura. | 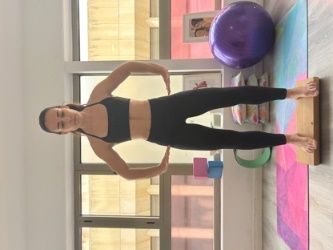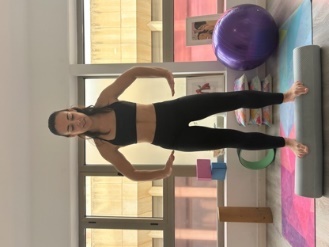 |
| **Sentadillas profundas.** | Pies apoyados a más anchura que las caderas. Sentadilla profunda y subir, activando transverso y suelo pélvico. | 10 repeticiones. | 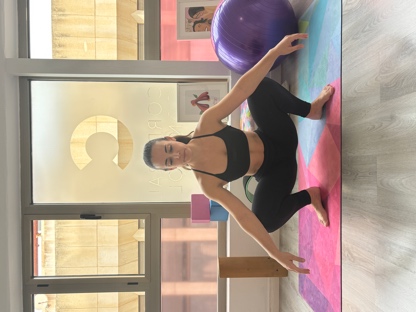 |
| **Sentadillas isométricas** | Pies apoyados a más anchura que las caderas. Sentadilla mantenida 3 segundos y subir, todo activando transverso y suelo pélvico. | 10 repeticiones. | 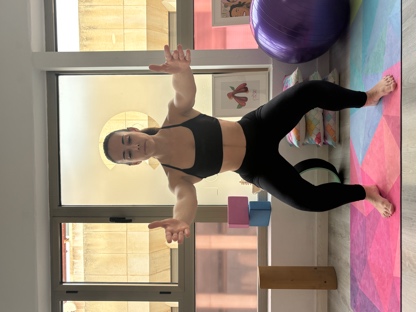 |
| **Zancadas alternadas** | De pie, con brazos extendidos delante. Zancadas hacia delante alternando piernas. Activar transverso y suelo pélvico. | 10 repeticiones. | 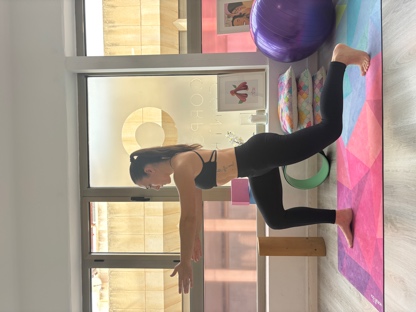 |
| **Auto elongación en suelo.** | Boca arriba, piernas flexionadas con apoyo de talones y flexión dorsal de tobillo. En espiración resistida, autoelongación y activación del transverso y suelo pélvico. | 10 respiraciones. | 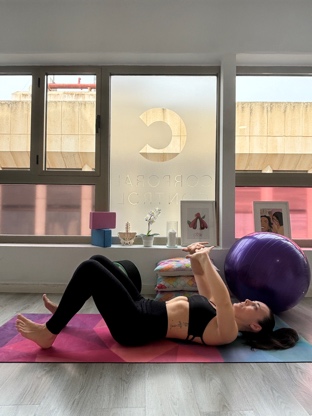 |
| **Puente gluteo.** | Boca arriba, piernas flexionadas con apoyo de talones y flexión dorsal de tobillo. En espiración resistida autoelongación y activación del transverso y suelo pélvico junto con la elevación de glúteos (puente glúteo). | 10 repeticiones. | 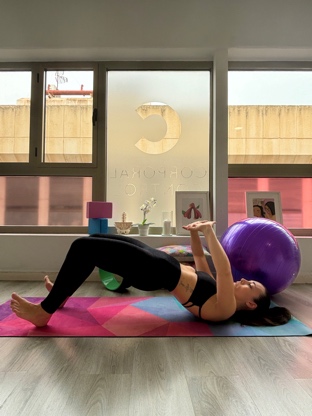 |
| **Puente glúteo con elongación pierna alternada.** | Boca arriba, piernas flexionadas con apoyo de talones. En espiración resistida autoelongación y activación del transverso y suelo pélvico y elevación de glúteos (puente glúteo) con estiramiento de pierna unilateral. Mismo ejercicio con la otra pierna. | 10 repeticiones. | 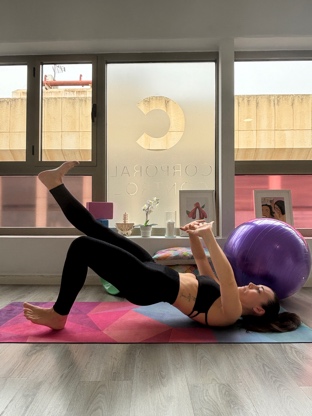 |
| **Activación de aductores con pelota.** | Boca arriba, piernas extendidas a 90° con pelota entre las mismas, se realiza movimiento de flexo extensión de cadera y rodilla con activación de aductores. Ejercicio con respiración normalizada. | 10 repeticiones. | 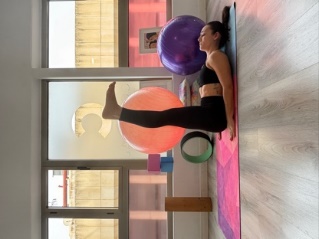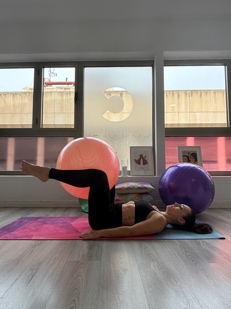 |
| **Abdominales en V alternando piernas flexionadas-estiradas con pelota.** | Boca arriba, piernas a 90° con pelota entre las mismas, se realiza movimiento de flexo-extensión de cadera y rodilla con activación de adductores. Se realiza con espiración en el descenso y estiramiento de las piernas. | 10 repeticiones. | 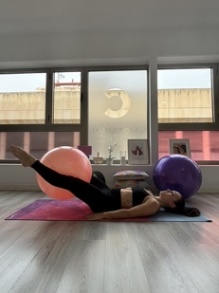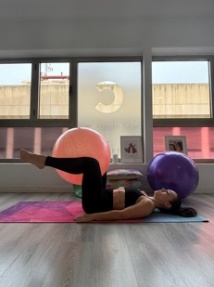 |
| **Estiramiento activo y sesión de relajación y conciencia corporal.** | Estiramientos brazos, piernas, cuello, zona lumbar, de pie, sentada, con calma, fijándose en la respiración.  Vuelta a la calma. | 5 minutos. | 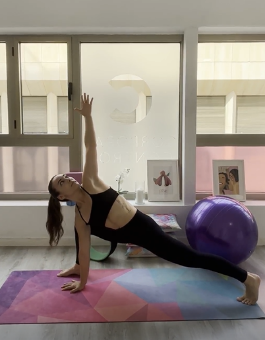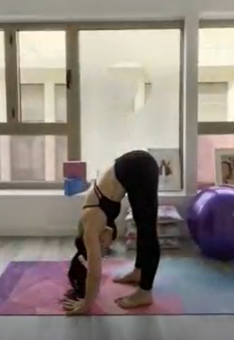  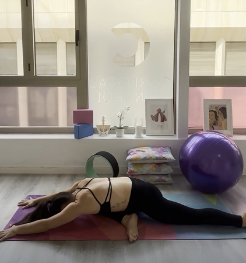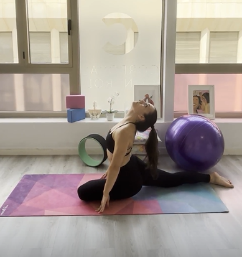 |
| **MES 2, Días 10, 12, 14, 16** | | | |
| **Concientización y propiocepción.** | Boca arriba, ejercicios de conciencia y  propiocepción pélvica, realizando  contracciones mantenidas y rápidas. | Según  valoración inicial del paciente. | 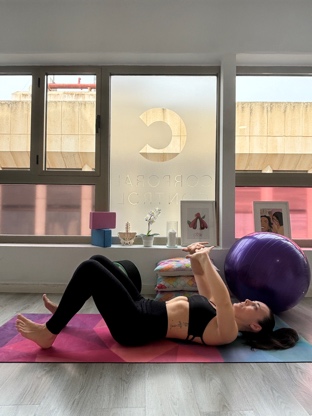 |
| **Auto elongación.** | Boca arriba, piernas flexionadas con apoyo de talones y flexión dorsal de tobillo. En espiración resistida autoelongación, creciendo y activación del transverso y suelo pélvico. | 10 respiraciones. | 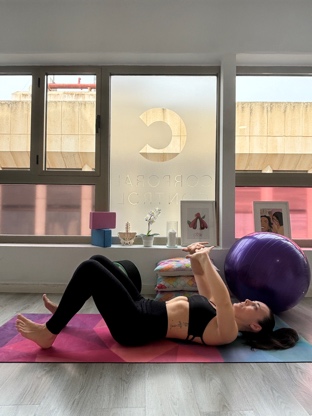 |
| **Puente glúteo.** | Boca arriba, piernas flexionadas con apoyo de talones y flexión dorsal de tobillo. En espiración resistida autoelongación y activación del transverso y suelo pélvico junto con la elevación de glúteos (puente glúteo). | 10 repeticiones. | 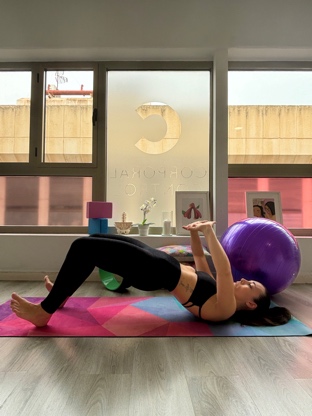 |
| **Puente Glúteo con elongación pierna alternada.** | Boca arriba, piernas flexionadas con apoyo de talones. En espiración resistida autoelongación y activación del transverso y suelo pélvico y elevación de glúteos (puente glúteo) con estiramiento de pierna unilateral. Mismo ejercicio con la otra pierna. | 10 repeticiones. | 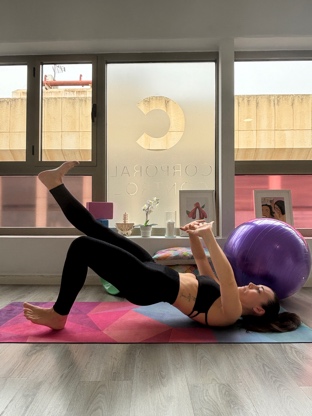 |
| **Elevación de pierna tumbada de lado.** | De lado con piernas estiradas, activación del transverso y del suelo pélvico, realizar movimiento de elevación y descenso de la pierna de arriba con amplitud máxima y velocidad lenta y luego con mínima amplitud del movimiento y máxima velocidad. | 10 repeticiones. | 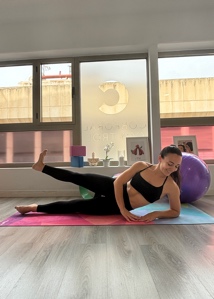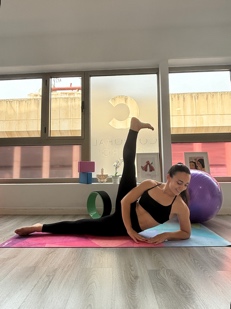 |
| **Circunducción de pierna de lado.** | De lado con piernas estiradas, activación del transverso y conciencia del suelo pélvico, realizar movimiento de círculos con la pierna de arriba en los dos sentidos. | 10 repeticiones. | 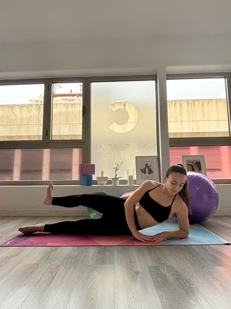 |
| **Flexión y extensión de pierna de lado.** | De lado, piernas alineadas con el tronco, activar el transverso y suelo pélvico, realizar movimiento de flexo extensión de cadera y rodilla. | 10 repeticiones. | 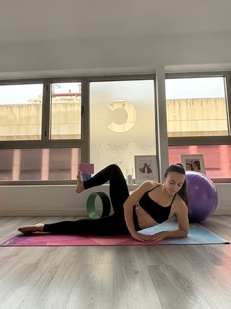 |
| **Hipopresivos y auto elongación en perro invertido.** | En cuadrupedia realizar ejercicio hipopresivo  (tras 2 respiraciones completas, quedar en apnea y activar transverso y suelo pélvico y mantener 10 segundos) con  flexión de brazos y  estiramiento de piernas  (posición de yoga de montaña o perro  invertido). | 10 segundos. | 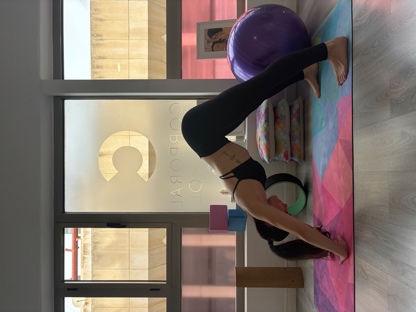 |
| **Hipopresivos y auto elongación en perro invertido con elevación pierna alternada.** | En cuadrupedia realizar ejercicio hipopresivo  (tras 2 respiraciones completas, quedar  en apnea y activar transverso y suelo pélvico y mantener 10 segundos) con  flexión de miembros superiores y  estiramiento de miembros inferiores  (posición de yoga de montaña o perro  invertido) y añadir elevación de una pierna al techo y movimiento de flexo-extensión de cadera y rodilla. Repetir con la otra pierna. | 10 segundos. | 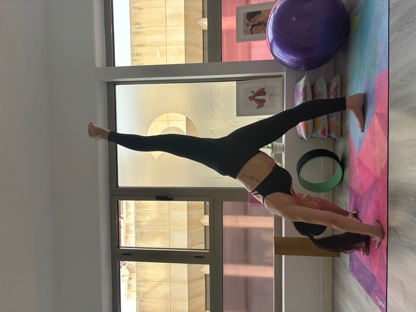 |
| **Plancha frontal con brazos flexionados.** | Plancha frontal sobre la punta de los pies y apoyo sobre antebrazos. Espalda y piernas alineadas, abdominales fuertes. | 60 segundos. | 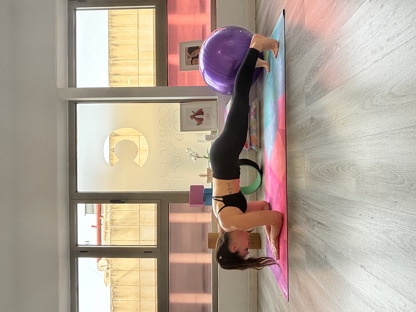 |
| **Plancha frontal con brazos extendidos.** | Plancha frontal sobre la punta de los pies y apoyo sobre manos, brazos extendidos. Espaldas y piernas alineadas. Abdominales fuertes. | 60 segundos. | 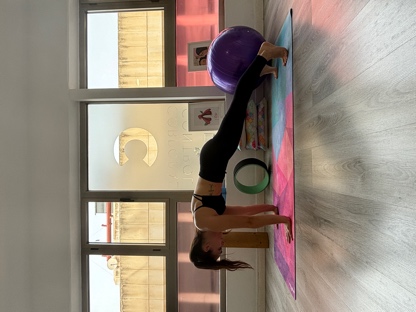 |
| **Planchas laterales.** | Plancha lateral, pies juntos, apoyo sobre uno de los brazos estirado y el otro esta estirado arriba. Mantener pelvis alineado con tronco. Repetir del otro lado. | 60 segundos con 30 segundos de descanso entre cada. | 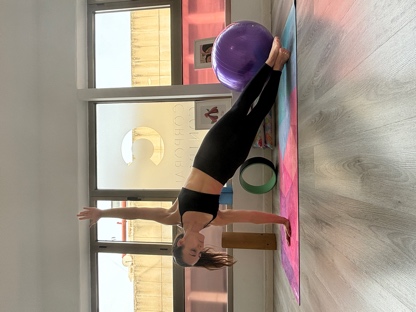 |
| **Plancha frontal con elevación alternada de brazo y pierna.** | Plancha frontal con  elevación alternada de  brazo izquierdo y pierna derecha y sus contrarios con abdominales fuertes y  y conciencia del suelo pélvico. | 10 repeticiones (5 a cada lado). | 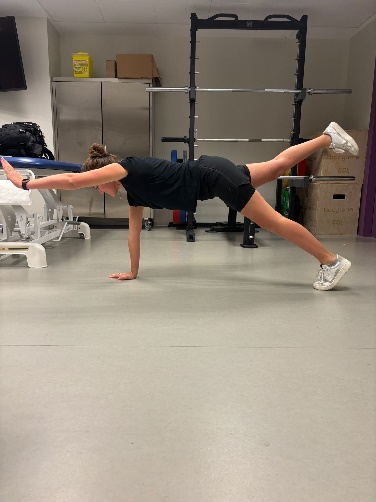 |
| **Plancha frontal – escalador.** | Plancha frontal sobre brazos extendidos. Llevar la rodilla al codo del mismo lado, alternando piernas. | 10 repeticiones. | 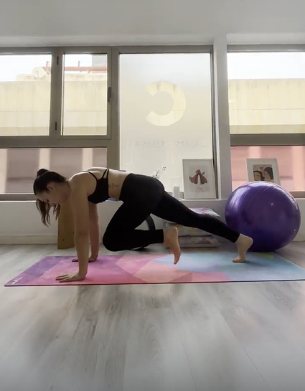 |
| **Crunch en espiración.** | Crunch en espiración, activando abdominales, elevación de la cabeza con las manos detrás ella. | 10 repeticiones. | 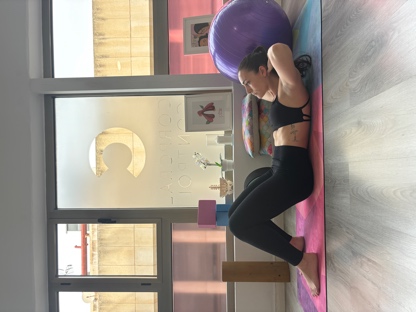 |
| **Crunch en espiración con inclinación lateral.** | Crunch en espiración con inclinación lateral. Manos cruzadas detrás de la cabeza el codo va buscando la rodilla contraria. | 10 repeticiones a cada lado. | 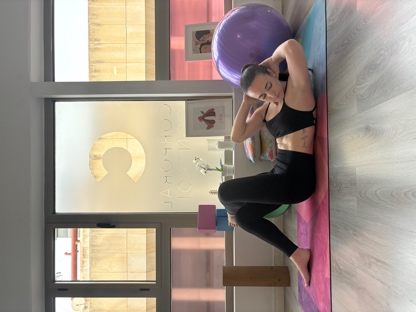 |
| **Crunch con**  **elevación de tronco y lateralidad.** | Piernas dobladas sin apoyar talones. Tronco erguido, contrayendo abdominales, giros de tronco a cada lado. | 10 repeticiones a cada lado. | 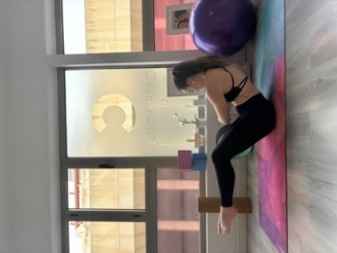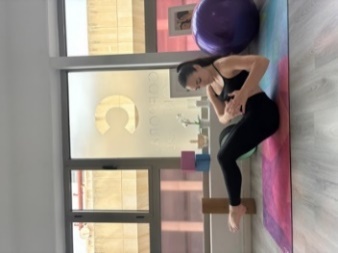 |
| **Oblicuos.** | Boca arriba, piernas flexionadas, apoyando sobre talones. Ligera elevación de cabeza y las manos van buscando cada talón de manera alterna. | 10 repeticiones a cada lado. | 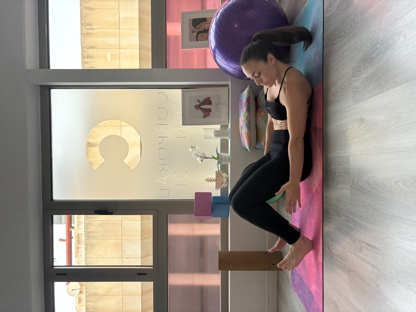 |
| **Estiramiento activo y sesión de relajación y conciencia corporal.** | Estiramientos brazos, piernas, cuello, zona lumbar, de pie, sentada, con calma, fijándose en la respiración. | 5 minutos. | 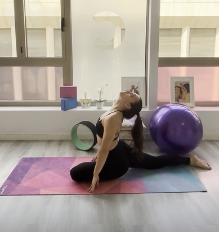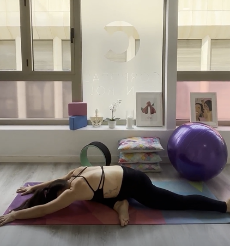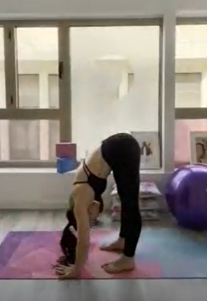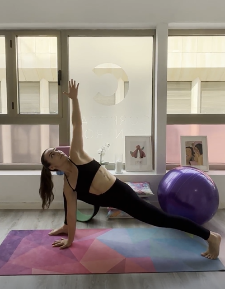 |

Durante este 3r mes, realizaremos ejercicios de movilidad, propiocepción y entrenamiento de la musculatura del suelo pélvico con aumento de la carga a través de la postura y el movimiento. Se introducirá carga externa hasta 60% de su RM de acuerdo con la progresión de las cargas en términos de fuerza, resistencia y salud.

| **MES 3, Días 17, 19, 21, 23** | | | |
| --- | --- | --- | --- |
| **Puente glúteo boca arriba** | Boca arriba, piernas flexionadas con apoyo de talones y flexión dorsal de tobillo. En espiración resistida autoelongación y activación del transverso y suelo pélvico junto con la elevación de glúteos (puente glúteo). | 10 repeticiones. | 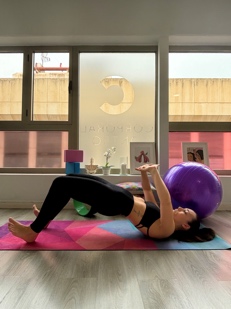 |
| **Puente Glúteo boca arriba con elongación de pierna alternada** | Boca arriba, piernas flexionadas con apoyo de talones. En espiración resistida autoelongación y activación del transverso y suelo pélvico y elevación de glúteos (puente glúteo) con estiramiento de pierna unilateral. Mismo ejercicio con la otra pierna. | 10 repeticiones. | 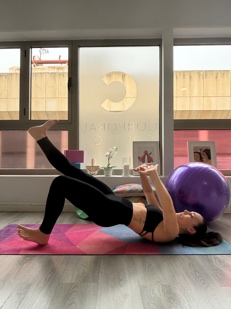 |
| **Abdominales en V alternando piernas flexionadas-estiradas con pelota.** | Boca arriba, piernas a 90° con pelota entre las mismas. Realizar movimiento de flexo-extensión de cadera y rodilla con activación de adductores. Se realiza con espiración en el descenso y estiramiento de las piernas. | 10 repeticiones. | 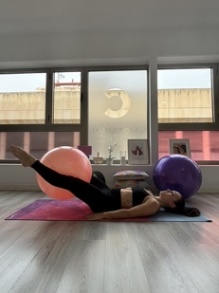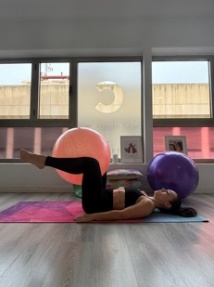 |
| **Plancha frontal con brazos flexionados.** | Plancha frontal sobre la punta de los pies y apoyo sobre antebrazos. Espalda y piernas alineadas, abdominales fuertes. | 90 segundos.  Reposo: 30 seg. | 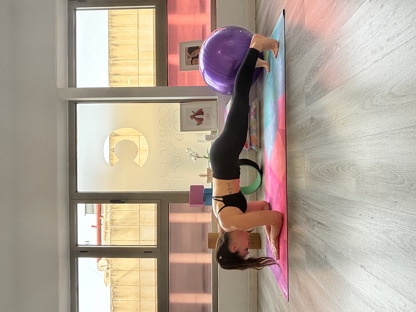 |
| **Plancha frontal con brazos extendidos.** | Plancha frontal sobre la punta de los pies y apoyo sobre manos, brazos extendidos. Espaldas y piernas alineadas. Abdominales fuertes. | 90 segundos.  Reposo: 30seg. | 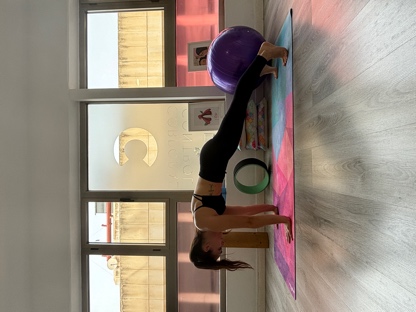 |
| **Plancha lateral.** | Plancha lateral, pies juntos, apoyo sobre uno de los brazos estirado y el otro esta estirado arriba. Repetir del otro lado. | 90 segundos.  Reposo: 30 seg | 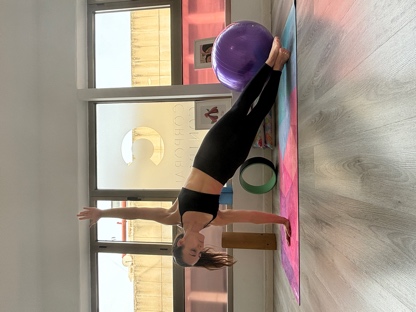 |
| **Plancha frontal con elevación alternada de brazo y pierna.** | Plancha frontal con  elevación alternada de  brazo izquierdo y pierna derecha y sus contrarios con abdominales fuertes y conciencia del suelo pélvico.  Repetir con el otro lado. | 10 repeticiones (5 a cada lado). | 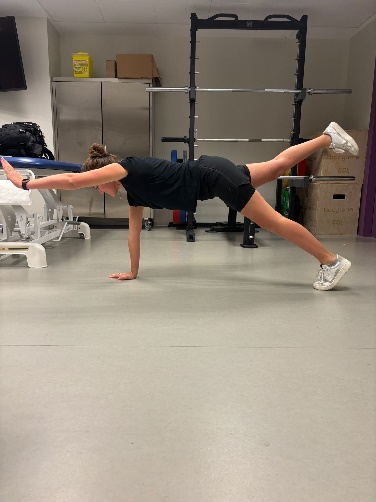 |
| **Plancha frontal – escalador.** | Plancha frontal sobre brazos extendidos. Llevar la rodilla al codo del mismo lado, alternando piernas. | 10 repeticiones. | 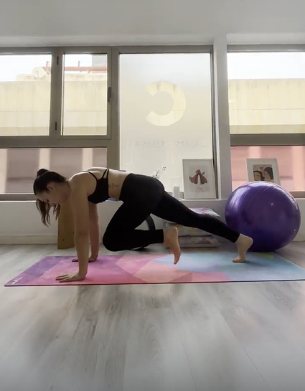 |
| **Estiramiento activo y sesión de relajación y conciencia corporal.** | Estiramientos brazos, piernas, cuello, zona lumbar, de pie, sentada, con calma, fijándose en la respiración.  Vuelta a la calma. | 5 minutos. | 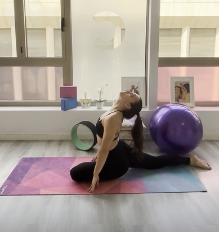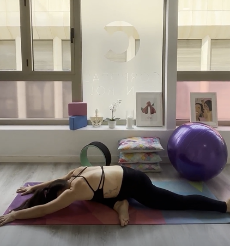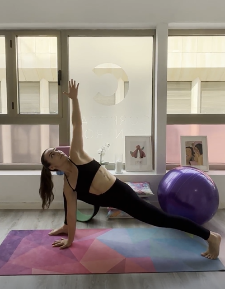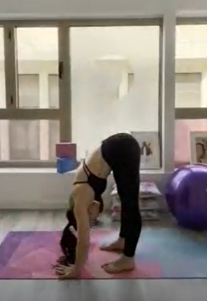 |
| **MES 3, Días 18, 20, 22, 24** | | | |
| **Movilidad de pelvis.** | De pie, ejercicio de conciencia pélvica. Hacer círculos en ambos sentidos e infinitos con la pelvis, manos sobre caderas. | 60 segundos. | 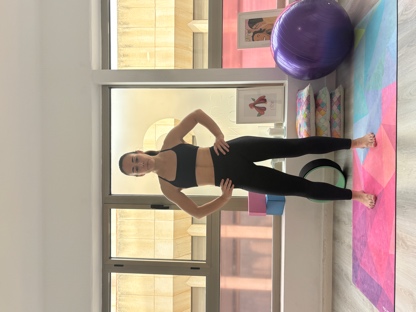 |
| **Contracciones del suelo pélvico.** | De pie, posición de pelvis neutra, contracciones rápidas del suelo pélvico, y luego mantenidas espirando el aire. No quedarse en apnea. | Adaptar número de repeticiones según la valoración que hicimos en consulta. | 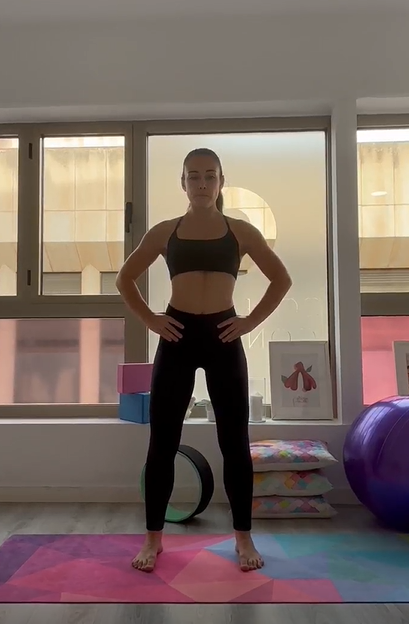 |
| **Equilibrio y control motor sobre tronco con base estable.** | Sobre el tronco, con la base estable en suelo, hacer flexo-extensión de brazos con ketellball y luego rotación de la ketellball alrededor de su abdomen. | 10 repeticiones de cada. | 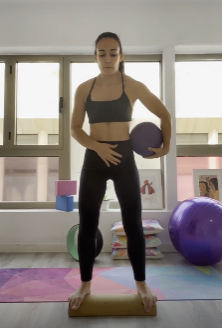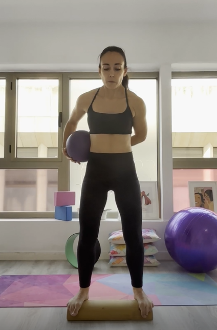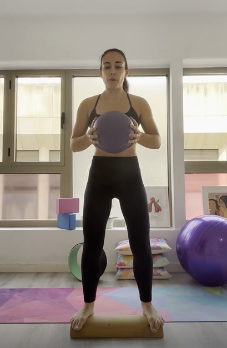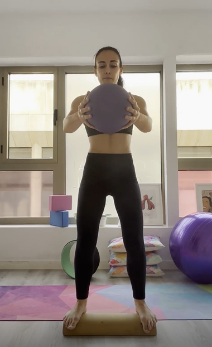 |
| **Equilibrio y control motor sobre tronco con base inestable.** | Sobre el tronco, con la base inestable en suelo, hacer flexo-extensión de brazos con ketellball y luego rotación de la ketellball alrededor de su abdomen. | 10 repeticiones de cada. | 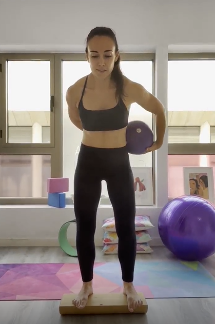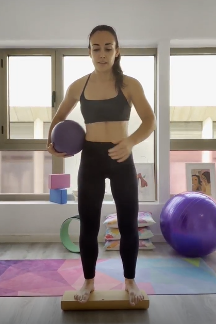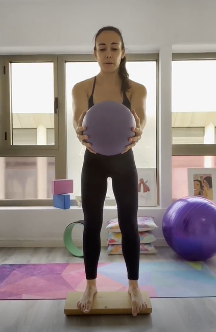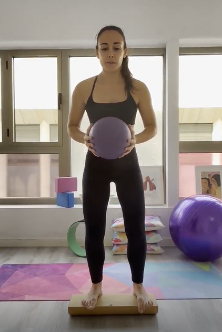 |
| **Equilibrio y control motor sobre rulo.** | Sobre el rulo, hacer flexo-extensión de brazos con ketellball y luego rotación de la ketellball alrededor de su abdomen. | 10 repeticiones de cada. | 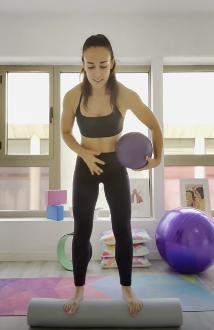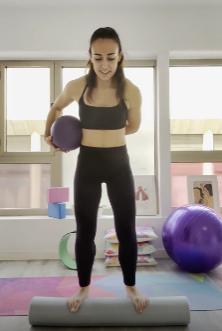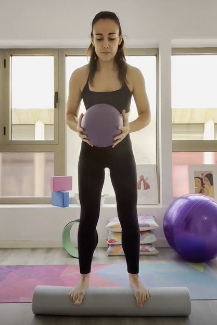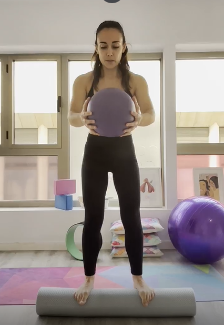 |
| **Sentadilla profunda con ketellball.** | Pies apoyados a más anchura que las caderas. Coger ketellball con brazos extendidos delante. Sentadilla profunda y subir, activando transverso y suelo pélvico. | 10 repeticiones. | 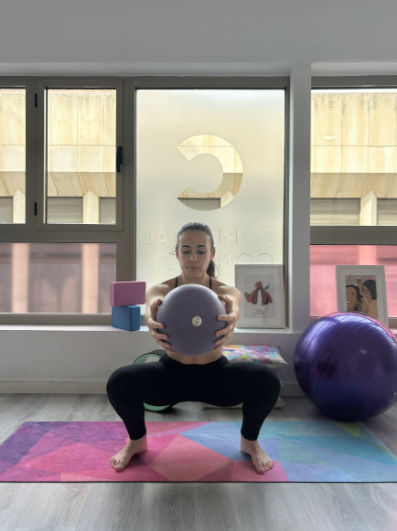 |
| **Sentadilla isométrica con ketellball.** | Pies apoyados a más anchura que las caderas. Coger ketellball con brazos extendidos delante. Sentadilla profunda, mantenida 3 segundos y subir, todo activando transverso y suelo pélvico. | 10 repeticiones. | 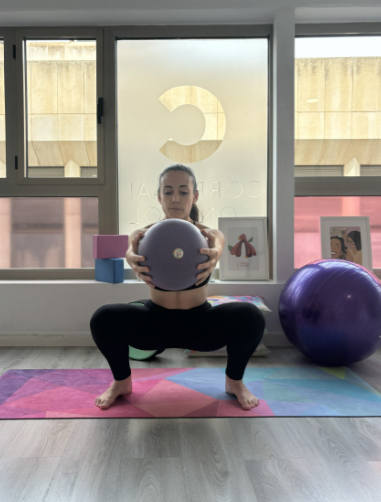 |
| **Zancada alterna con ketellball** | De pie, coger ketellball con brazos extendidos delante. Zancadas hacia delante alternando piernas. Activando transverso y suelo pélvico. | 10 repeticiones de cada pierna. | 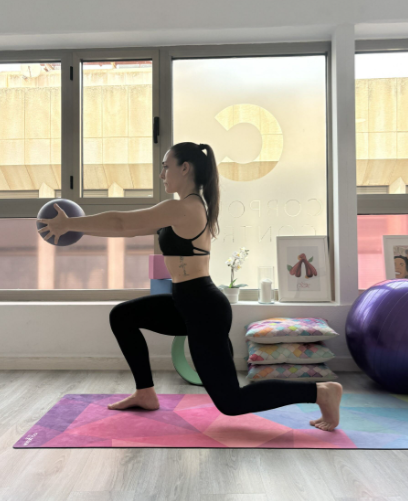 |
| **Hipopresivos y auto elongación en perro invertido.** | En cuadrupedia realizar ejercicio hipopresivo  (tras 2 respiraciones completas, quedar en apnea y activar transverso y suelo pélvico y mantener 10 segundos) con  flexión de miembros superiores y  estiramiento de miembros inferiores  (posición de yoga de montaña o perro  invertido). | 10 segundos. | 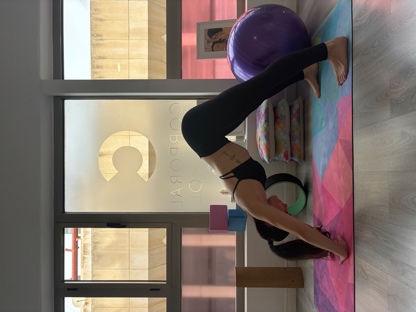 |
| **Hipopresivos y auto elongación en perro invertido con elevación de pierna alternada.** | En cuadrupedia realizar ejercicio hipopresivo  (tras 2 respiraciones completas, quedar  en apnea y activar transverso y suelo pélvico y mantener 10 segundos) con  flexión de miembros superiores y  estiramiento de miembros inferiores  (posición de yoga de montaña o perro  invertido) y añadir elevación de una pierna al techo y movimiento de flexo-extensión de cadera y rodilla. Repetir con la otra pierna. | 10 segundos con cada pierna (20 segundos). | 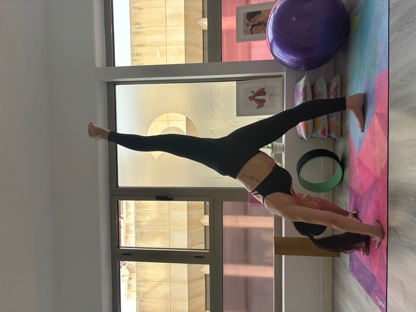 |
| **Crunch con ketellball.** | Boca arriba, apoyo talones, coger la ketellball y elevar de unos grados el tronco activando transverso y suelo pélvico en tiempo espiratorio. | 10 repeticiones. | 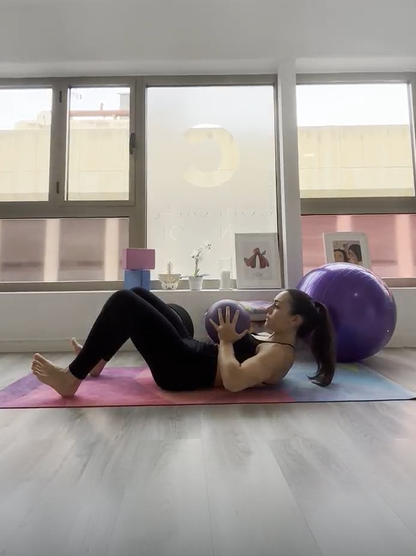 |
| **Crunch con inclinaciones laterales.** | Mismo ejercicio, pero con 2 ketellball (1 en cada mano) o pesas, y en vez de elevar tronco, hacer inclinaciones aproximando la ketellball de cada talón. | 10 repeticiones. | 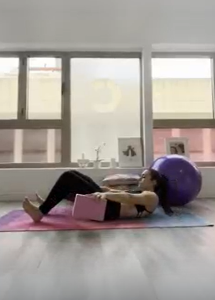 |
| **Crunch con elevación de tronco y lateralidad.** | Misma postura, pero poner la cara lateral del pie izquierdo sobre la rodilla contraria. Elevando el tronco con la ketellball aproximar el hombro derecho de la rodilla izquierda. Repetir del otro lado. | 10 repeticiones. |  |
| **Oblicuos con elevación y pesa.** | Sentada, con inclinación de tronco hacia atrás y sin apoyar piernas, llevar la ketellball a izquierda y derecha alternando. | 10 repeticiones a cada lado. |  |
| **Estiramiento activo y sesión de relajación y conciencia corporal.** | Estiramientos brazos, piernas, cuello, zona lumbar, de pie, sentada, con calma, fijándose en la respiración. | 5 minutos |  |

Durante el 4° mes, volveremos a valorar la RM para la gestión de las cargas. Así, aumentaremos la misma hasta el 75% y nos centraremos en el trabajo y activación del CORE y suelo pélvico de una forma dinámica, con una respiración normalizada e introduciendo el impacto y la fatiga que son factores de riesgo demostrables en la patología pélvico, con el objetivo de automatizar la sinergia abdominopélvica y su competencia en situaciones de la vida diaria, así como del ejercicio físico.

| **MES 4, Días 25, 27, 29,31** |
| --- |

| **Puente glúteo en con elongación pierna alternada.** | Boca arriba, piernas flexionadas con apoyo de talones. En espiración resistida autoelongación y activación del transverso y suelo pélvico y elevación de glúteos (puente glúteo) con estiramiento de pierna unilateral. Mismo ejercicio con la otra pierna. | 10 repeticiones. |  |
| --- | --- | --- | --- |
| **“Hip thrust” con ketellball** | Boca arriba, rodillas flexionadas apoyando los talones en el suelo, levantando punta de los pies hacia el techo, soltar el aire lentamente mientras contraer el abdomen y el suelo pélvico y con el ketellball levantar la pelvis. | 10 repeticiones. |  |
| **Abdominales en V alternando piernas flexionadas-estiradas con pelota.** | Boca arriba, piernas a 90° con pelota entre las mismas, se realiza movimiento de flexo-extensión de cadera y rodilla con activación de adductores. Se realiza con espiración en el descenso y estiramiento de las piernas. | 10 repeticiones. |  |
| **Abdominales en flexión y extensión de cadera y rodilla con pelota.** | Boca arriba, con las rodillas con cadera a 90º y una pelota entre las rodillas apretándola para activar los músculos internos del muslo, lleva los pies hacia el techo y bajada de los pies al suelo sin llegar a tocarlo. | 10 repeticiones. |  |
| **Plancha frontal con brazos flexionados.** | Plancha frontal sobre la punta de los pies y apoyo sobre antebrazos. Espalda y piernas alineadas, abdominales fuertes. | 90 segundos.  Reposo : 30s |  |
| **Plancha frontal con brazos extendidos.** | Plancha frontal sobre la punta de los pies y apoyo sobre manos, brazos extendidos. Espaldas y piernas alineadas. Abdominales fuertes. | 90 segundos.  Reposo : 30s |  |
| **Planchas laterales** | Plancha lateral, pies juntos, apoyo sobre uno de los brazos estirado y el otro esta estirado arriba. Repetir del otro lado. | 60 segundos con 30 segundos de descanso entre cada. |  |
| **Plancha frontal con elevación alternada de brazo y pierna.** | Plancha frontal con  elevación alternada de  brazo izquierdo y pierna derecha y sus contrarios con abdominales fuertes y conciencia del suelo pélvico.  Repetir con el otro lado. | 10 repeticiones (5 a cada lado). |  |
| **Plancha frontal – escalador.** | Plancha frontal sobre brazos extendidos. Llevar la rodilla al codo del mismo lado, alternando piernas. | 10 repeticiones. |  |
| **Hipopresivos y auto elongación en perro invertido con elevación pierna alternada.** | En cuadrupedia realizar ejercicio hipopresivo  (tras 2 respiraciones completas, quedar  en apnea y activar transverso y suelo pélvico y mantener 10 segundos) con  flexión de miembros superiores y  estiramiento de miembros inferiores  (posición de yoga de montaña o perro  invertido) y añadir elevación de una pierna al techo y movimiento de flexo-extensión de cadera y rodilla. Repetir con la otra pierna. | 10 segundos. |  |
| **Estiramiento activo y sesión de relajación y conciencia corporal.** | Estiramientos brazos, piernas, cuello, zona lumbar, de pie, sentada, con calma, fijándose en la respiración. Vuelta a la calma. | 5 minutos. |  |

| **MES 4, Días 26, 28, 30, 32** |
| --- |

| **Movilidad de pelvis y relajación del CORE y suelo pélvico** | De pie, ejercicio de conciencia pélvica. Hacer círculos en ambos sentidos e infinitos con la pelvis, manos sobre caderas. | 60 segundos |  |
| --- | --- | --- | --- |
| **Ejercicio de deambulación con Contracciones Mantenidas** | Caminar a un ritmo cómodo mientras se realizan contracciones mantenidas y rápidas, sin quedarse en apnea. | Cada persona hará una cantidad específica de repeticiones según su evaluación. |  |
| **“Skeeping” con activación de transverso y conciencia del SP** | Levantar las rodillas alternadamente como si estuviéramos corriendo en el lugar, poner especial atención en activar el CORE y en mantener la conciencia del suelo pélvico. | Cada persona hará una cantidad específica de repeticiones según su evaluación. |  |
| **Ejercicios de equilibrio y control motor sobre tronco con ketellball** | Al inicio realizar este ejercicio sobre una base estable, más adelante sobre tronco con base inestable o sobre rulo. Llevar la ketellball entre las piernas y empujar caderas hacia delante y llevar ketellball por encima del pecho, bajar el ketellball de forma controlada. | 10 repeticiones. |  |
| **Sentadilla isométrica con ketellball.** | Pies apoyados a más anchura que las caderas. Coger ketellball con brazos extendidos delante. Sentadilla profunda, mantenida 3 segundos y subir, todo activando transverso y suelo pélvico. | 10 repeticiones. |  |
| **Sentadilla profunda con ketellball.** | Pies apoyados a más anchura que las caderas. Coger ketellball con brazos extendidos delante. Sentadilla profunda y subir, todo activando transverso y suelo pélvico. | 10 repeticiones. |  |
| **Zancada alterna con ketellball** | De pie, coger ketellball con brazos extendidos delante. Zancadas hacia delante alternando piernas. Activando transverso y suelo pélvico. | 10 repeticiones de cada pierna. |  |
| **Burpees** | De pie, con los pies en el ancho de los hombros y los brazos a los lados, activar el transverso abdominal llevando el ombligo hacia adentro y hacia arriba, y contraer a la vez los músculos del suelo pélvico. Desde la posición de pie, bajar en cuclillas y colocar las manos en el suelo, saltar y llevar los pies hacia la posición de plancha, mantener la posición de plancha y volver al inicio. | 10 repeticiones. |  |
| **Salto al cajón** | Pies juntos, saltar sobre un cajón y bajar.  Se puede hacer como subiendo escaleras. Concientizar activación del suelo pélvico. | Repeticiones: 10 |  |
| **Crunch en espiración con ketellball** | Crunch en espiración, activando abdominales, elevación de la cabeza con las manos detrás ella. | 10 repeticiones. |  |
| **Crunch con inclinaciones laterales.** | Mismo ejercicio, pero con 2 ketellball (1 en cada mano) o pesas, ligera flexion de cabeza, y en vez de elevar tronco, hacer inclinaciones aproximando la ketellball a cada talón. | 10 repeticiones. |  |
| **Crunch con elevación de tronco y lateralidad.** | Misma postura, pero poner la cara lateral del pie izquierdo sobre la rodilla contraria. Elevando el tronco con la ketellball aproximar el hombro derecho de la rodilla izquierda. Repetir del otro lado. | 10 repeticiones. |  |
| **Oblicuos con elevación y pesa.** | Sentada, con inclinación de tronco hacia atrás y sin apoyar piernas, llevar la ketellball a izquierda y derecha alternando. | 10 repeticiones a cada lado. |  |
| **Estiramiento activo y sesión de relajación y conciencia corporal.** | Estiramientos brazos, piernas, cuello, zona lumbar, de pie, sentada, con calma, fijándose en la respiración.  Vuelta a la calma. | 5 minutos. |  |

**ANEXO 5. HOJA DE REGISTRO DE FUERZA MUSCULAR**

Código de identificación de la paciente:

| Valoracion manual | | |
| --- | --- | --- |
|  |  |  |
| Balance Manual fuerza (oxford) | | |
|  | pre-intervención | post-intevención |
| 1 |  |  |
| 2 |  |  |
| 3 |  |  |
| Resistencia | | |
| *"manten la contracción" contabilizar los segundos* | | |
|  | pre-intervención | post-intevención |
| 1 |  |  |
| 2 |  |  |
| 3 |  |  |
| Fatigabilidad | | |
| *"contrae tantas veces como sea posible"* | | |
| *6 seg contracción-6s relajación, nº de repeticiones hasta que no pueda mantener contrac* | | |
|  | pre-intervención | post-intevención |
| 1 |  |  |
| 2 |  |  |
| 3 |  |  |

| Valoración pelvimetro phenix | | |
| --- | --- | --- |
|  |  |  |
| Fuerza máxima | | |
| *"contrae tan fuerte como puedas durante 10segundos" pico máximo* | | |
|  | pre-intervención | post-intevención |
| 1 |  |  |
| 2 |  |  |
| 3 |  |  |

**ANEXO 6. CUESTIONARIOS DE FUNCIÓN PÉLVICA Y CALIDAD DE VIDA**

- **PFIQ-20 (PELVIC FLOOR IMPACT QUESTIONNARIE Short Form)**

Este cuestionario versa sobre ciertos síntomas intestinales, urinarios o pélvico; se le preguntará si usted siente estos síntomas y, si los siente, lo mucho que le molestan. Por favor, responda las preguntas a continuación marcando con una (X) en la casilla o casillas correspondiente (s). Si usted duda sobre alguna respuesta, selecciones la que mejor se adapte a su caso. Al responder a este cuestionario, tenga en cuenta los síntomas que ha sentido en los últimos 3 meses.

Por favor conteste a todas las preguntas de la siguiente encuesta:

1. **Habitualmente, ¿siente usted presión en la parte baja del abdomen?**

No ; Sí

Si la respuesta es sí, ¿cuánto le molesta?

| 1 | 2 | 3 | 4 |
| --- | --- | --- | --- |
| Nada | Un poco | Moderadamente | Mucho |

1. **Habitualmente, ¿tiene usted una sensación de pesadez en la zona de la pelvis?**

No ; Sí

Si la respuesta es sí, ¿cuánto le molesta?

| 1 | 2 | 3 | 4 |
| --- | --- | --- | --- |
| Nada | Un poco | Moderadamente | Mucho |

1. **Habitualmente, ¿siente usted un “abultamiento” o algo que sale hacia fuera que puede tocar o ver en la zona de su vagina?**

No ; Si

Si la respuesta es sí, ¿cuánto le molesta?

| 1 | 2 | 3 | 4 |
| --- | --- | --- | --- |
| Nada | Un poco | Moderadamente | Mucho |

1. **¿Alguna vez tiene que empujar sobre su vagina o alrededor de su recto para poder defecar o para acabar de defecar?**

No ; Sí

Si la respuesta es sí, ¿cuánto le molesta?

| 1 | 2 | 3 | 4 |
| --- | --- | --- | --- |
| Nada | Un poco | Moderadamente | Mucho |
|  |  |  |  |

1. **Habitualmente, ¿tiene usted una sensación de no llegar a vaciar completamente su vejiga?**

No ; Sí

Si la respuesta es sí, ¿cuánto le molesta?

| 1 | 2 | 3 | 4 |
| --- | --- | --- | --- |
| Nada | Un poco | Moderadamente | Mucho |

1. **¿Alguna vez tiene usted que empujar con los dedos un “abultamiento” en la zona de la vagina para iniciar o completar la micción?**

No ; Sí

Si la respuesta es sí, ¿cuánto le molesta?

| 1 | 2 | 3 | 4 |
| --- | --- | --- | --- |
| Nada | Un poco | Moderadamente | Mucho |

1. **¿Tiene usted la sensación de tener que empujar mucho para poder defecar?**

No ; Sí

Si la respuesta es sí, ¿cuánto le molesta?

| 1 | 2 | 3 | 4 |
| --- | --- | --- | --- |
| Nada | Un poco | Moderadamente | Mucho |

1. **¿Tiene usted la sensación de que no ha vaciado completamente el intestino tras haber defecado?**

No ; Sí

Si la respuesta es sí, ¿cuánto le molesta?

| 1 | 2 | 3 | 4 |
| --- | --- | --- | --- |
| Nada | Un poco | Moderadamente | Mucho |

1. **Habitualmente, ¿tiene usted pérdidas fecales involuntarias cuando sus heces son sólidas?**

No ; Sí

Si la respuesta es sí, ¿cuánto le molesta?

| 1 | 2 | 3 | 4 |
| --- | --- | --- | --- |
| Nada | Un poco | Moderadamente | Mucho |

1. **Habitualmente, ¿tiene usted pérdidas fecales involuntarias cuando sus heces son muy blandas o liquidas?**

No ; Sí

Si la respuesta es sí, ¿cuánto le molesta?

| 1 | 2 | 3 | 4 |
| --- | --- | --- | --- |
| Nada | Un poco | Moderadamente | Mucho |

1. **Habitualmente, ¿tiene usted pérdida involuntaria de gases (pedos)?**

No ; Sí

Si la respuesta es sí, ¿cuánto le molesta?

| 1 | 2 | 3 | 4 |
| --- | --- | --- | --- |
| Nada | Un poco | Moderadamente | Mucho |

1. **Habitualmente, ¿siente usted dolor al defecar?**

No ; Sí

Si la respuesta es sí, ¿cuánto le molesta?

| 1 | 2 | 3 | 4 |
| --- | --- | --- | --- |
| Nada | Un poco | Moderadamente | Mucho |

1. **Habitualmente, ¿tiene usted una fuerte sensación de urgencia de forma que tiene que correr al baño para defecar?**

No ; Sí

Si la respuesta es sí, ¿cuánto le molesta?

| 1 | 2 | 3 | 4 |
| --- | --- | --- | --- |
| Nada | Un poco | Moderadamente | Mucho |

1. **¿Alguna vez parte de su intestino sobresale por el ano mientras usted defeca o al acabar de defecar?**

No ; Sí

Si la respuesta es sí, ¿cuánto le molesta?

| 1 | 2 | 3 | 4 |
| --- | --- | --- | --- |
| Nada | Un poco | Moderadamente | Mucho |

1. **Habitualmente, ¿orina con frecuencia?**

No ; Sí

Si la respuesta es sí, ¿cuánto le molesta?

| 1 | 2 | 3 | 4 |
| --- | --- | --- | --- |
| Nada | Un poco | Moderadamente | Mucho |

1. **Habitualmente ¿experimenta usted pérdidas involuntarias de orina asociadas a una sensación de urgencia, es decir, una fuerte sensación de tener que ir al baño a orinar?**

No ; Sí

Si la respuesta es sí, ¿cuánto le molesta?

| 1 | 2 | 3 | 4 |
| --- | --- | --- | --- |
| Nada | Un poco | Moderadamente | Mucho |

1. **Habitualmente, ¿tiene usted pérdidas de orina al toser, estornudar o reír?**

No ; Sí

Si la respuesta es sí, ¿cuánto le molesta?

| 1 | 2 | 3 | 4 |
| --- | --- | --- | --- |
| Nada | Un poco | Moderadamente | Mucho |

1. **Habitualmente, ¿tiene usted la sensación de perder pequeñas cantidades de orina (es decir, gotas)?**

No ; Sí

Si la respuesta es sí, ¿cuánto le molesta?

| 1 | 2 | 3 | 4 |
| --- | --- | --- | --- |
| Nada | Un poco | Moderadamente | Mucho |

1. **Habitualmente, ¿tiene usted dificultades para vaciar su vejiga?**

No ; Sí

Si la respuesta es sí, ¿cuánto le molesta?

| 1 | 2 | 3 | 4 |
| --- | --- | --- | --- |
| Nada | Un poco | Moderadamente | Mucho |
|  |  |  |  |

1. **Habitualmente, ¿siente usted dolor o molestia en la parte baja del abdomen o en la zona genital?**

No ; Sí

Si la respuesta es sí, ¿cuánto le molesta?

| 1 | 2 | 3 | 4 |
| --- | --- | --- | --- |
| Nada | Un poco | Moderadamente | Mucho |

- **ICIQ-SF (International Consultation on Incontinence Questionnarie – Short Form)**

Hay mucha gente que en un momento determinado pierde orina. Estamos intentando determinar el número de personas que presentan este problema y hasta qué punto les preocupa esta situación. Le estaríamos muy agradecidos si nos contestase las siguientes preguntas, pensando en cómo se ha encontrado en las ULTIMAS CUATRO SEMANAS.

1.¿Con qué frecuencia pierde orina? (Marque una).

___ 1 nunca

___ 2 una vez a la semana o menos

___ 3 dos o tres veces a la semana

___ 4 una vez al día

___ 5 varias veces al día

___ 6 continuamente

2.Nos gustaría saber su impresión acerca de la cantidad de orina que usted cree que se le escapa. Cantidad de orina que pierde habitualmente (tanto si lleva protección como si no) (Marque sólo una opción)

___ 1 no se me escapa nada

___ 2 muy poca cantidad

___ 3 una cantidad moderada

___ 4 mucha cantidad.

3.Estos escapes de orina que tiene ¿cuánto afectan su vida diaria?

Por favor marque una cruz, en la siguiente lista, un número entre 0 (no me afectan nada) y 10 (me afectan mucho)

1. 1 2 3 4 5 6 7 8 9 10

nada mucho

Puntuación de ICI-Q: sume las puntuaciones de las preguntas 3+4+5:____

4.¿Cuándo pierde orina? (Señale todo lo que le pasa a usted)

___ 1 nunca pierde orina

___ 2 pierde orina antes de llegar al WC

___ 3 pierde orina cuando tosa o estornuda

___ 4 pierde cuando duerme

___ 5 pierde orina cuando hace esfuerzos físicos/ejercicio

___ 6 pierde orina al acabar de orinar y ya se ha vestido

___ 7 pierde orina sin un motivo evidente

___ 8 pierde orina de forma continua

- **Test de Severidad de Sandvick**

Este test evalúa la gravedad de los síntomas de incontinencia de orina en la mujer.

1.¿Con qué frecuencia pierde orina? (Marque una).

___ 1 Menos de una vez al mes

___ 2 Algunas veces al mes

___ 3 Algunas veces a la semana

___ 4 Todos los días y/o noches

2.¿Qué cantidad de orina se le escapa cada vez? (Marque una).

___ 1 Gotas (muy poca cantidad)

___ 2 Chorro pequeño (una cantidad moderada)

___ 3 Mucha cantidad

**ANEXO 7. CUESTIONARIOS DE SÍNTOMAS VAGINALES**

- **VHI (Índice de Salud Vaginal)**

**Señale la respuesta que indique el estado de los distintos aspectos vaginales que presenta:**

- - - 1. **Elasticidad**
         1. **Ausente**
         2. **Escasa**
         3. **Aceptable**
         4. **Buena**
         5. **Excelente**
      2. **Volumen de flujo**
         1. **Ausente**
         2. **Escaso**
         3. **Capa fina en la superficie**
         4. **Capa moderada**
         5. **Normal**
      3. **PH**
         1. **>6,1**
         2. **5,6 a 6**
         3. **5,5 a 5**
         4. **4,7 a 6**
         5. **<4,6**
      4. **Integridad del epitelio**
         1. **Petequias espontáneas**
         2. **Sangra al menor contacto**
         3. **Sangra al rascado**
         4. **Epitelio no friable**
         5. **Normal**
      5. **Humedad**
         1. **Ausente. Superficie inflamada**
         2. **Ausente. Superficie normal**
         3. **Mínima**
         4. **Moderada**
         5. **Normal**

**ANEXO 8. CUESTIONARIOS DE FUNCIÓN SEXUAL Y AUTOESTIMA**

- **FSFI (Índice de Función Sexual Femenina)**

Las siguientes preguntas son sobre sus sentimientos y respuestas sexuales durante las últimas 4 semanas. Por favor, conteste a las siguientes preguntas lo más honesta y claramente posible. Sus respuestas serán completamente confidenciales.

MARQUE UNA SOLA RESPUESTA A CADA PREGUNTA:

Deseo o interés sexual: es la sensación que incluye el deseo de tener una experiencia sexual, sentirse receptiva a la iniciación sexual de la pareja y pensar o fantasear sobre tener sexo.

**1. En las últimas 4 semanas, ¿con qué frecuencia experimentó deseo o interés sexual?**

 Casi siempre o siempre

 La mayoría de las veces (más de la mitad de las veces)

 A veces (aproximadamente la mitad de las veces)

 Pocas veces (menos de la mitad de las veces)

 Casi nunca o nunca

**2. En las últimas 4 semanas, ¿cómo calificaría su nivel (grado) de deseo o interés sexual?**

 Muy alto

 Alto

 Moderado

 Bajo

 Muy bajo o nada

Excitación sexual: es la sensación que incluye aspectos físicos y mentales de la exaltación sexual. Puede incluir sensación de calor o latidos en los genitales, lubricación (humedad) o contracciones musculares.

**3. En las últimas 4 semanas, ¿con qué frecuencia sintió excitación sexual durante la actividad sexual o coito vaginal?**

 Sin actividad sexual

 Casi siempre o siempre

 La mayoría de las veces (más de la mitad de las veces)

 A veces (aproximadamente la mitad de las veces)

 Pocas veces (menos de la mitad de las veces)

 Casi nunca o nunca

**4. En las últimas 4 semanas, ¿cómo calificaría su nivel de excitación sexual durante la actividad sexual o coito vaginal?**

 Sin actividad sexual

 Muy alto

 Alto

 Moderado

 Bajo

 Muy bajo o nada

**5. En las últimas 4 semanas, ¿Cuánta confianza tuvo para conseguir excitarse durante la actividad sexual o coito vaginal?**

 Sin actividad sexual

 Confianza muy alta

 Confianza alta

 Confianza moderada

 Confianza baja

 Confianza muy baja o nada

**6. En las últimas 4 semanas, ¿con qué frecuencia se sintió satisfecha con su excitación durante la actividad sexual o coito vaginal?**

 Sin actividad sexual

 Casi siempre o siempre

 La mayoría de las veces (más de la mitad de las veces)

 A veces (aproximadamente la mitad de las veces)

 Pocas veces (menos de la mitad de las veces)

 Casi nunca o nunca

**7. En las últimas 4 semanas, ¿con qué frecuencia consiguió la lubricación vaginal (humedad vaginal) durante la actividad sexual o coito vaginal?**

 Sin actividad sexual

 Casi siempre o siempre

 La mayoría de las veces (más de la mitad de las veces)

 A veces (aproximadamente la mitad de las veces)

 Pocas veces (menos de la mitad de las veces)

 Casi nunca o nunca

**8. En las últimas 4 semanas, ¿cuánta dificultad encontró para lubricarse (humedad vaginal) durante la actividad sexual o coito vaginal?**

 Sin actividad sexual

 Extremadamente difícil o imposible

 Muy difícil

 Difícil

 Un poco difícil

 Sin dificultad

**9. En las últimas 4 semanas, ¿con qué frecuencia mantuvo la lubricación vaginal (humedad vaginal) hasta finalizar la actividad sexual o coito vaginal?**

 Sin actividad sexual

 Casi siempre o siempre

 La mayoría de las veces (más de la mitad de las veces)

 A veces (aproximadamente la mitad de las veces)

 Pocas veces (menos de la mitad de las veces)

 Casi nunca o nunca

**10. En las últimas 4 semanas, ¿cuánta dificultad encontró para mantener la lubricación vaginal (humedad vaginal) hasta finalizar la actividad sexual o coito vaginal?**

 Sin actividad sexual

 Extremadamente difícil o imposible

 Muy difícil

 Difícil

 Un poco difícil

 Sin dificultad

**11. En las últimas 4 semanas, cuando tuvo una estimulación sexual o coito vaginal, ¿con qué frecuencia consiguió el orgasmo (clímax)?**

 Sin actividad sexual

 Casi siempre o siempre

 La mayoría de las veces (más de la mitad de las veces)

 A veces (aproximadamente la mitad de las veces)

 Pocas veces (menos de la mitad de las veces)

 Casi nunca o nunca

**12. En las últimas 4 semanas, cuando tuvo una estimulación sexual o coito vaginal, ¿cuánta dificultad tuvo para alcanzar el orgasmo (clímax)?**

 Sin actividad sexual

 Extremadamente difícil o imposible

 Muy difícil

 Difícil

 Un poco difícil

 Sin dificultad

**13. En las últimas 4 semanas, ¿cuánta satisfacción sintió con su capacidad para alcanzar el orgasmo (clímax) durante la actividad sexual o coito vaginal??**

 Sin actividad sexual

 Muy satisfecha

 Moderadamente satisfecha

 Ni satisfecha ni insatisfecha

 Moderadamente insatisfecha

 Muy insatisfecha

**14. En las últimas 4 semanas, ¿cuánta satisfacción sintió con el acercamiento emocional con su pareja durante la actividad sexual?**

 Sin actividad sexual

 Muy satisfecha

 Moderadamente satisfecha

 Ni satisfecha ni insatisfecha

 Moderadamente insatisfecha

 Muy insatisfecha

**15. En las últimas 4 semanas, ¿cuánta satisfacción sintió con la relación sexual con su pareja?**

 Muy satisfecha

 Moderadamente satisfecha

 Ni satisfecha ni insatisfecha

 Moderadamente insatisfecha

 Muy insatisfecha

**16. En las últimas 4 semanas, ¿cuánta satisfacción sintió con su vida sexual en general?**

 Muy satisfecha

 Moderadamente satisfecha

 Ni satisfecha ni insatisfecha

 Moderadamente insatisfecha

 Muy insatisfecha

**17. En las últimas 4 semanas, ¿con cuánta frecuencia sintió molestias o dolor durante coito vaginal?**

 Sin coito vaginal

 Casi siempre o siempre

 La mayoría de las veces (más de la mitad de las veces)

 A veces (aproximadamente la mitad de las veces)

 Pocas veces (menos de la mitad de las veces)

 Casi nunca o nunca

**18. En las últimas 4 semanas, ¿con cuánta frecuencia sintió molestias o dolor después del coito vaginal?**

 Sin coito vaginal

 Casi siempre o siempre

 La mayoría de las veces (más de la mitad de las veces)

 A veces (aproximadamente la mitad de las veces)

 Pocas veces (menos de la mitad de las veces)

 Casi nunca o nunca

**19. En las últimas 4 semanas, ¿cómo valoraría su nivel (grado) de dolor o molestias durante o después**

**del coito vaginal?**

 Sin coito vaginal

 Muy alto

 Alto

 Moderado

 Bajo

 Muy bajo o nada

- **EVA (Escala visual analógica)**

Marque en la línea el punto que indique la intensidad del dolor durante las relaciones sexuales. La valoración será: 1 Dolor leve si el paciente puntúa el dolor como menor de 3; 2 Dolor moderado si la valoración se sitúa entre 4 y 7; 3 Dolor severo si la valoración es igual o superior a 8.

1. 1 2 3 4 5 6 7 8 9 10

- **S-BIS (Escala de imagen corporal)**

En este cuestionario se le preguntará sobre cómo se siente acerca de su aspecto físico y de los cambios que puedan haberle ocurrido como consecuencia de la enfermedad o del tratamiento recibido

Por favor, lea cuidadosamente cada ítem y marque la respuesta que mejor se ajuste a cómo se ha sentido durante la semana pasada.

**1.¿Se ha sentido avergonzada(o) o cohibida(o) por su aspecto físico?**

**a. En absoluto**

**b. Un poco**

**c. Bastante**

**d. Mucho**

**2.¿Se ha sentido físicamente menos atractiva(o) a consecuencia de la enfermedad o de su tratamiento?**

**a. En absoluto**

**b. Un poco**

**c. Bastante**

**d. Mucho**

**3.¿Se ha sentido descontenta(o) con su aspecto cuando está vestida(o)?**

**a. En absoluto**

**b. Un poco**

**c. Bastante**

**d. Mucho**

**4.¿Se ha sentido menos femenina/masculino como consecuencia de su enfermedad o tratamiento?**

**a. En absoluto**

**b. Un poco**

**c. Bastante**

**d. Mucho**

**5.¿Le resulta difícil mirarse cuando está desnuda(o)?**

**a. En absoluto**

**b. Un poco**

**c. Bastante**

**d. Mucho**

**6.¿Se ha sentido menos atractiva(o) sexualmente a consecuencia de su enfermedad o tratamiento?**

**a. En absoluto**

**b. Un poco**

**c. Bastante**

**d. Mucho**

**7.¿Ha evitado a alguien debido al modo en que se sentía en relación a su aspecto?**

**a. En absoluto**

**b. Un poco**

**c. Bastante**

**d. Mucho**

**8.¿Ha sentido que el tratamiento ha dejado su cuerpo “menos completo”?**

**a. En absoluto**

**b. Un poco**

**c. Bastante**

**d. Mucho**

**8.¿Se ha sentido insatisfecha(o) con su cuerpo?**

**a. En absoluto**

**b. Un poco**

**c. Bastante**

**d. Mucho**

**8.¿Se ha sentido insatisfecha(o) con el aspecto de su cicatriz?**

**a. En absoluto**

**b. Un poco**

**c. Bastante**

**d. Mucho**

- **Escala de satisfaccion con el tratamiento**

¿Cómo de satisfecho está con el tratamiento recibido?

Totalmente insatisfecho

Insatisfecho

Algo insatisfecho

Satisfecho

Totalmente satisfecho
