# Supplementary material for: Effectiveness of radiofrequency and exercise-based rehabilitation on symptoms associated with pelvic floor dysfunction in breast cancer patients: A study protocol
Source: PLoS One. 2025 Aug 29;20(8):e0330156. doi: 10.1371/journal.pone.0330156 (PMC12396660; doi:10.1371/journal.pone.0330156)
Supplement: Attachment — Submitted filename: Response to Reviewers.docx [file pone.0330156.s006.docx]

**Title:** Effectiveness of radiofrequency and exercise-based rehabilitation on symptoms associated with pelvic floor dysfunction in breast cancer patients: A study protocol.

**Article Type:** Study protocol

We wish to thank you greatly for the interest you have shown towards our study, for taking the time to review it and for your constructive comments.

All changes are indicated in red in the manuscript and in blue here.

Here follows a point-by-point response to your comments:

**Reviewer Comments:**

**Reviewer #1:** **The authors present a protocol for a triple arm randomised clinical trial to improve the quality of life of breast cancer patients. The research objectives are good but the protocol needs some methodological/statistical improvement.**

**1. Abstract: Methods: All clinical trials are prospective and hence the word prospective is not needed.**

The word ‘prospective’ has been removed from the abstract and from the Trial Design section of the Materials and Methods. Thank you.

**2. Sample size needs to be calculated and presented based on the assumed minimum clinically worthwhile effect size in terms of primary end point. Also, the test statistics behind the sample size and the software used for calculation needs to be mentioned.**

The information requested by the reviewer has been included in the section “Study population” of the Materials and Methods (page 7) as follows:

For sample calculation, the minimum clinically significant effect size of the PFDI-20 was 30 points, with a Cohen's d of 0.612 between extreme groups, assuming a standard deviation of 50, with a 30% loss to follow-up, an alpha risk (α) of 5% and a power (1-β) of 80%. A sample size of 117 women (39 in each group) was obtained using the G*Power program.

**3. Primary outcome and secondary outcomes are not clearly defined. Primary outcome and secondary outcomes need to be clearly defined with their corresponding endpoints.**

We appreciate this contribution from the reviewer. The main results have been developed in more detail in the section ‘Materials and methods’ (pages 11 and 12), as follows:

**Primary outcomes**

The primary outcome is improvement in pelvic function following the proposed interventions, assessed in terms of reduction in symptoms and the impact of pelvic dysfunction, including urinary, colorectal-anal, and genital prolapse symptoms.

Assessment Criteria:

- Tool: Pelvic Floor Distress Inventory Questionnaire, short form (PFDI-20) 43. The PFDI-20 questionnaire uses a scoring system where each of the 20 items is rated from 0 to 4, with 0 meaning the symptom is not present and 1 to 4 indicating how much it bothers, from "not at all" to "quite a bit."

- Details: This questionnaire assesses the impact of pelvic dysfunction over the last 3 months and consists of three subscales:

Pelvic Organ Prolapse Impact Questionnaire (POPIQ-7): 6 items (questions 1-6), focusing on distress from pelvic organ prolapse.

Colon-Rectal-Anal Impact Questionnaire (CRAIQ-7): 8 items (questions 7-14), assesses colon-rectal-anal symptoms.

Urinary Impact Questionnaire (UIQ-7): 6 items (questions 15-20), evaluating urinary symptoms.

- Scoring: The maximum score is 300 points, with a maximum of 100 points for each subscale. A higher score indicates a greater negative impact on quality of life, so improvement is reflected in a reduction in the score, indicating fewer or less severe symptoms.

- Assessment Timing: It is measured at baseline and at follow-ups 15 days, 6 months, and 12 months post-intervention.

The secondary outcomes have been also expanded with a definition at the beginning of each secondary outcome, and it has been verified that the assessment criteria have been described: tools, procedure, and scoring.

All changes have been indicated in red in the manuscript.

**4. Data análisis section needs to be stratified in terms of primary endpoint análisis and secondary endpoint análisis.**

Following your recommendation, we have included the following text, stratifying it in terms of primary endpoint analysis and secondary endpoint analysis (page 17):

For the analysis of PFDI-20 scores (continuous variable) that determine improvement in pelvic function (primary outcome), ANOVA will be used if the data follow a normal distribution. If the data are not normally distributed, the Kruskal-Wallis test will be applied for intergroup evaluation in order to compare the three intervention groups (RF, PFMT and RF+PFMT) and determine which intervention is most effective in determining pelvic function. To evaluate changes within each group, the Student's t-test for paired samples will be used if the data are normal, or the Wilcoxon test if they are not, with the aim of evaluating the magnitude of improvement in pelvic function within each group over time. A repeated measures analysis will be performed using ANOVA to simultaneously analyse the effect between groups, the effect within groups and the group x time interaction to determine whether the effect of the intervention varies over time between groups.

Regarding the analysis of secondary variables, the comparative analysis between groups will be performed using ANOVA if the data are normal, or using the Kruskal-Wallis test if they are not, for continuous variables (Oxford scale for muscle strength, VHI, FSFI and S-BIS). For categorical variables (Sandvick severity test and dyspareunia using VAS), the chi-square test or Fisher's exact test will be used to analyse associations between groups. Intragroup evaluation will be performed using Student's t-test (normal data) or Wilcoxon's test (non-normal data) for continuous secondary variables, and McNemar's test will be applied to evaluate differences before and after the intervention for categorical secondary variables. Similarly, a repeated measures analysis will be performed using ANOVA to simultaneously analyse the effect between groups, the effect within groups and the group x time interaction to capture the short- and long-term effects of the interventions on the secondary outcomes.

**Reviewer #2:** **The Manuscript is a trial protocol only, possibly a part of doctoral thesis. Rationale of the study, inclusion criteria, intervention methods, statistical methodology of proposed data analysis is given in details, but results are not given it being just a trial protocol.**

**The limitations of the study are clearly identified as well.**

**The PI are requested to give more details of the scoring system used for evaluation of primary outcome. (PFDI-20 questionnaire and its scoring methods and calculations).**

We appreciate the reviewer's input. Further details on the scoring and calculation method for the PFDI-20 questionnaire have been provided (pages 11 and 12), as follows**:**

**Primary outcomes**

The primary outcome is improvement in pelvic function following the proposed interventions, assessed in terms of reduction in symptoms and the impact of pelvic dysfunction, including urinary, colorectal-anal, and genital prolapse symptoms.

Assessment Criteria:

- Tool: Pelvic Floor Distress Inventory Questionnaire, short form (PFDI-20) 43. The PFDI-20 questionnaire uses a scoring system where each of the 20 items is rated from 0 to 4, with 0 meaning the symptom is not present and 1 to 4 indicating how much it bothers, from "not at all" to "quite a bit."

- Details: This questionnaire assesses the impact of pelvic dysfunction over the last 3 months and consists of three subscales:

Pelvic Organ Prolapse Impact Questionnaire (POPIQ-7): 6 items (questions 1-6), focusing on distress from pelvic organ prolapse.

Colon-Rectal-Anal Impact Questionnaire (CRAIQ-7): 8 items (questions 7-14), assesses colon-rectal-anal symptoms.

Urinary Impact Questionnaire (UIQ-7): 6 items (questions 15-20), evaluating urinary symptoms.

- Scoring: The maximum score is 300 points, with a maximum of 100 points for each subscale. For each scale, calculate the mean score of the items, then multiply by 25 to get a score from 0 to 100. A higher score indicates a greater negative impact on quality of life, so improvement is reflected in a reduction in the score, indicating fewer or less severe symptoms.

- Assessment Timing: It is measured at baseline and at follow-ups 15 days, 6 months, and 12 months post-intervention.
