# Supplementary material for: Effectiveness of radiofrequency and exercise-based rehabilitation on symptoms associated with pelvic floor dysfunction in breast cancer patients: A study protocol
Source: PLoS One. 2025 Aug 29;20(8):e0330156. doi: 10.1371/journal.pone.0330156 (PMC12396660; doi:10.1371/journal.pone.0330156)
Supplement: Attachment — Submitted filename: Response_to_Reviewers_auresp_2.docx [file pone.0330156.s007.docx]

**Title:** Effectiveness of radiofrequency and exercise-based rehabilitation on symptoms associated with pelvic floor dysfunction in breast cancer patients: A study protocol.

**Article Type:** Study protocol

We wish to thank you greatly for the interest you have shown towards our study and for taking the time to review it.

All changes are indicated in red in the “Revised Manuscript with Track Changes” and here.

Here follows a point-by-point response to your comments:

**Reviewer Comments:**

**Reviewer #1: The software used to calculate the sample size "G*Power" is mentioned by the test statistics behind the sample size calculation is still missing. Ideally, the test statistics used to calculate the sample size needs to be used (and mentioned) to analyse the primary end point.**

The information requested by the reviewer has been included in the section “Sample size” of the Materials and Methods (page 9) as follows:

A sample size of 117 women (39 in each group) was obtained using the G*Power program and the one-way ANOVA statistical test with an effect size f: 0.25 (calculated from Cohen's d = 0.612 between extreme groups).

**The sentence, "A repeated measures analysis will be performed using ANOVA" can be shortened by writing, "repeated measures ANOVA".**

The change suggested by the reviewer has been made.
